# Time to benefit estimation in multicenter studies using flexible hazard shared frailty models

**DOI:** 10.1186/s12874-026-02816-1

**Published:** 2026-03-07

**Authors:** Mengyi Lu, Zhuoyue Wu, Yang Zhao, Fang Shao

**Affiliations:** 1 Department of Biostatistics, School of Public Health, Nanjing Medical University, Nanjing, 211166 China; 2 National Vaccine Innovation Platform, Nanjing Medical University, Nanjing, 211166 China; 3 Jiangsu Key Laboratory of Cancer Biomarkers, Prevention and Treatment, Collaborative Innovation Center for Cancer Personalized Medicine, Nanjing, 211166 China; 4 The First Affiliated Hospital of Nanjing Medical University, Nanjing, 211166 China

**Keywords:** Time to benefit, Clustered survival data, Shared frailty models, Marginal survival modeling, Uncertainty quantification

## Abstract

**Background:**

Time to benefit (TTB) has emerged as a clinically interpretable estimand for characterizing when treatment effects become meaningful over time. Unlike conventional survival summaries, TTB is implicitly defined through marginal differences in survival probabilities and is therefore highly sensitive to modeling assumptions. In multicenter studies involving clustered time-to-event data, unobserved heterogeneity and misspecification of the baseline hazard present additional challenges for coherent TTB estimation.

**Methods:**

We propose a unified framework for estimating TTB in clustered survival settings using marginal survival modeling with shared frailty. Specifically, TTB is defined on the marginal population scale by integrating over the frailty distribution, ensuring coherence between the estimand and its clinical interpretation. Both parametric and flexible spline-based baseline hazard models are evaluated. Uncertainty is quantified using the Delta method and Monte Carlo (MC)-based inference procedures. Extensive simulation studies are conducted to characterize estimator behavior under varying degrees of heterogeneity, censoring, and hazard misspecification. Furthermore, the framework is illustrated using data from the Systolic Blood Pressure Intervention Trial (SPRINT), a large multicenter randomized clinical trial.

**Results:**

Simulation results indicate that ignoring latent heterogeneity or misspecifying the baseline hazard can bias TTB estimation and produce miscalibrated confidence intervals, particularly under small absolute risk reduction thresholds. Flexible hazard models combined with MC-based inference yield more stable estimates and improved coverage in the presence of model misspecification. In the SPRINT application, TTB point estimates remained relatively consistent across modeling approaches, while statistically significant frailty effects revealed meaningful between-site heterogeneity, highlighting its importance for accurate uncertainty quantification.

**Conclusions:**

TTB is a model-sensitive implicit estimand; reliable estimation in clustered survival settings requires explicit alignment among the estimand definition, the survival model, and the inference strategy. The proposed framework provides a principled and practical approach to TTB estimation in multicenter studies, facilitating transparent and interpretable reporting of TTB in both clinical and real-world research contexts.

**Supplementary Information:**

The online version contains supplementary material available at 10.1186/s12874-026-02816-1.

## Introduction

Time-to-event analysis often requires insight into when meaningful clinical benefits are likely to emerge. This perspective has spurred growing interest in time to benefit (TTB), commonly defined as the earliest time point at which the treatment effect exceeds a prespecified threshold of clinical relevance typically expressed as an absolute risk reduction (ARR). TTB has been widely applied in preventive and chronic disease contexts to support individualized treatment decisions, particularly among older adults or high-risk populations, where trade-offs involving life expectancy, competing risks, and treatment burden must be carefully weighed [1, 2].

From a statistical standpoint, TTB differs fundamentally from conventional survival estimands. Unlike hazard ratios or survival probabilities evaluated at fixed time points, TTB is an implicitly defined, threshold-based quantity that depends on the entire shape of the survival functions over time. Consequently, its estimation is inherently sensitive to modeling assumptions regarding baseline hazards and treatment effects. Even minor deviations in estimated survival curves can result in substantial shifts in the estimated timing of benefit onset, a characteristic that distinguishes TTB from standard summaries of time-to-event data and presents unique challenges for reliable inference [3]. These challenges are especially pronounced in settings where treatment effects evolve nonlinearly over time or where survival dynamics deviate from simple parametric forms.

Despite its increasing use in applied research, most existing TTB analyses rely on pooled survival models that implicitly assume homogeneous populations and independent event times. These assumptions are often untenable in contemporary clinical research, where multicenter randomized trials and large observational studies frequently exhibit substantial between-center heterogeneity due to differences in patient characteristics, clinical practice patterns, and unmeasured institutional factors. In clustered survival settings, unobserved heterogeneity modifies marginal survival functions in a nonlinear manner, potentially advancing or delaying the time at which a clinically meaningful benefit threshold is crossed [4]. While shared frailty models are commonly employed to account for within-cluster dependence in survival analysis and are known to alter marginal survival behavior relative to naïve models [5–7], their implications for threshold-based estimands such as TTB have not been systematically evaluated.

These methodological concerns are well illustrated by large multicenter cardiovascular trials in which the timing of benefit is directly relevant to clinical practice. For example, the Systolic Blood Pressure Intervention Trial (SPRINT) demonstrated that intensive blood pressure control reduces major cardiovascular events, yet also revealed considerable heterogeneity in event rates across participating clinical sites [8, 9]. In such settings, standard TTB analyses based on pooled survival models implicitly assume homogeneous hazards and may obscure how latent heterogeneity and baseline hazard misspecification influence the estimated onset of benefit. This raises concerns about the robustness and interpretability of TTB estimates when used to inform time-sensitive clinical decisions.

In this work, we address these limitations by developing a unified framework for estimating TTB in clustered survival data. Our approach defines TTB on the marginal population scale, explicitly accounting for unobserved heterogeneity through shared frailty modeling while accommodating complex and potentially misspecified hazard structures using flexible baseline hazard specifications. Recognizing that TTB is an implicitly defined estimand with nonstandard sampling properties, we further develop complementary inferential procedures to quantify uncertainty in its estimation. Through simulation studies and application to a large multicenter randomized clinical trial, we illustrate how modeling choices influence the estimated timing of benefit and provide practical guidance for robust TTB analysis in heterogeneous survival settings.

The remainder of this article is organized as follows. Sect. “Method” introduces the proposed modeling framework and inferential procedures. Sect. “Simulation study” presents the design and results of simulation studies. Sect. “Real data analysis: the SPRINT trial” applies the proposed methods to the SPRINT trial. Sect. “Discussion” concludes with a discussion of methodological implications and directions for future research.

## Method

### Target estimand: TTB in clustered survival settings

We consider TTB as the primary estimand of interest. Let *S*_1_(*t*) and *S*_0_(*t*) denote the marginal survival functions under treatment and control, respectively. TTB is defined as the earliest time point at which the absolute risk reduction between treatment groups exceeds a prespecified clinically meaningful threshold *δ*, that is [10],

1 $$TTB=\mathrm{inf}\{t>0 :{S}_{1}(t)-{S}_{0}(t)\ge \delta \}$$

This definition highlights two features that distinguish TTB from conventional survival estimands. First, TTB is a derived quantity, implicitly defined through the entire survival trajectory rather than as a direct model parameter. Second, TTB is defined on the marginal population scale, making it sensitive to both baseline hazard specification and unobserved heterogeneity. These features motivate the modeling and inferential strategies described below.

### Shared frailty framework for marginal survival modeling

#### Conditional hazard specification

To account for within-center dependence in multicenter studies, we adopt a shared frailty survival model [5]. Let *i* index individual, *j* index corresponding center, and *a* ∈ {0, 1} index corresponding treatment group. The conditional hazard function for individual *i* in center *j* under treatment *a* is specified as

2 $${h}_{ija}(t\mid a, {x}_{ij}, {w}_{j})={\omega }_{j}\cdot {h}_{0a}(t)\mathrm{exp}({x}_{ij}^{T}\beta )$$

where *h*_0*a*_(*t*) denotes the treatment-specific baseline hazard, *x*_*ij*_ is a vector of covariates, and *β* is the corresponding regression coefficient vector. *x*_*ij*_ and *β* are omitted in the followings for RCT data. The frailty term *ω*_*j*_, shared by all individuals within center *j*, induces correlation among their event times, allows for non-proportional hazards at the group level, and captures unobserved center-level heterogeneity through a multiplicative modification of the hazard.

#### Marginal survival under Gamma and log-normal frailty

Because TTB is defined in terms of marginal survival functions, inference requires integrating out the frailty term *ω*_*j*_. Under the shared frailty framework, the marginal survival function for treatment group *a*, *S*_*a*_(*t*), is obtained by taking the expectation of the conditional survival over the frailty distribution without adjusting *x*_*ij*_.

3 $${S}_{a}(t) ={ E}_{\omega } \left[\text{ exp}(-\omega {\int }_{0}^{t}{h}_{0a}(u)du)\right]={E}_{\omega }[\mathrm{exp}(-\omega {H}_{0a}(t))]$$

We consider two standard specifications for frailty, Gamma frailty and log-normal frailty, which represent different assumptions about the distributional shape of latent heterogeneity [11]. For a Gamma frailty $\omega \sim Gamma(1/\theta , 1/\theta )$, where the parameter $\theta>0$ governs the variance this specification, the expectation admits a closed -form expression, yielding an explicit marginal survival function

4 $${S}_{a}(t)={(1+\theta {H}_{0a}(t))}^{-1/\theta }$$

where larger values of *θ* correspond to greater between-cluster heterogeneity and induce stronger divergence between conditional and marginal survival functions. For log-normal frailty, where ln(*ω*) ∼ *N*(0, *θ*), the marginal survival function lacks a closed-form expression and is evaluated numerically using Gaussian-Hermite quadrature. Within this framework, the frailty term functions as a mechanism for marginalization rather than solely as a random effect. Variation in the frailty parameter *θ* alters the shape of the marginal survival curves and can substantially influence the estimated time at which the survival difference crosses the TTB threshold.

### Baseline hazard specification

#### Parametric Weibull baseline

As a parametric benchmark, the baseline hazard is modeled using a Weibull specification [12],

5 $${h}_{0}\left(t\right)=\alpha \gamma {t}^{\gamma -1}$$

where *α* and *γ* are scale and shape parameters, respectively. Although computationally convenient, this model assumes monotonic hazard behavior and may fail to adequately capture complex temporal dynamics relevant to TTB estimation.

#### Flexible spline-based baseline hazards

To reduce the impact of baseline hazard misspecification on TTB estimation, flexible spline-based models are adopted. Specifically, for each treatment group *a*, the logarithm of the baseline hazard is modeled as

6 $$\mathrm{ln}\,{h}_{0a}\left(t\right)={\beta }_{0a}+\sum\nolimits_{k=1}^{K}{\beta }_{ka}{B}_{ka}(t)$$

where *B*_*ka*_(‧) denote spline basis functions and *β*_*ka*_ are the corresponding coefficients, *β*_0*a*_ denotes the intercept term controlling the overall level of the baseline hazard, and *k* is the number of spline basis functions used to represent the baseline hazard. We consider penalized M-splines (PMS), penalized thin-plate regression splines (PTPRS), and natural cubic splines (NCS), each providing different smoothness and boundary behavior [13, 14]. All spline-based hazards are estimated via penalized maximum likelihood, with smoothing parameters selected using cross-validation or restricted maximum likelihood. These flexible specifications mitigate the risk that overly restrictive hazard assumptions cause survival curves to separate too early or too late, thereby improving the stability of TTB estimation.

### Estimation of TTB

Given estimates of the marginal survival functions $\hat{S}_{1}(t)$ and $\hat{S}_{0}(t)$, TTB is estimated as

7 $$\widehat{\tau }=\mathrm{inf}\{t :{\widehat{S}}_{1}(t)-{\widehat{S}}_{0}(t)\ge\Delta \}$$

where Δ > 0 denotes a prespecified absolute risk reduction threshold defining clinical relevance. TTB is obtained numerically using root-finding algorithms applied to the estimated marginal survival curves.

### Statistical inference for TTB as a derived quantity

#### Delta-method inference

Let $\widehat{\varphi }$ denote the vector of all estimated model parameters, including regression, spline, and frailty parameters. The Delta method approximates the variance of TTB by linearizing the threshold-crossing condition with respect to $\widehat{\varphi }$ [15]. Here linearization is performed with respect to the full parameter vector $\widehat{\varphi }$ rather than the frailty variance parameter $\hat{\theta}$ alone.

8 $$\widehat{Var}(\mathrm{ln}(\widehat{\tau }))\approx {\left(\frac{\partial \text{ ln}(\widehat{\tau })}{\partial \widehat{\varphi } }\right)}^{T}{\widehat{\Sigma }}_{\widehat{\varphi }}\left(\frac{\partial \text{ ln}(\widehat{\tau })}{\partial \widehat{\varphi }}\right)$$

${\widehat{\Sigma }}_{\widehat{\varphi }}$ is the estimated covariance matrix of the parameters. The logarithmic transformation is used to improve normal approximation and to ensure positivity of the TTB estimator. Confidence intervals are constructed on the logarithmic scale and subsequently back-transformed to the original scale. The 95% CI for *τ* is then:

9 $$\left(\mathrm{exp}\left(\mathrm{ln}\left(\widehat{\tau }\right)-{z}_{0.975}\sqrt{\widehat{Var}(\mathrm{ln}(\widehat{\tau }))}\right),\text{ exp}\left(\mathrm{ln}\left(\widehat{\tau }\right)+{z}_{0.975}\sqrt{\widehat{Var}(\mathrm{ln}(\widehat{\tau }))}\right)\right),$$

where *z*_0.975_ is the 97.5% quantile of the standard normal distribution.

#### Monte Carlo-based inference

To reduce reliance on a normal approximation for the TTB estimator $\widehat{\tau }$ itself, we additionally implement a Monte Carlo-based procedure [16, 17]. This approach is widely used for inference on nonlinear functionals of estimated model parameters and relies on the first-level asymptotic normality of the estimated parameter vector $\widehat{\varphi }$. In particular, it avoids the additional linearization inherent in the Delta method when mapping from $\varphi$ to $\tau$. Specifically, parameter draws are generated from the estimated joint asymptotic distribution of the model parameters $\widehat{\varphi }$, and the TTB is recomputed for each draw using the same numerical routine as in the point estimation. The empirical quantiles of the resulting TTB distribution provide simulation-based confidence intervals, offering improved robustness in finite samples and in settings where the sampling distribution of TTB may exhibit nonlinearity-induced skewness.

The procedure is as follows:

1. Generate parameter draws ${\varphi }^{\left(b\right)}$ from the estimated asymptotic multivariate normal distribution $N\left(\widehat{\varphi },{\widehat{\Sigma }}_{\widehat{\varphi }}\right)$.
2. Compute ${\tau }^{\left(b\right)}$ for each draw using Eq. 7.
3. Estimate $$\widehat{\tau }=\mathrm{median}\left({\tau }^{\left(b\right)}\right)$$ from $${\left\{{\tau }^{\left(b\right)}\right\}}_{b=1}^{B}$$.

The 95% CI for $\tau$ is $\left({\tau }_{0.025}^{\left(b\right)}, {\tau }_{0.975}^{\left(b\right)}\right)$ using 2.5% and 97.5% quantiles.

## Simulation study

### Simulation design

Simulation studies were conducted to evaluate the finite-sample operating characteristics of TTB estimation under clustered survival settings. The design follows the modeling framework in Sect. “Method” and targets the sensitivity of TTB to frailty, baseline hazard specification, censoring, and inference strategy. Parameters were empirically calibrated using estimates from preliminary shared frailty models fitted to the SPRINT dataset. These values were used only to define realistic magnitudes of hazards, frailty variance, and censoring rates, and were fixed prior to simulation. Here, the frailty variance refers to the parameter governing the variability of the shared frailty term (*θ* for Gamma frailty and the variance parameter of the log-normal distribution), which was held fixed within each simulation scenario.

### Data generation mechanisms

Clustered survival data were generated under a shared frailty structure with varying numbers of clusters and total sample sizes of 5,000 and 10,000. Specifically, the number of clusters was fixed across simulation replicates, with cluster sizes determined implicitly by the total sample size. Treatment assignment was balanced at the cluster level. Frailty terms were generated from gamma or log-normal distributions. Conditional on frailty, event times were generated from the hazard in Eq. (2). Independent right-censoring times were generated from Weibull distributions to achieve censoring rates of 0%, 30%, 60%, and 90%. Event times were obtained using inverse transform sampling when closed-form inverses were available.

### Simulation scenarios

Two primary simulation scenarios were considered, each representing a distinct class of baseline hazard behavior commonly encountered in multicenter clinical studies.

#### Scenario I: Weibull baseline hazards

Both treatment and control group hazards followed Weibull distributions. The conditional baseline hazard functions were specified as

10 $${h}_{0a}(t)={\lambda }_{a}{\rho }_{a}{t}^{{\rho }_{a}-1}$$

Parameter values were selected based on empirically plausible magnitudes derived from preliminary Weibull shared frailty modeling, ensuring realistic hazard function behavior while remaining fixed throughout the simulation study. For Gamma frailty, parameters were set to achieve true TTB values of 1.874, 2.156, and 2.388 for ARR thresholds of 0.002, 0.005, and 0.010, respectively. For log-normal frailty, parameters were set to analogous values, yielding corresponding TTB estimates of 1.932, 2.281, and 2.493 under the same ARR thresholds.

#### Scenario II: piecewise Weibull baseline hazards

Scenario II was designed to introduce baseline hazard misspecification relative to the parametric Weibull model assumed in the analysis. The control-group hazard remained Weibull, as in Scenario I, whereas the treatment-group hazard followed a piecewise Weibull structure with a change point at six months, thereby deviating from the proportional hazards assumption.

11 $${h}_{1}(t)=\left\{\! \begin{array}{l} {\lambda }_{0}{\rho }_{0}{t}^{{\rho }_{0}-1}, t \in [0, 1]\\ {\lambda }_{1}{\rho }_{1}{t}^{{\rho }_{1}-1}, t>1\end{array}\right.$$

with parameters chosen to induce non-Weibull hazard behavior over time. Frailty parameters for both Gamma and log-normal distributions were identical to those used in Scenario I. For Gamma frailty, the true TTB values were 2.685 for ARR values of 0.002, 0.005, and 0.010, respectively. Corresponding values under log-normal frailty were 2.757.

### Simulation conditions, competing methods, and evaluation metrics

Across both scenarios, simulation conditions varied according to:

1. Censoring rates: 0%, 30%, 60%, and 90%, achieved using Weibull censoring distributions with parameters reported in Supplementary Table S1.
2. Monte Carlo resamples: *B* = 1000 and *B* = 2000 for MC-based interval estimation. Both values were considered to assess the stability of Monte Carlo-based interval estimation with respect to the number of resamples.

The performance of TTB estimation was evaluated using the Delta method and the MC-based method described in Sect. “Statistical inference for TTB as a derived quantity”. Five modeling approaches were compared: the parametric Weibull model without frailty (WnoF), the parametric Weibull shared frailty model (WF), and three spline-based shared frailty models using PMS, PTPRS, and NCS. Both Gamma and log-normal frailty distributions were considered.

Evaluation metrics focused on both point and interval estimation. Point estimation accuracy was assessed using bias, mean absolute error (MAE), and root mean squared error (RMSE). Interval estimation performance was evaluated using coverage probability (CP), out-of-lower-limit probability (OLLP), out-of-upper-limit probability (OULP), and average interval length for nominal 95% confidence intervals. OLLP and OULP denote the probabilities that the true TTB lies below the lower confidence limit and above the upper confidence limit, respectively. Computation time was recorded for all methods.

### Simulation results

The simulation results are presented in Tables 1, 2, 3 and 4, Supplementary Tables S2–S13, and Supplementary Figures S1–S8.

**Table 1:** TTB point estimation results for simulation scenario I with the Gamma frailty and 1,000 replicates

				*N* = 5,000				*N* = 10,000			
Cesnoring	Model	Method	ARR	Bias	MAE	RMSE	Time	Bias	MAE	RMSE	Time
0%	WnoF	Delta	0.002	−0.053	0.372	0.460	1 min	−0.039	0.262	0.325	1 min
		MC		−0.064	0.372	0.459	30 min	−0.047	0.263	0.325	1hrs30min
	WF	Delta		−0.001	0.339	0.417	26 min	0.004	0.239	0.296	1hrs52min
		MC		−0.023	0.339	0.417	2hrs55min	−0.012	0.239	0.295	2hrs21min
	PMS	Delta		−0.107	0.631	0.772	3hrs30min	−0.092	0.482	0.593	8hrs16min
		MC		−0.063	0.610	0.748	6hrs37min	−0.057	0.467	0.575	10hrs24min
	PTPRS	Delta		−0.048	0.482	0.602	13hrs43min	−0.046	0.360	0.454	15hrs49min
	NCS	Delta		−0.132	0.514	0.620	4hrs50min	−0.148	0.396	0.479	4hrs4min
	WnoF	Delta	0.005	−0.043	0.272	0.337	–––	−0.034	0.191	0.237	–––
		MC		−0.050	0.273	0.338	–––	−0.040	0.191	0.238	–––
	WF	Delta		0.001	0.247	0.306	–––	0.004	0.174	0.215	–––
		MC		−0.015	0.248	0.306	–––	−0.008	0.175	0.215	–––
	PMS	Delta		−0.025	0.405	0.509	–––	−0.012	0.287	0.362	–––
		MC		0.001	0.394	0.496	–––	0.005	0.280	0.353	–––
	PTPRS	Delta		−0.022	0.327	0.411	–––	−0.021	0.237	0.300	–––
	NCS	Delta		−0.051	0.364	0.446	–––	−0.052	0.265	0.328	–––
	WnoF	Delta	0.010	−0.031	0.210	0.261	–––	−0.024	0.147	0.182	–––
		MC		−0.034	0.210	0.262	–––	−0.027	0.147	0.183	–––
	WF	Delta		0.001	0.193	0.239	–––	0.003	0.135	0.167	–––
		MC		−0.012	0.193	0.239	–––	−0.007	0.136	0.168	–––
	PMS	Delta		−0.002	0.278	0.353	–––	0.007	0.189	0.240	–––
		MC		0.012	0.276	0.349	–––	0.015	0.188	0.239	–––
	PTPRS	Delta		−0.013	0.238	0.300	–––	−0.010	0.170	0.215	–––
	NCS	Delta		−0.020	0.273	0.338	–––	−0.014	0.192	0.241	–––
90%	WnoF	Delta	0.002	−0.064	0.542	0.676	1 min	−0.046	0.397	0.494	1 min
		Delta		−0.058	0.536	0.669	1hrs38min	−0.041	0.394	0.491	1hrs34min
	WF	Delta		−0.055	0.537	0.670	19 min	−0.040	0.394	0.491	1hrs33min
		MC		−0.085	0.547	0.681	3hrs42min	−0.047	0.398	0.495	2hrs15min
	PMS	Delta		−0.123	0.650	0.796	1hrs31min	−0.057	0.467	0.585	1hrs9min
		MC		−0.083	0.638	0.785	15hrs10min	−0.027	0.452	0.569	15hrs37min
	PTPRS	Delta		−0.070	0.557	0.700	24hrs18min	−0.046	0.411	0.518	40hrs21min
	NCS	MC		−0.124	0.637	0.794	4hrs29min	−0.063	0.466	0.596	5hrs36min
	WnoF	Delta	0.005	−0.029	0.374	0.471	–––	−0.019	0.265	0.334	–––
		Delta		−0.030	0.366	0.463	–––	−0.018	0.262	0.330	–––
	WF	Delta		−0.027	0.372	0.469	–––	−0.017	0.264	0.332	–––
		MC		−0.044	0.374	0.472	–––	−0.021	0.263	0.332	–––
	PMS	Delta		−0.027	0.420	0.527	–––	−0.006	0.293	0.373	–––
		MC		−0.003	0.416	0.524	–––	0.006	0.292	0.371	–––
	PTPRS	Delta		−0.027	0.379	0.477	–––	−0.017	0.271	0.342	–––
	NCS	MC		−0.050	0.427	0.536	–––	−0.029	0.308	0.390	–––
	WnoF	Delta	0.010	−0.010	0.278	0.353	–––	−0.004	0.192	0.244	–––
		Delta		−0.019	0.273	0.345	–––	−0.008	0.190	0.242	–––
	WF	Delta		−0.011	0.278	0.352	–––	−0.005	0.192	0.243	–––
		MC		−0.023	0.277	0.350	–––	−0.011	0.192	0.243	–––
	PMS	Delta		0.000	0.294	0.371	–––	0.003	0.211	0.266	–––
		MC		0.008	0.290	0.367	–––	0.011	0.209	0.263	–––
	PTPRS	Delta		−0.009	0.281	0.356	–––	−0.006	0.196	0.248	–––
	NCS	MC		−0.026	0.312	0.390	–––	−0.017	0.227	0.284	–––

Metrics are calculated based on the logarithms of TTB estimates. The number of MC samples is 2,000. For MC method, the estimates are the median of the MC samples. Time is for all ARR values for each model

*ARR* absolute relative risk, *MAE* mean absolute error, *RMSE* root mean squared error, *MC* Monte Carlo, *NCS* natural cubic spline, *PMS* penalized M-spline, *PTPRS* penalized thin plate regression spline, *SPRINT* Systolic Blood Pressure Intervention Trial, *TTB* time to benefit, *WF* Weibull model with shared frailty, *WnoF* Weibull model without shared frailty

**Table 2:** TTB interval estimation results for simulation scenario I with the Gamma frailty and 1,000 replicates

				*N* = 5,000				*N* = 10,000			
Cesnoring	Model	Method	ARR	CP	OLLP	OULP	length	CP	OLLP	OULP	length
0%	WnoF	Delta	0.002	0.924	0.028	0.048	1.788	0.941	0.023	0.036	1.287
		MC		0.944	0.024	0.032	1.731	0.954	0.019	0.027	1.267
	WF	Delta		0.929	0.035	0.035	1.633	0.946	0.027	0.028	1.175
		MC		0.947	0.023	0.029	1.581	0.953	0.020	0.027	1.151
	PMS	Delta		0.812	0.098	0.089	3.006	0.865	0.079	0.056	2.304
		MC		0.933	0.034	0.033	2.536	0.946	0.030	0.023	2.025
	PTPRS	Delta		0.911	0.055	0.034	2.534	0.939	0.039	0.022	1.928
	NCS	Delta		0.843	0.045	0.112	2.388	0.880	0.027	0.093	1.853
	WnoF	Delta	0.005	0.931	0.023	0.046	1.311	0.946	0.018	0.036	0.937
		MC		0.952	0.022	0.026	1.297	0.955	0.018	0.027	0.939
	Weibull	Delta		0.939	0.027	0.033	1.197	0.949	0.026	0.026	0.856
		MC		0.950	0.022	0.027	1.176	0.954	0.020	0.026	0.843
	PMS	Delta		0.874	0.074	0.051	1.959	0.913	0.062	0.025	1.445
		MC		0.934	0.038	0.028	1.813	0.943	0.036	0.020	1.364
	PTPRS	Delta		0.933	0.045	0.022	1.696	0.946	0.032	0.022	1.266
	NCS	Delta		0.883	0.044	0.073	1.751	0.920	0.029	0.051	1.320
	WnoF	Delta	0.010	0.936	0.023	0.041	1.019	0.945	0.020	0.035	0.725
		MC		0.955	0.020	0.025	1.014	0.955	0.020	0.025	0.728
	Weibull	Delta		0.941	0.029	0.029	0.934	0.952	0.024	0.025	0.666
		MC		0.956	0.018	0.025	0.928	0.952	0.023	0.026	0.661
	PMS	Delta		0.910	0.057	0.033	1.353	0.942	0.042	0.016	0.971
		MC		0.938	0.035	0.027	1.329	0.954	0.028	0.017	0.958
	PTPRS	Delta		0.940	0.035	0.025	1.225	0.959	0.021	0.020	0.904
	NCS	Delta		0.900	0.049	0.051	1.327	0.936	0.033	0.031	0.978
90%	WnoF	Delta	0.002	0.873	0.076	0.051	2.656	0.907	0.048	0.045	1.970
		MC		0.937	0.033	0.030	2.439	0.944	0.025	0.031	1.845
	Weibull	Delta		0.872	0.078	0.050	2.702	0.904	0.052	0.044	1.954
		MC		0.936	0.030	0.034	2.483	0.946	0.020	0.034	1.861
	PMS	Delta		0.811	0.105	0.085	3.187	0.873	0.083	0.044	2.318
		MC		0.944	0.027	0.029	2.800	0.950	0.023	0.027	2.153
	PTPRS	Delta		0.855	0.081	0.064	2.687	0.896	0.057	0.047	1.999
	NCS	Delta		0.747	0.169	0.084	2.408	0.828	0.126	0.046	2.090
	WnoF	Delta	0.005	0.905	0.057	0.038	1.842	0.931	0.036	0.033	1.332
		MC		0.935	0.030	0.035	1.785	0.943	0.028	0.029	1.294
	Weibull	Delta		0.905	0.057	0.038	1.921	0.932	0.036	0.032	1.323
		MC		0.939	0.026	0.035	1.819	0.949	0.019	0.032	1.319
	PMS	Delta		0.871	0.079	0.050	2.161	0.908	0.061	0.031	1.436
		MC		0.940	0.034	0.026	2.021	0.952	0.027	0.021	1.471
	PTPRS	Delta		0.900	0.056	0.044	1.853	0.930	0.039	0.031	1.340
	NCS	Delta		0.802	0.123	0.075	1.772	0.842	0.101	0.057	1.361
	WnoF	Delta	0.010	0.930	0.034	0.036	1.370	0.944	0.029	0.027	0.968
		MC		0.936	0.029	0.035	1.382	0.945	0.027	0.028	0.967
	Weibull	Delta		0.929	0.034	0.037	1.403	0.945	0.029	0.026	0.964
		MC		0.941	0.024	0.035	1.426	0.952	0.020	0.028	0.989
	PMS	Delta		0.907	0.059	0.034	1.584	0.913	0.060	0.027	1.065
		MC		0.948	0.026	0.025	1.551	0.951	0.031	0.019	1.094
	PTPRS	Delta		0.925	0.033	0.042	1.385	0.936	0.036	0.028	0.984
	NCS	Delta		0.797	0.115	0.087	1.257	0.848	0.091	0.061	1.044

Metrics are calculated based on the logarithms of TTB estimates. The number of MC samples is 2,000

*ARR* absolute relative risk, *CP* coverage probability, *OLLP* out-of-lower-limit probability, *OULP* out-of-upper-limit probability, *MAE* mean absolute error, *RMSE* root mean squared error, *MC* Monte Carlo, *NCS* natural cubic spline, *PMS* penalized M-spline, *PTPRS* penalized thin plate regression spline, *SPRINT* Systolic Blood Pressure Intervention Trial, *TTB* time to benefit, *WF* Weibull model with shared frailty, *WnoF* Weibull model without shared frailty

**Table 3:** TTB point estimation results for simulation scenario II with the Gamma frailty and 1,000 replicates

				*N* = 5,000				*N* = 10,000			
Cesnoring	Model	Method	ARR	Bias	MAE	RMSE	Time	Bias	MAE	RMSE	Time
0%	WnoF	Delta	0.002	−0.323	0.455	0.561	1 min	−0.307	0.366	0.446	1 min
		MC		−0.334	0.460	0.566	1hrs32min	−0.315	0.371	0.451	1hrs33min
	WF	Delta		−0.274	0.411	0.502	26 min	−0.266	0.327	0.400	1hrs51min
		MC		−0.296	0.422	0.514	2hrs23min	−0.283	0.337	0.411	3hrs50min
	PMS	Delta		−0.203	0.597	0.771	3hrs28min	−0.164	0.439	0.571	9hrs45min
		MC		−0.167	0.571	0.740	6hrs14min	−0.134	0.421	0.547	12hrs32min
	PTPRS	Delta		−0.233	0.508	0.660	17hrs50min	−0.208	0.392	0.510	19hrs21min
	NCS	Delta		−0.249	0.549	0.665	5hrs58min	−0.245	0.431	0.525	5hrs19min
	WnoF	Delta	0.005	−0.181	0.310	0.382	–––	−0.171	0.237	0.292	–––
		MC		−0.189	0.313	0.386	–––	−0.177	0.240	0.295	–––
	WF	Delta		−0.140	0.276	0.339	–––	−0.135	0.207	0.257	–––
		MC		−0.156	0.282	0.346	–––	−0.147	0.214	0.264	–––
	PMS	Delta		−0.054	0.379	0.489	–––	−0.030	0.260	0.334	–––
		MC		−0.034	0.371	0.475	–––	−0.016	0.256	0.326	–––
	PTPRS	Delta		−0.094	0.330	0.423	–––	−0.076	0.240	0.306	–––
	NCS	Delta		−0.083	0.368	0.452	–––	−0.075	0.268	0.332	–––
	WnoF	Delta	0.010	−0.102	0.226	0.280	–––	−0.095	0.166	0.205	–––
		MC		−0.105	0.227	0.282	–––	−0.098	0.167	0.206	–––
	WF	Delta		−0.072	0.204	0.252	–––	−0.069	0.147	0.183	–––
		MC		−0.086	0.207	0.256	–––	−0.079	0.151	0.187	–––
	PMS	Delta		−0.007	0.262	0.335	–––	0.006	0.176	0.224	–––
		MC		0.005	0.261	0.331	–––	0.014	0.176	0.223	–––
	PTPRS	Delta		−0.039	0.235	0.299	–––	−0.027	0.167	0.211	–––
	NCS	Delta		−0.016	0.269	0.333	–––	−0.006	0.189	0.236	–––
90%	WnoF	Delta	0.002	−0.253	0.560	0.712	1 min	−0.228	0.416	0.535	2 min
		Delta		−0.251	0.551	0.702	1hrs40min	−0.225	0.412	0.530	1hrs35min
	WF	Delta		−0.245	0.554	0.704	19 min	−0.221	0.411	0.529	1hrs32min
		MC		−0.273	0.568	0.721	3hrs6min	−0.228	0.413	0.534	3hrs44min
	PMS	Delta		−0.194	0.626	0.801	1hrs33min	−0.132	0.445	0.586	1hrs14min
		MC		−0.177	0.601	0.774	4hrs10min	−0.115	0.432	0.568	4hrs21min
	PTPRS	Delta		−0.225	0.579	0.757	29hrs20min	−0.179	0.426	0.566	45hrs33min
	NCS	MC		−0.227	0.634	0.852	6hrs47min	−0.129	0.441	0.629	6hrs18min
	WnoF	Delta	0.005	−0.109	0.382	0.482	–––	−0.096	0.272	0.347	–––
		Delta		−0.113	0.377	0.476	–––	−0.098	0.270	0.344	–––
	WF	Delta		−0.105	0.381	0.481	–––	−0.094	0.270	0.344	–––
		MC		−0.122	0.385	0.486	–––	−0.100	0.269	0.344	–––
	PMS	Delta		−0.035	0.394	0.510	–––	−0.015	0.271	0.351	–––
		MC		−0.030	0.388	0.500	–––	−0.006	0.271	0.349	–––
	PTPRS	Delta		−0.077	0.383	0.489	–––	−0.054	0.262	0.336	–––
	NCS	MC		−0.053	0.398	0.525	–––	−0.023	0.272	0.351	–––
	WnoF	Delta	0.010	−0.039	0.283	0.357	–––	−0.032	0.196	0.249	–––
		Delta		−0.046	0.279	0.353	–––	−0.036	0.194	0.247	–––
	WF	Delta		−0.039	0.282	0.356	–––	−0.033	0.195	0.248	–––
		MC		−0.049	0.284	0.358	–––	−0.039	0.196	0.249	–––
	PMS	Delta		0.010	0.282	0.363	–––	0.009	0.201	0.251	–––
		MC		0.015	0.278	0.356	–––	0.018	0.200	0.251	–––
	PTPRS	Delta		−0.020	0.279	0.352	–––	−0.013	0.193	0.244	–––
	NCS	MC		−0.012	0.299	0.377	–––	−0.005	0.214	0.267	–––

Metrics are calculated based on the logarithms of TTB estimates. The number of MC samples is 2,000. For MC method, the estimates are the median of the MC samples. Time is for all ARR values for each model

*ARR* absolute relative risk, *MAE* mean absolute error, *RMSE* root mean squared error, *MC* Monte Carlo, *NCS* natural cubic spline, *PMS* penalized M-spline, *PTPRS* penalized thin plate regression spline, *SPRINT* Systolic Blood Pressure Intervention Trial, *TTB* time to benefit, *WF* Weibull model with shared frailty, *WnoF* Weibull model without shared frailty

**Table 4:** TTB interval estimation results for simulation scenario II with the Gamma frailty and 1,000 replicates

				*N* = 5,000				*N* = 10,000			
Cesnoring	Model	Method	ARR	CP	OLLP	OULP	length	CP	OLLP	OULP	length
0%	WnoF	Delta	0.002	0.886	0.005	0.109	1.795	0.855	0.002	0.143	1.291
		MC		0.908	0.001	0.091	1.738	0.873	0.002	0.125	1.272
	WF	Delta		0.886	0.005	0.109	1.645	0.855	0.003	0.142	1.183
		MC		0.898	0.000	0.102	1.594	0.851	0.002	0.147	1.162
	PMS	Delta		0.845	0.076	0.079	2.835	0.901	0.055	0.044	2.165
		MC		0.935	0.028	0.037	2.491	0.947	0.025	0.028	1.956
	PTPRS	Delta		0.914	0.037	0.049	2.564	0.942	0.026	0.032	1.970
	NCS	Delta		0.829	0.042	0.129	2.428	0.871	0.026	0.103	1.888
	WnoF	Delta	0.005	0.900	0.009	0.091	1.321	0.894	0.003	0.103	0.944
		MC		0.930	0.004	0.066	1.305	0.913	0.003	0.084	0.945
	Weibull	Delta		0.911	0.006	0.083	1.208	0.902	0.006	0.092	0.864
		MC		0.924	0.003	0.073	1.188	0.900	0.004	0.096	0.853
	PMS	Delta		0.889	0.069	0.042	1.854	0.926	0.055	0.019	1.345
		MC		0.939	0.032	0.029	1.758	0.951	0.031	0.018	1.299
	PTPRS	Delta		0.932	0.033	0.035	1.685	0.953	0.023	0.024	1.247
	NCS	Delta		0.878	0.051	0.071	1.748	0.916	0.032	0.052	1.314
	WnoF	Delta	0.010	0.920	0.012	0.068	1.026	0.925	0.007	0.068	0.730
		MC		0.941	0.009	0.050	1.019	0.938	0.006	0.056	0.732
	Weibull	Delta		0.935	0.014	0.051	0.942	0.924	0.010	0.066	0.672
		MC		0.944	0.006	0.050	0.937	0.927	0.006	0.067	0.669
	PMS	Delta		0.917	0.054	0.029	1.281	0.947	0.041	0.012	0.908
		MC		0.944	0.031	0.025	1.274	0.957	0.029	0.014	0.906
	PTPRS	Delta		0.947	0.029	0.024	1.205	0.965	0.017	0.018	0.879
	NCS	Delta		0.904	0.052	0.044	1.303	0.935	0.041	0.024	0.955
90%	WnoF	Delta	0.002	0.907	0.036	0.057	2.668	0.917	0.026	0.057	1.941
		MC		0.938	0.017	0.045	2.476	0.928	0.013	0.059	1.841
	Weibull	Delta		0.910	0.036	0.054	2.662	0.916	0.026	0.058	1.920
		MC		0.933	0.016	0.051	2.514	0.924	0.008	0.068	1.858
	PMS	Delta		0.831	0.088	0.081	3.092	0.893	0.069	0.038	2.211
		MC		0.938	0.023	0.039	2.745	0.952	0.020	0.028	2.168
	PTPRS	Delta		0.888	0.046	0.066	2.739	0.908	0.039	0.053	2.005
	NCS	Delta		0.742	0.137	0.121	2.274	0.824	0.111	0.065	1.935
	WnoF	Delta	0.005	0.926	0.033	0.041	1.855	0.930	0.025	0.045	1.332
		MC		0.938	0.020	0.042	1.819	0.935	0.018	0.047	1.301
	Weibull	Delta		0.924	0.035	0.041	1.889	0.931	0.025	0.044	1.320
		MC		0.942	0.016	0.042	1.851	0.940	0.012	0.048	1.325
	PMS	Delta		0.885	0.074	0.041	2.104	0.920	0.062	0.018	1.400
		MC		0.946	0.026	0.028	2.014	0.950	0.028	0.022	1.456
	PTPRS	Delta		0.917	0.041	0.042	1.871	0.938	0.036	0.026	1.315
	NCS	Delta		0.808	0.127	0.065	1.619	0.856	0.100	0.044	1.419
	WnoF	Delta	0.010	0.935	0.028	0.037	1.387	0.945	0.027	0.028	0.979
		MC		0.937	0.025	0.038	1.417	0.943	0.024	0.033	0.983
	Weibull	Delta		0.935	0.029	0.036	1.451	0.944	0.027	0.029	0.973
		MC		0.949	0.013	0.038	1.469	0.949	0.017	0.034	1.004
	PMS	Delta		0.915	0.055	0.030	2.670	0.924	0.050	0.026	1.014
		MC		0.948	0.027	0.025	1.599	0.948	0.032	0.020	1.087
	PTPRS	Delta		0.933	0.032	0.035	1.377	0.945	0.030	0.025	0.971
	NCS	Delta		0.808	0.110	0.082	1.259	0.871	0.076	0.053	1.021

Metrics are calculated based on the logarithms of TTB estimates. The number of MC samples is 2,000

*ARR* absolute relative risk, *CP* coverage probability, *OLLP* out-of-lower-limit probability, *OULP* out-of-upper-limit probability, *MAE* mean absolute error, *RMSE* root mean squared error, *MC* Monte Carlo, *NCS* natural cubic spline, *PMS* penalized M-spline, *PTPRS* penalized thin plate regression spline, *SPRINT* Systolic Blood Pressure Intervention Trial, *TTB* time to benefit, *WF* Weibull model with shared frailty, *WnoF* Weibull model without shared frailty

#### Results under Weibull baseline hazards (Scenario I)

Under Scenario I, all modeling approaches exhibited reduced bias and RMSE as sample size increased. Larger sample sizes were consistently associated with narrower confidence intervals across all methods. The empirical distributions of the estimates were approximately symmetric and unimodal in most settings. The parametric WFmodel yielded the lowest bias and RMSE, particularly for smaller ARR thresholds. In contrast, spline-based shared frailty models displayed greater variability in performance. The WnoF produced TTB estimates similar to those from the WF model, though with higher bias and RMSE, especially under lower censoring rates.

Confidence intervals constructed using the Monte Carlo method achieved coverage probabilities closer to the nominal level than those derived from the Delta method, particularly for spline-based models. Interval lengths decreased with increasing sample size, a pattern consistent across all approaches. Median point estimates from the Monte Carlo method were comparable to those obtained using the Delta method. Computation time varied substantially among methods: the WnoF model was the fastest, followed by the Weibull shared frailty model with Gamma frailty. Spline-based models, particularly PTPRS, required considerably longer computation times, especially under heavy censoring. Results under log-normal frailty were qualitatively similar to those under Gamma frailty. Gamma frailty models demonstrated superior computational efficiency due to their closed-form marginal survival functions. For the Weibull model with log-normal frailty, bimodal empirical distributions of the estimates were observed under certain conditions.

#### Results under piecewise Weibull hazards (Scenario II)

Under Scenario II, parametric Weibull models exhibited increased bias and reduced coverage probabilities compared to Scenario I. Differences among modeling approaches were more pronounced, with spline-based shared frailty models demonstrating smaller bias and improved coverage relative to the parametric Weibull model. PTPRS consistently achieved the lowest RMSE and coverage probabilities closest to the nominal level. Interval estimation based on MC simulation further enhanced coverage performance across all spline-based models. Under log-normal frailty, NCS showed larger bias and poorer coverage than other spline-based methods, whereas PTPRS maintained stable performance across settings. Patterns observed in Scenario I regarding sample size, censoring rate, and computational time remained consistent in this scenario.

## Real data analysis: the SPRINT trial

### Study population and data structure

We applied the proposed TTB framework to data from the SPRINT, a large multicenter randomized clinical trial whose design and objectives closely align with the methodological challenges outlined in the Introduction. SPRINT was designed not only to assess whether intensive systolic blood pressure (SBP) control reduces major cardiovascular events but also to characterize the temporal pattern over which such benefits emerge, making it a highly relevant real-world setting for evaluating TTB estimation.

**Fig. 1:**
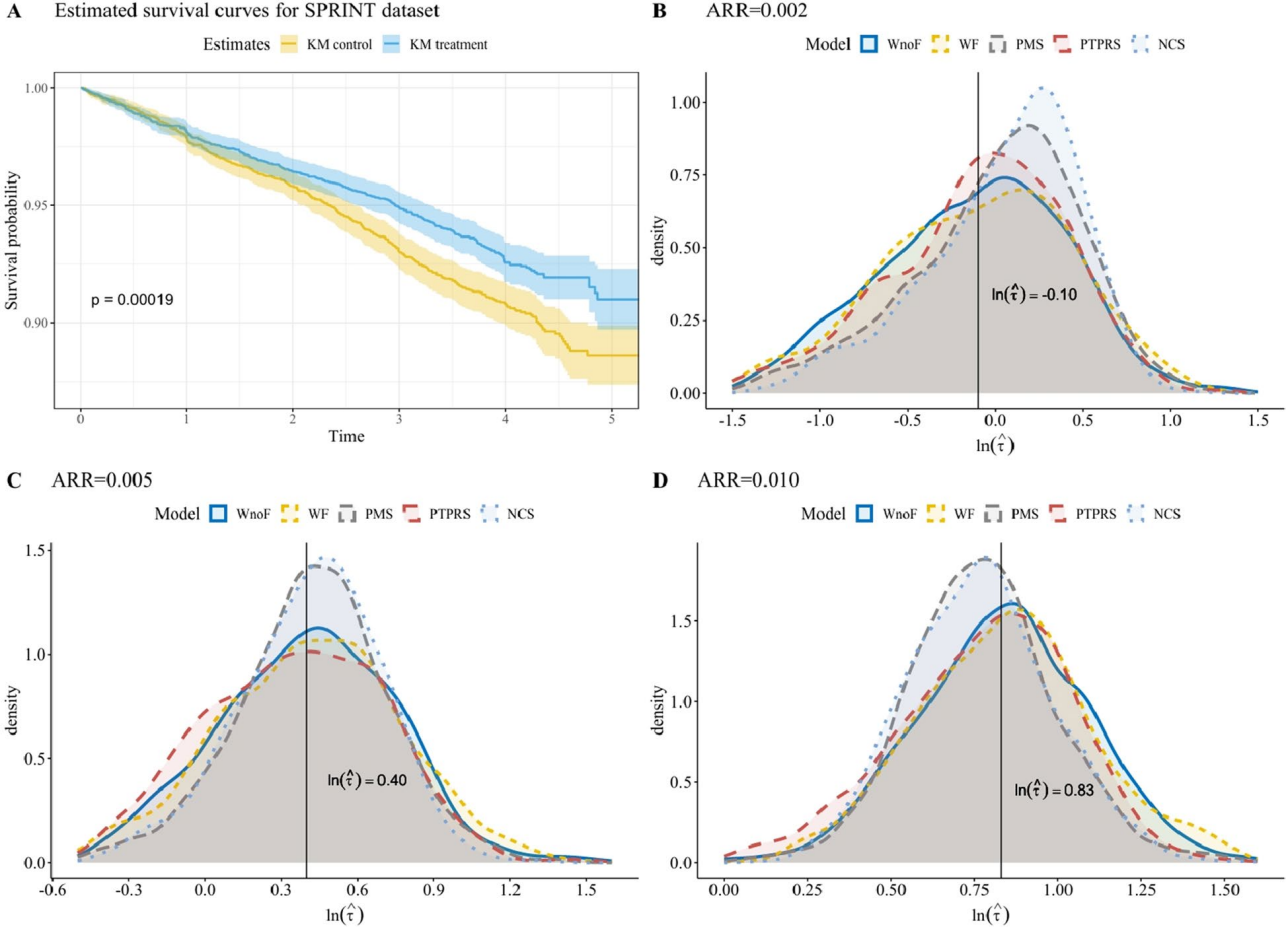
Real data analysis results for the SPRINT dataset. Panel **A** shows the estimated KM survival curves with 95% CIs. Panels **B**–**D** display the empirical sampling distributions of the logarithms of TTB estimates from four models incorporating Gamma frailty, based on 1,000 MC samples. Vertical lines indicate the estimated logarithms of TTB derived from the Weibull model using the Delta method. ARR: absolute relative risk; CI: confidence interval; KM: Kaplan–Meier; MC: Monte Carlo; NCS: natural cubic spline; PMS: penalized M-spline; PTPRS: penalized thin plate regression spline; SPRINT: Systolic Blood Pressure Intervention Trial; TTB: time to benefit; WF: Weibull model with shared frailty; WnoF: Weibull model without shared frailty

The trial enrolled 9,361 participants at elevated cardiovascular risk without diabetes across 102 clinical sites, randomly assigning them to an intensive-treatment group targeting SBP < 120 mmHg or a standard-treatment group targeting SBP < 140 mmHg. The primary outcome was a composite of major cardiovascular events, with a median follow-up of 3.792 years. Censoring rates were low, at 6.7% in the control group and 8.8% in the treatment group. Baseline characteristics of the study population are summarized in Table 5, and Kaplan–Meier survival curves for the two treatment groups are presented in Fig. 1A.

**Table 5:** Characteristics of the SPRINT dataset

Variable	Level	Control	Treatment	*P*
*N*	–––-	4683	4678	
Time, years, Median [IQR]	–––-	3.781 [3.265, 4.307]	3.806 [3.288, 4.370]	0.018
Status (%)	Censored	4272 (91.2)	4363 (93.3)	< 0.001
	Death	411 (8.8)	315 (6.7)	

The *p* values are calculated by the Wilcoxon-Mann–Whitney test and the Chi-squared test for the Time and Status variables, respectively

*SPRINT* systolic blood pressure intervention trial

Beyond these design features, SPRINT exhibits a clustered survival structure typical of modern large-scale trials, with participants nested within clinical sites that vary in baseline cardiovascular risk and event dynamics. This between-site heterogeneity may influence marginal survival functions and, consequently, the estimated timing at which clinically meaningful benefit thresholds are crossed. In this context, SPRINT provides a particularly appropriate example for evaluating how unmeasured heterogeneity and complex hazard shapes affect the estimation and interpretation of TTB in practice.

### Model implementation

The modeling framework described in Sect. “Method” was applied to the SPRINT data, treating clinical site as the clustering unit to account for potential site-level variation in baseline event risk. Analyses were conducted under both Gamma and log-normal frailty specifications, reflecting alternative assumptions about the distribution of unobserved heterogeneity across sites. For each frailty distribution, five baseline hazard models were fitted: a parametric WnoF, a parametric WF, and three spline-based shared frailty models using PMS, PTPRS, and NCS.

Baseline hazard functions were estimated separately for the intensive and standard treatment groups to allow for time-varying treatment effects beyond the proportional hazards assumption. Model fitting was performed using maximum likelihood or penalized maximum likelihood estimation, depending on the hazard specification. For spline-based models, smoothing parameters were selected using default data-driven criteria implemented in the corresponding software. Marginal survival functions for each treatment group were obtained by integrating over the frailty distribution, yielding population-averaged survival estimates consistent with the marginal definition of TTB adopted in Sect. “Baseline hazard specification”.

### Estimation of TTB

TTB was defined as the earliest time point at which the absolute risk reduction between the treatment and control groups exceeded a prespecified threshold *Δ*. Analyses were conducted for thresholds of 0.005 and 0.010, corresponding to clinically interpretable risk differences over time. Point estimates of TTB were derived numerically from the estimated marginal survival functions. Confidence intervals were constructed using both the Delta method and the Monte Carlo (MC) procedure described in Sect. “Statistical inference for TTB as a derived quantity”. For this application, 1,000 Monte Carlo resamples were used, a number found to be sufficient based on the operating characteristics observed in the simulation study. Empirical distributions of TTB estimates from the MC procedure are displayed in Fig. 1B–D and Supplementary Figure S9 for selected models under both frailty distributions.

### Results

Estimated TTB values and corresponding 95% confidence intervals are reported in Table 6. Across modeling approaches, point estimates of TTB were broadly similar, yielding approximate values of 1.0, 1.5, and 2.2 years for ARR thresholds of 0.005, 0.0075, and 0.010, respectively. This apparent stability in point estimation reflects the relatively regular hazard patterns and moderate censoring observed in the SPRINT trial. However, such consistency should not be interpreted as evidence that modeling choices are inconsequential for TTB estimation in general.

**Table 6:** Time to benefit estimation results for SPRINT dataset

			Gamma frailty		Log-normal frailty	
ARR	Model	Method	Estimate (95% CI)	Time(s)	Estimate (95% CI)	Time(s)
0.002	WnoF	Delta	0.908 (0.294, 2.802)	1	0.908 (0.294, 2.802)	1
		MC	0.931 (0.279, 2.240)	1	0.931 (0.279, 2.240)	1
	WF	Delta	0.907 (0.292, 2.815)	3	0.906 (0.293, 2.809)	19
		MC	0.927 (0.273, 2.343)	5	0.925 (0.272, 2.334)	24
	PMS	Delta	1.089 (0.425, 2.791)	1	1.094 (0.430, 2.784)	34
		MC	1.070 (0.311, 2.225)	7	1.074 (0.310, 2.222)	45
	PTPRS	Delta	0.928 (0.353, 2.435)	96	0.928 (0.353, 2.436)	388
		MC	0.924 (0.134, 2.145)	1259	0.923 (0.133, 2.139)	1626
	NCS	Delta	1.129 (0.496, 2.572)	8	1.129 (0.496, 2.572)	13
		MC	1.130 (0.377, 2.038)	496	1.130 (0.376, 2.036)	533
0.005	WnoF	Delta	1.498 (0.739, 3.038)	–––-	1.498 (0.739, 3.038)	–––-
		MC	1.504 (0.708, 2.851)	–––-	1.504 (0.708, 2.851)	–––-
	WF	Delta	1.489 (0.731, 3.036)	–––-	1.487 (0.730, 3.032)	–––-
		MC	1.519 (0.687, 3.072)	–––-	1.511 (0.682, 3.072)	–––-
	PMS	Delta	1.553 (0.870, 2.773)	–––-	1.555 (0.873, 2.769)	–––-
		MC	1.532 (0.774, 2.714)	–––-	1.532 (0.775, 2.732)	–––-
	PTPRS	Delta	1.430 (0.665, 3.079)	–––-	1.430 (0.664, 3.078)	–––-
		MC	1.432 (0.679, 2.662)	–––-	1.430 (0.676, 2.649)	–––-
	NCS	Delta	1.538 (0.905, 2.614)	–––-	1.537 (0.904, 2.614)	–––-
		MC	1.548 (0.807, 2.482)	–––-	1.545 (0.797, 2.482)	–––-
0.010	WnoF	Delta	2.313 (1.411, 3.791)	–––-	2.313 (1.411, 3.791)	–––-
		MC	2.327 (1.398, 3.867)	–––-	2.327 (1.398, 3.867)	–––-
	WF	Delta	2.294 (1.393, 3.778)	–––-	2.290 (1.390, 3.773)	–––-
		MC	2.339 (1.352, 4.130)	–––-	2.325 (1.349, 4.103)	–––-
	PMS	Delta	2.172 (1.406, 3.354)	–––-	2.170 (1.408, 3.346)	–––-
		MC	2.164 (1.432, 3.476)	–––-	2.153 (1.425, 3.419)	–––-
	PTPRS	Delta	2.238 (1.361, 3.682)	–––-	2.237 (1.359, 3.680)	–––-
		MC	2.239 (1.229, 3.476)	–––-	2.234 (1.229, 3.475)	–––-
	NCS	Delta	2.128 (1.373, 3.297)	–––-	2.126 (1.372, 3.296)	–––-
		MC	2.149 (1.421, 3.394)	–––-	2.147 (1.413, 3.399)	–––-

The number of MC samples is 1,000. Time column is the computation time for all four models

*ARR* absolute relative risk, *MC* Monte Carlo, *NCS* natural cubic spline, *PMS* penalized M-spline, *PTPRS* penalized thin plate regression spline, *SPRINT* Systolic Blood Pressure Intervention Trial, *TTB* time to benefit, *WnoF* Weibull model without failty, *WF* Weibull shared frailty model

Although point estimates were similar, shared frailty models yielded statistically significant frailty variance estimates under both Gamma and log-normal specifications (all *p* < 0.010), indicating substantial between-site heterogeneity in baseline cardiovascular risk. Notably, while this heterogeneity did not substantially shift the estimated timing of benefit onset in this dataset, it markedly affected uncertainty quantification. Models that ignored frailty produced narrower confidence intervals, whereas shared frailty models yielded wider intervals that better accounted for uncertainty arising from latent heterogeneity. Given that TTB is defined as a threshold-crossing estimand at the marginal population level, these differences in interval width have direct implications for clinical interpretation, particularly when treatment decisions depend on whether anticipated benefits are likely to be realized within a specific time horizon.

Baseline hazard specification further influenced the inferential behavior of TTB, primarily through its impact on the smoothness and stability of estimated marginal survival functions. Flexible spline-based shared frailty models produced smoother empirical distributions of TTB under MC-based inference, whereas parametric Weibull models occasionally exhibited irregular and sometimes multimodal behavior, particularly under log-normal frailty. These patterns mirror those observed in the simulation study and underscore that hazard flexibility plays a substantive role in stabilizing inference for implicitly defined estimands, even when point estimates remain relatively unchanged.

Computational considerations also differed across modeling approaches. The Weibull model without frailty completed estimation in approximately one second, whereas spline-based shared frailty models, especially those combined with log-normal frailty, required substantially longer computation times. These differences illustrate the trade-offs between computational efficiency and inferential robustness across modeling strategies in the SPRINT application, particularly in the presence of heterogeneity and complex hazard dynamics.

## Discussion

TTB has garnered increasing attention as a clinically interpretable estimand for characterizing not only whether a treatment is effective, but also when its benefits are likely to emerge [18–20]. Unlike conventional survival summaries evaluated at fixed time points, TTB is implicitly defined through marginal survival differences over time and is therefore intrinsically sensitive to modeling assumptions. This work formalizes that sensitivity and demonstrates that, in clustered survival settings, coherent estimation of TTB requires explicit alignment among the estimand definition, the survival model used for marginalization, and the inferential strategy employed to quantify uncertainty.

A central contribution of the proposed framework lies in its treatment of unobserved cluster-level heterogeneity. Shared frailty models are routinely used to account for within-cluster dependence in time-to-event data, yet their implications for threshold-based estimands such as TTB have received limited attention. By defining TTB on the marginal population scale and integrating over the frailty distribution, the framework targets clinically interpretable population-level quantities. Simulations and the SPRINT application show that ignoring latent heterogeneity can distort TTB inference, especially when the ARR threshold is small or when threshold-crossing behavior is sensitive to marginal survival estimation. These findings underscore that frailty modeling in TTB analysis is not merely a technical adjustment for correlation; it directly shapes the marginal survival contrast from which TTB is derived.

Baseline hazard specification is a second key determinant of TTB estimation. Parametric Weibull models offer computational simplicity but may be restrictive when hazards exhibit complex patterns. When the hazard departs from the Weibull form, semiparametric spline-based hazards can improve flexibility and stabilize marginal survival estimation. Among the spline approaches considered, PTPRS typically provides the most accurate point and interval estimates, PMS offers a pragmatic compromise, and NCS is fastest but may be less reliable due to tuning sensitivity. In practice, model choice involves balancing flexibility against computational cost.

Overall, the main runtime drivers were flexible spline baselines (especially PTPRS), MC-based intervals, and log-normal frailty due to numerical integration. As a practical and scalable default, we recommend to start with a Weibull baseline with Gamma shared frailty, assess hazard shape and model fit, and then escalate to a more flexible baseline when warranted (PMS when PTPRS is prohibitive and NCS with caution), and using MC intervals and/or log-normal frailty primarily as sensitivity analyses.

Across frailty specifications, Gamma and log-normal frailty often yield similar point estimates in our experiments, but Gamma is generally more computationally efficient and numerically stable because it avoids the numerical integration required by log-normal frailty; the associated approximation error can grow with frailty variance [5, 11]. Accordingly, Gamma shared frailty is a sensible default, with log-normal frailty serving as a useful sensitivity analysis when heavier-tailed heterogeneity is plausible.

Uncertainty quantification is challenging because TTB is defined implicitly as a threshold-crossing time and can be non-smooth. We therefore considered both Delta method and MC-based intervals. The Delta method is an efficient default when asymptotic normality is credible, whereas MC can improve coverage when ARR is small or when normal approximations for the induced TTB distribution are doubtful, at added computational cost. Importantly, the MC procedure does not eliminate reliance on asymptotic normality altogether: it typically samples parameters from an estimated asymptotic multivariate normal distribution for regression parameter estimates​, thereby relying on first-level asymptotic normality, while sidestepping the Delta method’s additional linearization of the nonlinear TTB functional.

A more robust alternative, albeit often more computationally intensive, is the nonparametric cluster-level bootstrap, which resamples clusters from the observed data and can reduce reliance on parametric and asymptotic approximations. However, for flexible hazard shared frailty models, particularly those with log-normal frailty, bootstrap refitting can be prohibitively expensive in realistic multicenter datasets. We therefore view the bootstrap primarily as a sensitivity analysis option when computational resources permit, rather than as a default.

Several limitations qualify these recommendations. Numerical instability is more likely with log-normal frailty and very small ARR, where the survival-difference function can flatten near the TTB threshold. The assumption of conditional independence within centers given frailty may be violated when additional clustering exists (e.g., physicians within centers), and reliable estimation of frailty variance requires sufficient between-center variation.

Future work should prioritize scalability and broader applicability. Time-splitting approaches such as the Piecewise Exponential Additive Mixed Model (PAMM) [21] offer a flexible frequentist route for complex hazards with random effects, though scaling can be memory and computation intensive. Within a Bayesian formulation, the Integrated Nested Laplace Approximation (INLA) [22] may provide a faster alternative to Markov chain Monte Carlo (MCMC) for the posterior quantities required to compute TTB and its uncertainty. More broadly, extensions to time-varying effects, competing risks, and multi-state processes can yield cause- or state-specific TTB [13]; joint models with longitudinal biomarkers may enable dynamic TTB [23]; and integration with causal survival estimators may address confounding and clustering simultaneously [24–26]. Alternative frailty distributions (e.g., positive stable, power-variance families [7, 11]) merit evaluation, as do design tools for power and sample size when TTB is a primary endpoint, and extensions to interval censoring and left truncation for registry settings. Evidence synthesis is another direction: one-stage hierarchical shared-frailty models can pool log TTB using individual participant data, whereas two-stage random-effects meta-analysis can be applied to study-level log TTB estimates with appropriate small-sample adjustments [27–29].

Bayesian approaches can be attractive for TTB because they provide coherent uncertainty propagation and allow prior regularization, which may help stabilize frailty variance estimation in small-center settings. However, Bayesian analyses can be sensitive to prior choices and may be computationally demanding for frailty and flexible hazard models; generic MCMC can be slow and requires careful convergence diagnostics. Under weakly informative priors and regular models, posterior modes or means may align closely with maximum likelihood estimation (MLE)-based inference, but this is not guaranteed and should be checked in practice [10].

Finally, interpretation and use should be cautious. TTB is probabilistic and should be reported with an appropriate uncertainty interval to avoid overconfident clinical decisions, particularly when center-level heterogeneity is substantial. Frailty is a device for modeling unobserved heterogeneity rather than a measure of provider quality and should not be interpreted as such [7, 11]. When communicated appropriately, the framework can support multicenter decision-making by clarifying how heterogeneity shifts the time horizon over which benefits are likely to emerge [19, 30].

## Conclusion

We propose an estimand-based framework for estimating TTB in clustered survival settings by aligning the marginal estimand definition, shared frailty modeling, baseline hazard specification, and uncertainty quantification. Simulations and a multicenter application demonstrate how assumptions regarding frailty distribution, flexibility of the hazard function, and methods for interval construction jointly influence both TTB estimates and their associated uncertainty. In practice, we recommend beginning with a Weibull baseline hazard with Gamma distributed shared frailty, using Delta method-based confidence intervals, then increasing hazard flexibility and/or adopting MC-based intervals as diagnostics assessments warrant. Uncertainty should always be reported to reflect the inherently probabilistic nature of TTB. This framework facilitates transparent and clinically interpretable TTB evaluation in multicenter trials and real-world survival analyses.

## Supplementary Information

Supplementary Material 1.

## Data Availability

The data that support the findings of this study are not openly available due to reasons of sensitivity and are available from the corresponding author upon reasonable request.

## Abbreviations

ARR: Absolute risk reduction
CP: Coverage probability
INLA: Integrated nested Laplace approximation
MAE: Mean absolute error
MC: Monte Carlo
MCMC: Markov Chain Monte Carlo
MLE: Maximum likelihood estimation
NCS: Natural cubic splines
OLLP: Out-of-lower-limit probability
OULP: Out-of-upper-limit probability
PMS: Penalized M-splines
PTPRS: Penalized thin-plate regression splines
RMSE: Root mean squared error
SBP: Systolic blood pressure
SPRINT: Pressure Intervention Trial
TTB: Time to benefit
WF: Weibull shared frailty model
WnoF: Weibull model without frailty
