# Supplementary material for: Time to benefit estimation in multicenter studies using flexible hazard shared frailty models
Source: BMC Med Res Methodol. 2026 Mar 7;26:84. doi: 10.1186/s12874-026-02816-1 (PMC13081241; doi:10.1186/s12874-026-02816-1)
Supplement: Supplementary file 1 — Supplementary Material 1. [file 12874_2026_2816_MOESM1_ESM.docx]

# Supplementary Material

## Supplemenraty Tables

**Table S1.** Censoring Weibull distribution parameters for simulations.

|  |  | Gamma frailty  Weibull parameters | | Log-normal frailty  Weibull parameters | |
| --- | --- | --- | --- | --- | --- |
| Scenario | Censoring rate | Shape | Scale | Shape | Scale |
| I | 30% | 1.130 | 94.428 | 0.939 | 110.647 |
|  | 60% | 1.348 | 27.797 | 1.328 | 28.480 |
|  | 90% | 1.692 | 5.431 | 1.676 | 5.585 |
| II | 30% | 0.614 | 176.464 | 0.880 | 117.182 |
|  | 60% | 1.313 | 27.812 | 1.321 | 28.435 |
|  | 90% | 1.371 | 5.284 | 1.710 | 5.546 |

**Table S2.** TTB point estimation results for simulation scenario I with the Gamma frailty and 1,000 replicates.

|  |  |  |  | N=5,000 | | | | N=10,000 | | | |
| --- | --- | --- | --- | --- | --- | --- | --- | --- | --- | --- | --- |
| Cesnoring | Model | Method | ARR | Bias | MAE | RMSE | Time | Bias | MAE | RMSE | Time |
| 0% | WnoF | Delta | 0.002 | -0.053 | 0.372 | 0.460 | 1min | -0.039 | 0.262 | 0.325 | 1min |
|  |  | MC |  | -0.064 | 0.372 | 0.459 | 30min | -0.047 | 0.263 | 0.325 | 1hrs30min |
|  | WF | Delta |  | -0.001 | 0.339 | 0.417 | 26min | 0.004 | 0.239 | 0.296 | 1hrs52min |
|  |  | MC |  | -0.023 | 0.339 | 0.417 | 2hrs55min | -0.012 | 0.239 | 0.295 | 2hrs21min |
|  | PMS | Delta |  | -0.107 | 0.631 | 0.772 | 3hrs30min | -0.092 | 0.482 | 0.593 | 8hrs16min |
|  |  | MC |  | -0.063 | 0.610 | 0.748 | 6hrs37min | -0.057 | 0.467 | 0.575 | 10hrs24min |
|  | PTPRS | Delta |  | -0.048 | 0.482 | 0.602 | 13hrs43min | -0.046 | 0.360 | 0.454 | 15hrs49min |
|  | NCS | Delta |  | -0.132 | 0.514 | 0.620 | 4hrs50min | -0.148 | 0.396 | 0.479 | 4hrs4min |
|  | WnoF | Delta | 0.005 | -0.043 | 0.272 | 0.337 |  | -0.034 | 0.191 | 0.237 |  |
|  |  | MC |  | -0.050 | 0.273 | 0.338 |  | -0.040 | 0.191 | 0.238 |  |
|  | WF | Delta |  | 0.001 | 0.247 | 0.306 |  | 0.004 | 0.174 | 0.215 |  |
|  |  | MC |  | -0.015 | 0.248 | 0.306 |  | -0.008 | 0.175 | 0.215 |  |
|  | PMS | Delta |  | -0.025 | 0.405 | 0.509 |  | -0.012 | 0.287 | 0.362 |  |
|  |  | MC |  | 0.001 | 0.394 | 0.496 |  | 0.005 | 0.280 | 0.353 |  |
|  | PTPRS | Delta |  | -0.022 | 0.327 | 0.411 |  | -0.021 | 0.237 | 0.300 |  |
|  | NCS | Delta |  | -0.051 | 0.364 | 0.446 |  | -0.052 | 0.265 | 0.328 |  |
|  | WnoF | Delta | 0.010 | -0.031 | 0.210 | 0.261 |  | -0.024 | 0.147 | 0.182 |  |
|  |  | MC |  | -0.034 | 0.210 | 0.262 |  | -0.027 | 0.147 | 0.183 |  |
|  | WF | Delta |  | 0.001 | 0.193 | 0.239 |  | 0.003 | 0.135 | 0.167 |  |
|  |  | MC |  | -0.012 | 0.193 | 0.239 |  | -0.007 | 0.136 | 0.168 |  |
|  | PMS | Delta |  | -0.002 | 0.278 | 0.353 |  | 0.007 | 0.189 | 0.240 |  |
|  |  | MC |  | 0.012 | 0.276 | 0.349 |  | 0.015 | 0.188 | 0.239 |  |
|  | PTPRS | Delta |  | -0.013 | 0.238 | 0.300 |  | -0.010 | 0.170 | 0.215 |  |
|  | NCS | Delta |  | -0.020 | 0.273 | 0.338 |  | -0.014 | 0.192 | 0.241 |  |
| 30% | WnoF | Delta | 0.002 | -0.172 | 0.429 | 0.524 | 1min | -0.171 | 0.318 | 0.393 | 1min |
|  |  | MC |  | -0.187 | 0.433 | 0.528 | 1hrs30min | -0.182 | 0.322 | 0.398 | 1hrs31min |
|  | WF | Delta |  | -0.008 | 0.381 | 0.468 | 27min | -0.008 | 0.267 | 0.334 | 1hrs41min |
|  |  | MC |  | -0.028 | 0.382 | 0.468 | 2hrs58min | -0.021 | 0.267 | 0.334 | 2hrs15min |
|  | PMS | Delta |  | -0.100 | 0.631 | 0.770 | 2hrs41min | -0.068 | 0.459 | 0.569 | 4hrs52min |
|  |  | MC |  | -0.064 | 0.609 | 0.746 | 4hrs10min | -0.038 | 0.444 | 0.552 | 6hrs21min |
|  | PTPRS | Delta |  | -0.054 | 0.498 | 0.622 | 14hrs1min | -0.048 | 0.367 | 0.465 | 16hrs22min |
|  | NCS | Delta |  | -0.086 | 0.545 | 0.652 | 4hrs12min | -0.116 | 0.423 | 0.512 | 4hrs29min |
|  | WnoF | Delta | 0.005 | -0.118 | 0.308 | 0.378 |  | -0.118 | 0.226 | 0.280 |  |
|  |  | MC |  | -0.129 | 0.311 | 0.381 |  | -0.125 | 0.229 | 0.283 |  |
|  | WF | Delta |  | -0.003 | 0.276 | 0.341 |  | -0.004 | 0.192 | 0.241 |  |
|  |  | MC |  | -0.017 | 0.277 | 0.341 |  | -0.014 | 0.192 | 0.240 |  |
|  | PMS | Delta |  | -0.024 | 0.408 | 0.509 |  | -0.010 | 0.280 | 0.354 |  |
|  |  | MC |  | -0.002 | 0.402 | 0.500 |  | 0.006 | 0.277 | 0.349 |  |
|  | PTPRS | Delta |  | -0.023 | 0.337 | 0.423 |  | -0.021 | 0.242 | 0.306 |  |
|  | NCS | Delta |  | -0.028 | 0.380 | 0.465 |  | -0.038 | 0.281 | 0.346 |  |
|  | WnoF | Delta | 0.010 | -0.084 | 0.235 | 0.289 |  | -0.082 | 0.170 | 0.211 |  |
|  |  | MC |  | -0.091 | 0.236 | 0.291 |  | -0.088 | 0.171 | 0.213 |  |
|  | WF | Delta |  | -0.003 | 0.213 | 0.264 |  | -0.003 | 0.148 | 0.185 |  |
|  |  | MC |  | -0.013 | 0.213 | 0.264 |  | -0.011 | 0.148 | 0.185 |  |
|  | PMS | Delta |  | -0.004 | 0.280 | 0.353 |  | -0.002 | 0.192 | 0.242 |  |
|  |  | MC |  | 0.011 | 0.279 | 0.350 |  | 0.009 | 0.193 | 0.242 |  |
|  | PTPRS | Delta |  | -0.012 | 0.243 | 0.307 |  | -0.011 | 0.174 | 0.219 |  |
|  | NCS | MC |  | -0.010 | 0.278 | 0.345 |  | -0.009 | 0.198 | 0.248 |  |
| 60% | WnoF | Delta | 0.002 | -0.112 | 0.463 | 0.565 | 1min | -0.113 | 0.329 | 0.409 | 1min |
|  |  | Delta |  | -0.121 | 0.462 | 0.564 | 1hrs30min | -0.120 | 0.329 | 0.409 | 1hrs31min |
|  | WF | Delta |  | -0.013 | 0.441 | 0.541 | 23min | -0.015 | 0.308 | 0.383 | 1hrs35min |
|  |  | MC |  | -0.042 | 0.443 | 0.543 | 2hrs57min | -0.037 | 0.308 | 0.383 | 2hrs11min |
|  | PMS | Delta |  | -0.108 | 0.638 | 0.774 | 1hrs58min | -0.067 | 0.463 | 0.573 | 2hrs5min |
|  |  | MC |  | -0.072 | 0.627 | 0.759 | 4hrs14min | -0.039 | 0.458 | 0.565 | 5hrs45min |
|  | PTPRS | Delta |  | -0.051 | 0.509 | 0.635 | 17hrs3min | -0.052 | 0.380 | 0.481 | 16hrs17min |
|  | NCS | MC |  | -0.068 | 0.584 | 0.704 | 4hrs6min | -0.083 | 0.451 | 0.547 | 4hrs20min |
|  | WnoF | Delta | 0.005 | -0.070 | 0.328 | 0.404 |  | -0.071 | 0.231 | 0.287 |  |
|  |  | Delta |  | -0.076 | 0.327 | 0.403 |  | -0.077 | 0.230 | 0.287 |  |
|  | WF | Delta |  | -0.005 | 0.315 | 0.390 |  | -0.008 | 0.217 | 0.271 |  |
|  |  | MC |  | -0.026 | 0.316 | 0.390 |  | -0.023 | 0.218 | 0.272 |  |
|  | PMS | Delta |  | -0.027 | 0.413 | 0.517 |  | -0.012 | 0.286 | 0.361 |  |
|  |  | MC |  | -0.003 | 0.407 | 0.508 |  | 0.006 | 0.284 | 0.356 |  |
|  | PTPRS | Delta |  | -0.019 | 0.344 | 0.432 |  | -0.022 | 0.249 | 0.314 |  |
|  | NCS | MC |  | -0.030 | 0.401 | 0.499 |  | -0.029 | 0.289 | 0.360 |  |
|  | WnoF | Delta | 0.010 | -0.045 | 0.247 | 0.306 |  | -0.046 | 0.172 | 0.215 |  |
|  |  | Delta |  | -0.051 | 0.247 | 0.306 |  | -0.050 | 0.172 | 0.215 |  |
|  | WF | Delta |  | -0.004 | 0.239 | 0.298 |  | -0.006 | 0.164 | 0.205 |  |
|  |  | MC |  | -0.019 | 0.239 | 0.298 |  | -0.016 | 0.165 | 0.206 |  |
|  | PMS | Delta |  | -0.007 | 0.286 | 0.362 |  | -0.001 | 0.195 | 0.246 |  |
|  |  | MC |  | 0.010 | 0.281 | 0.357 |  | 0.011 | 0.193 | 0.243 |  |
|  | PTPRS | Delta |  | -0.009 | 0.250 | 0.315 |  | -0.012 | 0.178 | 0.224 |  |
|  | NCS | MC |  | -0.014 | 0.286 | 0.363 |  | -0.009 | 0.196 | 0.248 |  |
| 90% | WnoF | Delta | 0.002 | -0.064 | 0.542 | 0.676 | 1min | -0.046 | 0.397 | 0.494 | 1min |
|  |  | Delta |  | -0.058 | 0.536 | 0.669 | 1hrs38min | -0.041 | 0.394 | 0.491 | 1hrs34min |
|  | WF | Delta |  | -0.055 | 0.537 | 0.670 | 19min | -0.040 | 0.394 | 0.491 | 1hrs33min |
|  |  | MC |  | -0.085 | 0.547 | 0.681 | 3hrs42min | -0.047 | 0.398 | 0.495 | 2hrs15min |
|  | PMS | Delta |  | -0.123 | 0.650 | 0.796 | 1hrs31min | -0.057 | 0.467 | 0.585 | 1hrs9min |
|  |  | MC |  | -0.083 | 0.638 | 0.785 | 15hrs10min | -0.027 | 0.452 | 0.569 | 15hrs37min |
|  | PTPRS | Delta |  | -0.070 | 0.557 | 0.700 | 24hrs18min | -0.046 | 0.411 | 0.518 | 40hrs21min |
|  | NCS | MC |  | -0.124 | 0.637 | 0.794 | 4hrs29min | -0.063 | 0.466 | 0.596 | 5hrs36min |
|  | WnoF | Delta | 0.005 | -0.029 | 0.374 | 0.471 |  | -0.019 | 0.265 | 0.334 |  |
|  |  | Delta |  | -0.030 | 0.366 | 0.463 |  | -0.018 | 0.262 | 0.330 |  |
|  | WF | Delta |  | -0.027 | 0.372 | 0.469 |  | -0.017 | 0.264 | 0.332 |  |
|  |  | MC |  | -0.044 | 0.374 | 0.472 |  | -0.021 | 0.263 | 0.332 |  |
|  | PMS | Delta |  | -0.027 | 0.420 | 0.527 |  | -0.006 | 0.293 | 0.373 |  |
|  |  | MC |  | -0.003 | 0.416 | 0.524 |  | 0.006 | 0.292 | 0.371 |  |
|  | PTPRS | Delta |  | -0.027 | 0.379 | 0.477 |  | -0.017 | 0.271 | 0.342 |  |
|  | NCS | MC |  | -0.050 | 0.427 | 0.536 |  | -0.029 | 0.308 | 0.390 |  |
|  | WnoF | Delta | 0.010 | -0.010 | 0.278 | 0.353 |  | -0.004 | 0.192 | 0.244 |  |
|  |  | Delta |  | -0.019 | 0.273 | 0.345 |  | -0.008 | 0.190 | 0.242 |  |
|  | WF | Delta |  | -0.011 | 0.278 | 0.352 |  | -0.005 | 0.192 | 0.243 |  |
|  |  | MC |  | -0.023 | 0.277 | 0.350 |  | -0.011 | 0.192 | 0.243 |  |
|  | PMS | Delta |  | 0.000 | 0.294 | 0.371 |  | 0.003 | 0.211 | 0.266 |  |
|  |  | MC |  | 0.008 | 0.290 | 0.367 |  | 0.011 | 0.209 | 0.263 |  |
|  | PTPRS | Delta |  | -0.009 | 0.281 | 0.356 |  | -0.006 | 0.196 | 0.248 |  |
|  | NCS | MC |  | -0.026 | 0.312 | 0.390 |  | -0.017 | 0.227 | 0.284 |  |

Note: Metrics are calculated based on the logarithms of TTB estimates. The number of MC samples is 2,000. For MC method, the estimates are the median of the MC samples. Time is for all ARR values for each Model. ARR: absolute relative risk; MAE: mean absolute error; RMSE: root mean squared error; MC: Monte Carlo; NCS: natural cubic spline; PMS: penalized M-spline; PTPRS: penalized thin plate regression spline; SPRINT: Systolic Blood Pressure Intervention Trial; TTB: time to benefit; WF: Weibull model with shared frailty; WnoF: Weibull model without shared frailty

**Table S3.** TTB interval estimation results for simulation scenario I with the Gamma frailty and 1,000 replicates.

|  |  |  |  | N=5,000 | | | | N=10,000 | | | |
| --- | --- | --- | --- | --- | --- | --- | --- | --- | --- | --- | --- |
| Cesnoring | Model | Method | ARR | CP | OLLP | OULP | length | CP | OLLP | OULP | length |
| 0% | WnoF | Delta | 0.002 | 0.924 | 0.028 | 0.048 | 1.788 | 0.941 | 0.023 | 0.036 | 1.287 |
|  |  | MC |  | 0.944 | 0.024 | 0.032 | 1.731 | 0.954 | 0.019 | 0.027 | 1.267 |
|  | WF | Delta |  | 0.929 | 0.035 | 0.035 | 1.633 | 0.946 | 0.027 | 0.028 | 1.175 |
|  |  | MC |  | 0.947 | 0.023 | 0.029 | 1.581 | 0.953 | 0.020 | 0.027 | 1.151 |
|  | PMS | Delta |  | 0.812 | 0.098 | 0.089 | 3.006 | 0.865 | 0.079 | 0.056 | 2.304 |
|  |  | MC |  | 0.933 | 0.034 | 0.033 | 2.536 | 0.946 | 0.030 | 0.023 | 2.025 |
|  | PTPRS | Delta |  | 0.911 | 0.055 | 0.034 | 2.534 | 0.939 | 0.039 | 0.022 | 1.928 |
|  | NCS | Delta |  | 0.843 | 0.045 | 0.112 | 2.388 | 0.880 | 0.027 | 0.093 | 1.853 |
|  | WnoF | Delta | 0.005 | 0.931 | 0.023 | 0.046 | 1.311 | 0.946 | 0.018 | 0.036 | 0.937 |
|  |  | MC |  | 0.952 | 0.022 | 0.026 | 1.297 | 0.955 | 0.018 | 0.027 | 0.939 |
|  | Weibull | Delta |  | 0.939 | 0.027 | 0.033 | 1.197 | 0.949 | 0.026 | 0.026 | 0.856 |
|  |  | MC |  | 0.950 | 0.022 | 0.027 | 1.176 | 0.954 | 0.020 | 0.026 | 0.843 |
|  | PMS | Delta |  | 0.874 | 0.074 | 0.051 | 1.959 | 0.913 | 0.062 | 0.025 | 1.445 |
|  |  | MC |  | 0.934 | 0.038 | 0.028 | 1.813 | 0.943 | 0.036 | 0.020 | 1.364 |
|  | PTPRS | Delta |  | 0.933 | 0.045 | 0.022 | 1.696 | 0.946 | 0.032 | 0.022 | 1.266 |
|  | NCS | Delta |  | 0.883 | 0.044 | 0.073 | 1.751 | 0.920 | 0.029 | 0.051 | 1.320 |
|  | WnoF | Delta | 0.010 | 0.936 | 0.023 | 0.041 | 1.019 | 0.945 | 0.020 | 0.035 | 0.725 |
|  |  | MC |  | 0.955 | 0.020 | 0.025 | 1.014 | 0.955 | 0.020 | 0.025 | 0.728 |
|  | Weibull | Delta |  | 0.941 | 0.029 | 0.029 | 0.934 | 0.952 | 0.024 | 0.025 | 0.666 |
|  |  | MC |  | 0.956 | 0.018 | 0.025 | 0.928 | 0.952 | 0.023 | 0.026 | 0.661 |
|  | PMS | Delta |  | 0.910 | 0.057 | 0.033 | 1.353 | 0.942 | 0.042 | 0.016 | 0.971 |
|  |  | MC |  | 0.938 | 0.035 | 0.027 | 1.329 | 0.954 | 0.028 | 0.017 | 0.958 |
|  | PTPRS | Delta |  | 0.940 | 0.035 | 0.025 | 1.225 | 0.959 | 0.021 | 0.020 | 0.904 |
|  | NCS | Delta |  | 0.900 | 0.049 | 0.051 | 1.327 | 0.936 | 0.033 | 0.031 | 0.978 |
| 30% | WnoF | Delta | 0.002 | 0.906 | 0.018 | 0.076 | 1.918 | 0.906 | 0.009 | 0.085 | 1.391 |
|  |  | MC |  | 0.935 | 0.011 | 0.054 | 1.793 | 0.924 | 0.008 | 0.068 | 1.327 |
|  | Weibull | Delta |  | 0.924 | 0.038 | 0.038 | 1.800 | 0.933 | 0.025 | 0.042 | 1.307 |
|  |  | MC |  | 0.957 | 0.015 | 0.029 | 1.716 | 0.940 | 0.017 | 0.043 | 1.255 |
|  | PMS | Delta |  | 0.817 | 0.097 | 0.086 | 3.031 | 0.881 | 0.075 | 0.044 | 2.296 |
|  |  | MC |  | 0.934 | 0.033 | 0.032 | 2.588 | 0.949 | 0.028 | 0.023 | 2.043 |
|  | PTPRS | Delta |  | 0.906 | 0.059 | 0.035 | 2.590 | 0.929 | 0.046 | 0.025 | 1.959 |
|  | NCS | Delta |  | 0.851 | 0.061 | 0.088 | 2.552 | 0.880 | 0.046 | 0.074 | 1.991 |
|  | WnoF | Delta | 0.005 | 0.919 | 0.014 | 0.067 | 1.393 | 0.916 | 0.008 | 0.076 | 1.000 |
|  |  | MC |  | 0.937 | 0.012 | 0.051 | 1.328 | 0.923 | 0.009 | 0.068 | 0.964 |
|  | Weibull | Delta |  | 0.938 | 0.029 | 0.034 | 1.312 | 0.939 | 0.023 | 0.038 | 0.944 |
|  |  | MC |  | 0.955 | 0.016 | 0.030 | 1.272 | 0.942 | 0.018 | 0.040 | 0.916 |
|  | PMS | Delta |  | 0.877 | 0.075 | 0.048 | 1.947 | 0.921 | 0.055 | 0.023 | 1.414 |
|  |  | MC |  | 0.933 | 0.036 | 0.030 | 1.845 | 0.946 | 0.031 | 0.023 | 1.369 |
|  | PTPRS | Delta |  | 0.929 | 0.045 | 0.026 | 1.732 | 0.942 | 0.036 | 0.022 | 1.282 |
|  | NCS | Delta |  | 0.884 | 0.058 | 0.058 | 1.829 | 0.913 | 0.048 | 0.039 | 1.385 |
|  | WnoF | Delta | 0.010 | 0.928 | 0.014 | 0.058 | 1.077 | 0.924 | 0.008 | 0.068 | 0.769 |
|  |  | MC |  | 0.937 | 0.015 | 0.048 | 1.044 | 0.932 | 0.008 | 0.060 | 0.749 |
|  | Weibull | Delta |  | 0.945 | 0.022 | 0.033 | 1.016 | 0.943 | 0.023 | 0.034 | 0.728 |
|  |  | MC |  | 0.952 | 0.015 | 0.033 | 0.996 | 0.943 | 0.019 | 0.038 | 0.710 |
|  | PMS | Delta |  | 0.911 | 0.057 | 0.032 | 1.371 | 0.937 | 0.043 | 0.020 | 0.978 |
|  |  | MC |  | 0.942 | 0.029 | 0.028 | 1.345 | 0.953 | 0.028 | 0.019 | 0.967 |
|  | PTPRS | Delta |  | 0.940 | 0.034 | 0.026 | 1.250 | 0.955 | 0.027 | 0.018 | 0.916 |
|  | NCS | Delta |  | 0.901 | 0.059 | 0.040 | 1.354 | 0.930 | 0.046 | 0.024 | 0.998 |
| 60% | WnoF | Delta | 0.002 | 0.898 | 0.039 | 0.063 | 2.151 | 0.935 | 0.011 | 0.054 | 1.578 |
|  |  | MC |  | 0.936 | 0.021 | 0.043 | 1.962 | 0.944 | 0.010 | 0.046 | 1.472 |
|  | Weibull | Delta |  | 0.905 | 0.052 | 0.043 | 2.068 | 0.934 | 0.028 | 0.038 | 1.517 |
|  |  | MC |  | 0.948 | 0.025 | 0.026 | 1.954 | 0.955 | 0.011 | 0.034 | 1.456 |
|  | PMS | Delta |  | 0.813 | 0.101 | 0.086 | 3.056 | 0.873 | 0.083 | 0.044 | 2.288 |
|  |  | MC |  | 0.937 | 0.026 | 0.037 | 2.599 | 0.952 | 0.024 | 0.025 | 2.078 |
|  | PTPRS | Delta |  | 0.889 | 0.064 | 0.047 | 2.528 | 0.933 | 0.044 | 0.023 | 2.009 |
|  | NCS | Delta |  | 0.811 | 0.097 | 0.092 | 2.723 | 0.857 | 0.073 | 0.070 | 2.122 |
|  | WnoF | Delta | 0.005 | 0.922 | 0.028 | 0.050 | 1.543 | 0.940 | 0.011 | 0.049 | 1.117 |
|  |  | MC |  | 0.939 | 0.021 | 0.040 | 1.441 | 0.947 | 0.010 | 0.043 | 1.061 |
|  | Weibull | Delta |  | 0.917 | 0.046 | 0.038 | 1.488 | 0.944 | 0.021 | 0.035 | 1.078 |
|  |  | MC |  | 0.951 | 0.021 | 0.027 | 1.440 | 0.954 | 0.012 | 0.034 | 1.058 |
|  | PMS | Delta |  | 0.876 | 0.073 | 0.052 | 1.982 | 0.920 | 0.054 | 0.026 | 1.429 |
|  |  | MC |  | 0.938 | 0.034 | 0.028 | 1.858 | 0.957 | 0.027 | 0.016 | 1.390 |
|  | PTPRS | Delta |  | 0.908 | 0.058 | 0.034 | 1.712 | 0.948 | 0.031 | 0.021 | 1.310 |
|  | NCS | Delta |  | 0.851 | 0.082 | 0.067 | 1.905 | 0.910 | 0.053 | 0.037 | 1.426 |
|  | WnoF | Delta | 0.010 | 0.929 | 0.026 | 0.045 | 1.174 | 0.947 | 0.012 | 0.041 | 0.843 |
|  |  | MC |  | 0.944 | 0.022 | 0.034 | 1.113 | 0.950 | 0.013 | 0.037 | 0.806 |
|  | Weibull | Delta |  | 0.931 | 0.038 | 0.032 | 1.134 | 0.948 | 0.021 | 0.031 | 0.816 |
|  |  | MC |  | 0.951 | 0.020 | 0.028 | 1.114 | 0.954 | 0.012 | 0.034 | 0.813 |
|  | PMS | Delta |  | 0.905 | 0.061 | 0.034 | 1.387 | 0.933 | 0.047 | 0.019 | 1.002 |
|  |  | MC |  | 0.945 | 0.028 | 0.026 | 1.377 | 0.955 | 0.028 | 0.017 | 0.994 |
|  | PTPRS | Delta |  | 0.932 | 0.040 | 0.028 | 1.249 | 0.956 | 0.025 | 0.019 | 0.936 |
|  | NCS | Delta |  | 0.903 | 0.057 | 0.040 | 1.384 | 0.935 | 0.041 | 0.024 | 1.010 |
| 90% | WnoF | Delta | 0.002 | 0.873 | 0.076 | 0.051 | 2.656 | 0.907 | 0.048 | 0.045 | 1.970 |
|  |  | MC |  | 0.937 | 0.033 | 0.030 | 2.439 | 0.944 | 0.025 | 0.031 | 1.845 |
|  | Weibull | Delta |  | 0.872 | 0.078 | 0.050 | 2.702 | 0.904 | 0.052 | 0.044 | 1.954 |
|  |  | MC |  | 0.936 | 0.030 | 0.034 | 2.483 | 0.946 | 0.020 | 0.034 | 1.861 |
|  | PMS | Delta |  | 0.811 | 0.105 | 0.085 | 3.187 | 0.873 | 0.083 | 0.044 | 2.318 |
|  |  | MC |  | 0.944 | 0.027 | 0.029 | 2.800 | 0.950 | 0.023 | 0.027 | 2.153 |
|  | PTPRS | Delta |  | 0.855 | 0.081 | 0.064 | 2.687 | 0.896 | 0.057 | 0.047 | 1.999 |
|  | NCS | Delta |  | 0.747 | 0.169 | 0.084 | 2.408 | 0.828 | 0.126 | 0.046 | 2.090 |
|  | WnoF | Delta | 0.005 | 0.905 | 0.057 | 0.038 | 1.842 | 0.931 | 0.036 | 0.033 | 1.332 |
|  |  | MC |  | 0.935 | 0.030 | 0.035 | 1.785 | 0.943 | 0.028 | 0.029 | 1.294 |
|  | Weibull | Delta |  | 0.905 | 0.057 | 0.038 | 1.921 | 0.932 | 0.036 | 0.032 | 1.323 |
|  |  | MC |  | 0.939 | 0.026 | 0.035 | 1.819 | 0.949 | 0.019 | 0.032 | 1.319 |
|  | PMS | Delta |  | 0.871 | 0.079 | 0.050 | 2.161 | 0.908 | 0.061 | 0.031 | 1.436 |
|  |  | MC |  | 0.940 | 0.034 | 0.026 | 2.021 | 0.952 | 0.027 | 0.021 | 1.471 |
|  | PTPRS | Delta |  | 0.900 | 0.056 | 0.044 | 1.853 | 0.930 | 0.039 | 0.031 | 1.340 |
|  | NCS | Delta |  | 0.802 | 0.123 | 0.075 | 1.772 | 0.842 | 0.101 | 0.057 | 1.361 |
|  | WnoF | Delta | 0.010 | 0.930 | 0.034 | 0.036 | 1.370 | 0.944 | 0.029 | 0.027 | 0.968 |
|  |  | MC |  | 0.936 | 0.029 | 0.035 | 1.382 | 0.945 | 0.027 | 0.028 | 0.967 |
|  | Weibull | Delta |  | 0.929 | 0.034 | 0.037 | 1.403 | 0.945 | 0.029 | 0.026 | 0.964 |
|  |  | MC |  | 0.941 | 0.024 | 0.035 | 1.426 | 0.952 | 0.020 | 0.028 | 0.989 |
|  | PMS | Delta |  | 0.907 | 0.059 | 0.034 | 1.584 | 0.913 | 0.060 | 0.027 | 1.065 |
|  |  | MC |  | 0.948 | 0.026 | 0.025 | 1.551 | 0.951 | 0.031 | 0.019 | 1.094 |
|  | PTPRS | Delta |  | 0.925 | 0.033 | 0.042 | 1.385 | 0.936 | 0.036 | 0.028 | 0.984 |
|  | NCS | Delta |  | 0.797 | 0.115 | 0.087 | 1.257 | 0.848 | 0.091 | 0.061 | 1.044 |

Note: Metrics are calculated based on the logarithms of TTB estimates. The number of MC samples is 2,000. ARR: absolute relative risk; CP: coverage probability; OLLP: out-of-lower-limit probability; OULP: out-of-upper-limit probability; MAE: mean absolute error; RMSE: root mean squared error; MC: Monte Carlo; NCS: natural cubic spline; PMS: penalized M-spline; PTPRS: penalized thin plate regression spline; SPRINT: Systolic Blood Pressure Intervention Trial; TTB: time to benefit; WF: Weibull model with shared frailty; WnoF: Weibull model without shared frailty

**Table S4.** TTB point estimation results for simulation scenario I with the Gamma frailty and 100 replicates.

|  |  |  |  | N=5,000 | | | | N=10,000 | | | |
| --- | --- | --- | --- | --- | --- | --- | --- | --- | --- | --- | --- |
| Cesnoring | Model | Method | ARR | Bias | MAE | RMSE | Time | Bias | MAE | RMSE | Time |
| 0% | WnoF | Delta | 0.002 | -0.119 | 0.390 | 0.472 | <1min | -0.037 | 0.235 | 0.296 | <1min |
|  |  | MC |  | -0.130 | 0.392 | 0.473 | 3min | -0.045 | 0.236 | 0.296 | 3min |
|  | WF | Delta |  | -0.058 | 0.345 | 0.429 | 3min | 0.009 | 0.215 | 0.264 | 5min |
|  |  | MC |  | -0.079 | 0.348 | 0.432 | 14min | -0.007 | 0.214 | 0.263 | 17min |
|  | PMS | Delta |  | -0.098 | 0.626 | 0.769 | 21min | -0.054 | 0.445 | 0.557 | 1hrs51min |
|  |  | MC |  | -0.051 | 0.605 | 0.746 | 1hrs36min | -0.019 | 0.430 | 0.542 | 1hrs7min |
|  | PTPRS | Delta |  | -0.043 | 0.497 | 0.619 | 2hrs33min | -0.036 | 0.344 | 0.439 | 2hrs48min |
|  |  | MC |  | -0.050 | 0.496 | 0.617 | 62hrs23min | -0.048 | 0.348 | 0.445 | 65hrs57min |
|  | NCS | Delta |  | -0.102 | 0.581 | 0.680 | 27min | -0.136 | 0.389 | 0.472 | 29min |
|  |  | MC |  | -0.089 | 0.572 | 0.670 | 21hrs5min | -0.126 | 0.382 | 0.465 | 21hrs1min |
|  | WnoF | Delta | 0.005 | -0.091 | 0.285 | 0.346 |  | -0.036 | 0.169 | 0.213 |  |
|  |  | MC |  | -0.098 | 0.288 | 0.348 |  | -0.041 | 0.170 | 0.214 |  |
|  | Weibull | Delta |  | -0.040 | 0.253 | 0.314 |  | 0.005 | 0.155 | 0.189 |  |
|  |  | MC |  | -0.057 | 0.256 | 0.316 |  | -0.008 | 0.154 | 0.189 |  |
|  | PMS | Delta |  | 0.000 | 0.422 | 0.523 |  | 0.006 | 0.270 | 0.348 |  |
|  |  | MC |  | 0.024 | 0.413 | 0.512 |  | 0.022 | 0.265 | 0.341 |  |
|  | PTPRS | Delta |  | -0.025 | 0.346 | 0.433 |  | -0.016 | 0.227 | 0.289 |  |
|  |  | MC |  | -0.031 | 0.346 | 0.434 |  | -0.022 | 0.229 | 0.292 |  |
|  | NCS | Delta |  | -0.040 | 0.414 | 0.495 |  | -0.049 | 0.260 | 0.321 |  |
|  |  | MC |  | -0.033 | 0.409 | 0.489 |  | -0.045 | 0.257 | 0.318 |  |
|  | WnoF | Delta | 0.010 | -0.068 | 0.219 | 0.267 |  | -0.028 | 0.129 | 0.162 |  |
|  |  | MC |  | -0.072 | 0.221 | 0.268 |  | -0.030 | 0.130 | 0.162 |  |
|  | Weibull | Delta |  | -0.032 | 0.198 | 0.244 |  | 0.002 | 0.119 | 0.145 |  |
|  |  | MC |  | -0.045 | 0.200 | 0.246 |  | -0.009 | 0.119 | 0.145 |  |
|  | PMS | Delta |  | 0.016 | 0.306 | 0.379 |  | 0.009 | 0.185 | 0.237 |  |
|  |  | MC |  | 0.030 | 0.304 | 0.376 |  | 0.017 | 0.185 | 0.236 |  |
|  | PTPRS | Delta |  | -0.023 | 0.258 | 0.324 |  | -0.010 | 0.164 | 0.204 |  |
|  |  | MC |  | -0.027 | 0.258 | 0.325 |  | -0.014 | 0.164 | 0.206 |  |
|  | NCS | Delta |  | -0.021 | 0.308 | 0.376 |  | -0.015 | 0.188 | 0.233 |  |
|  |  | MC |  | -0.019 | 0.306 | 0.372 |  | -0.013 | 0.187 | 0.231 |  |
| 30% | WnoF | Delta | 0.002 | -0.229 | 0.443 | 0.536 | <1min | -0.159 | 0.291 | 0.361 | <1min |
|  |  | MC |  | -0.245 | 0.450 | 0.542 | 3min | -0.170 | 0.295 | 0.366 | 3min |
|  | Weibull | Delta |  | -0.053 | 0.377 | 0.471 | 3min | -0.001 | 0.247 | 0.307 | 4min |
|  |  | MC |  | -0.072 | 0.380 | 0.473 | 14min | -0.014 | 0.246 | 0.306 | 16min |
|  | PMS | Delta |  | -0.076 | 0.613 | 0.750 | 10min | -0.065 | 0.440 | 0.552 | 23min |
|  |  | MC |  | -0.038 | 0.592 | 0.729 | 25min | -0.034 | 0.423 | 0.535 | 1hrs39min |
|  | PTPRS | Delta |  | -0.050 | 0.513 | 0.641 | 2hrs37min | -0.041 | 0.349 | 0.448 | 2hrs49min |
|  |  | MC |  | -0.059 | 0.514 | 0.642 | 65hrs44min | -0.048 | 0.351 | 0.451 | 66hrs34min |
|  | NCS | Delta |  | -0.040 | 0.549 | 0.660 | 28min | -0.100 | 0.395 | 0.501 | 29min |
|  |  | MC |  | -0.030 | 0.542 | 0.651 | 22hrs41min | -0.093 | 0.390 | 0.494 | 21hrs21min |
|  | WnoF | Delta | 0.005 | -0.161 | 0.319 | 0.387 |  | -0.112 | 0.205 | 0.255 |  |
|  |  | MC |  | -0.171 | 0.324 | 0.391 |  | -0.120 | 0.208 | 0.259 |  |
|  | Weibull | Delta |  | -0.037 | 0.274 | 0.344 |  | -0.001 | 0.176 | 0.218 |  |
|  |  | MC |  | -0.051 | 0.277 | 0.345 |  | -0.011 | 0.175 | 0.217 |  |
|  | PMS | Delta |  | 0.003 | 0.415 | 0.512 |  | 0.001 | 0.267 | 0.346 |  |
|  |  | MC |  | 0.024 | 0.412 | 0.506 |  | 0.016 | 0.265 | 0.342 |  |
|  | PTPRS | Delta |  | -0.028 | 0.357 | 0.447 |  | -0.018 | 0.232 | 0.296 |  |
|  |  | MC |  | -0.033 | 0.358 | 0.448 |  | -0.021 | 0.232 | 0.296 |  |
|  | NCS | Delta |  | -0.003 | 0.392 | 0.479 |  | -0.033 | 0.271 | 0.345 |  |
|  |  | MC |  | 0.000 | 0.388 | 0.474 |  | -0.031 | 0.268 | 0.342 |  |
|  | WnoF | Delta | 0.010 | -0.117 | 0.244 | 0.297 |  | -0.081 | 0.152 | 0.191 |  |
|  |  | MC |  | -0.124 | 0.246 | 0.299 |  | -0.086 | 0.154 | 0.193 |  |
|  | Weibull | Delta |  | -0.031 | 0.213 | 0.267 |  | -0.003 | 0.133 | 0.165 |  |
|  |  | MC |  | -0.041 | 0.215 | 0.268 |  | -0.011 | 0.133 | 0.165 |  |
|  | PMS | Delta |  | 0.012 | 0.298 | 0.374 |  | 0.002 | 0.191 | 0.242 |  |
|  |  | MC |  | 0.026 | 0.298 | 0.372 |  | 0.012 | 0.193 | 0.242 |  |
|  | PTPRS | Delta |  | -0.018 | 0.259 | 0.329 |  | -0.011 | 0.168 | 0.210 |  |
|  |  | MC |  | -0.021 | 0.260 | 0.330 |  | -0.013 | 0.168 | 0.210 |  |
|  | NCS | Delta |  | 0.000 | 0.294 | 0.364 |  | -0.010 | 0.195 | 0.247 |  |
|  |  | MC |  | -0.002 | 0.291 | 0.360 |  | -0.012 | 0.194 | 0.245 |  |
| 60% | WnoF | Delta | 0.002 | -0.119 | 0.498 | 0.616 | <1min | -0.112 | 0.304 | 0.386 | <1min |
|  |  | MC |  | -0.129 | 0.496 | 0.614 | 3min | -0.119 | 0.304 | 0.387 | 3min |
|  | Weibull | Delta |  | -0.028 | 0.496 | 0.603 | 2min | -0.015 | 0.288 | 0.366 | 4min |
|  |  | MC |  | -0.056 | 0.495 | 0.604 | 13min | -0.037 | 0.287 | 0.366 | 15min |
|  | PMS | Delta |  | -0.077 | 0.605 | 0.748 | 6min | -0.059 | 0.457 | 0.561 | 12min |
|  |  | MC |  | -0.040 | 0.597 | 0.733 | 22min | -0.030 | 0.449 | 0.551 | 28min |
|  | PTPRS | Delta |  | -0.023 | 0.531 | 0.652 | 2hrs58min | -0.044 | 0.364 | 0.464 | 2hrs48min |
|  |  | MC |  | -0.028 | 0.527 | 0.649 | 66hrs45min | -0.050 | 0.365 | 0.465 | 66hrs56min |
|  | NCS | Delta |  | -0.085 | 0.580 | 0.696 | 28min | -0.090 | 0.462 | 0.558 | 29min |
|  |  | MC |  | -0.081 | 0.569 | 0.683 | 22hrs54min | -0.088 | 0.456 | 0.551 | 22hrs37min |
|  | WnoF | Delta | 0.005 | -0.078 | 0.358 | 0.442 |  | -0.075 | 0.211 | 0.268 |  |
|  |  | MC |  | -0.085 | 0.356 | 0.441 |  | -0.080 | 0.211 | 0.268 |  |
|  | Weibull | Delta |  | -0.019 | 0.355 | 0.434 |  | -0.011 | 0.200 | 0.255 |  |
|  |  | MC |  | -0.039 | 0.356 | 0.435 |  | -0.026 | 0.201 | 0.256 |  |
|  | PMS | Delta |  | 0.004 | 0.415 | 0.514 |  | -0.003 | 0.278 | 0.355 |  |
|  |  | MC |  | 0.028 | 0.411 | 0.507 |  | 0.015 | 0.277 | 0.352 |  |
|  | PTPRS | Delta |  | -0.008 | 0.367 | 0.454 |  | -0.019 | 0.239 | 0.306 |  |
|  |  | MC |  | -0.011 | 0.365 | 0.452 |  | -0.022 | 0.239 | 0.305 |  |
|  | NCS | Delta |  | -0.024 | 0.406 | 0.501 |  | -0.034 | 0.289 | 0.367 |  |
|  |  | MC |  | -0.030 | 0.397 | 0.492 |  | -0.037 | 0.286 | 0.363 |  |
|  | WnoF | Delta | 0.010 | -0.055 | 0.272 | 0.336 |  | -0.052 | 0.154 | 0.198 |  |
|  |  | MC |  | -0.061 | 0.271 | 0.335 |  | -0.056 | 0.155 | 0.198 |  |
|  | Weibull | Delta |  | -0.017 | 0.270 | 0.330 |  | -0.011 | 0.148 | 0.189 |  |
|  |  | MC |  | -0.032 | 0.270 | 0.331 |  | -0.021 | 0.148 | 0.190 |  |
|  | PMS | Delta |  | 0.014 | 0.305 | 0.377 |  | 0.002 | 0.195 | 0.247 |  |
|  |  | MC |  | 0.031 | 0.303 | 0.373 |  | 0.013 | 0.195 | 0.245 |  |
|  | PTPRS | Delta |  | -0.008 | 0.273 | 0.340 |  | -0.013 | 0.174 | 0.218 |  |
|  |  | MC |  | -0.009 | 0.272 | 0.339 |  | -0.015 | 0.173 | 0.217 |  |
|  | NCS | Delta |  | 0.001 | 0.310 | 0.385 |  | -0.011 | 0.195 | 0.252 |  |
|  |  | MC |  | -0.009 | 0.304 | 0.380 |  | -0.018 | 0.193 | 0.249 |  |
| 90% | WnoF | Delta | 0.002 | -0.020 | 0.551 | 0.684 | <1min | -0.052 | 0.374 | 0.483 | <1min |
|  |  | MC |  | -0.015 | 0.545 | 0.677 | 4min | -0.047 | 0.372 | 0.480 | 4min |
|  | Weibull | Delta |  | -0.015 | 0.549 | 0.680 | 2min | -0.047 | 0.373 | 0.481 | 3min |
|  |  | MC |  | -0.041 | 0.553 | 0.687 | 16min | -0.054 | 0.378 | 0.486 | 15min |
|  | PMS | Delta |  | -0.058 | 0.612 | 0.751 | 3min | -0.047 | 0.451 | 0.559 | 7min |
|  |  | MC |  | -0.037 | 0.591 | 0.729 | 22min | -0.025 | 0.435 | 0.543 | 23min |
|  | PTPRS | Delta |  | -0.048 | 0.562 | 0.708 | 2hrs22min | -0.052 | 0.389 | 0.501 | 3hrs24min |
|  |  | MC |  | -0.045 | 0.553 | 0.695 | 63hrs52min | -0.058 | 0.391 | 0.504 | 58hrs10min |
|  | NCS | Delta |  | -0.133 | 0.604 | 0.777 | 29min | -0.057 | 0.442 | 0.558 | 1hrs30min |
|  |  | MC |  | -0.173 | 0.558 | 0.725 | 26hrs45min | -0.092 | 0.414 | 0.526 | 23hrs40min |
|  | WnoF | Delta | 0.005 | -0.005 | 0.386 | 0.487 |  | -0.029 | 0.248 | 0.323 |  |
|  |  | MC |  | -0.006 | 0.379 | 0.480 |  | -0.028 | 0.245 | 0.320 |  |
|  | Weibull | Delta |  | -0.004 | 0.385 | 0.485 |  | -0.027 | 0.247 | 0.322 |  |
|  |  | MC |  | -0.019 | 0.387 | 0.488 |  | -0.031 | 0.246 | 0.322 |  |
|  | PMS | Delta |  | 0.021 | 0.416 | 0.517 |  | 0.001 | 0.283 | 0.365 |  |
|  |  | MC |  | 0.029 | 0.409 | 0.506 |  | 0.010 | 0.281 | 0.362 |  |
|  | PTPRS | Delta |  | -0.001 | 0.380 | 0.484 |  | -0.030 | 0.257 | 0.335 |  |
|  |  | MC |  | -0.002 | 0.377 | 0.481 |  | -0.031 | 0.258 | 0.336 |  |
|  | NCS | Delta |  | -0.046 | 0.422 | 0.528 |  | -0.022 | 0.306 | 0.387 |  |
|  |  | MC |  | -0.117 | 0.402 | 0.508 |  | -0.053 | 0.301 | 0.376 |  |
|  | WnoF | Delta | 0.010 | 0.000 | 0.294 | 0.370 |  | -0.017 | 0.182 | 0.234 |  |
|  |  | MC |  | -0.006 | 0.289 | 0.365 |  | -0.020 | 0.180 | 0.232 |  |
|  | Weibull | Delta |  | -0.001 | 0.293 | 0.369 |  | -0.017 | 0.181 | 0.233 |  |
|  |  | MC |  | -0.010 | 0.293 | 0.370 |  | -0.023 | 0.182 | 0.234 |  |
|  | PMS | Delta |  | 0.033 | 0.312 | 0.384 |  | -0.009 | 0.202 | 0.258 |  |
|  |  | MC |  | 0.040 | 0.309 | 0.380 |  | 0.000 | 0.201 | 0.257 |  |
|  | PTPRS | Delta |  | 0.007 | 0.289 | 0.368 |  | -0.020 | 0.186 | 0.237 |  |
|  |  | MC |  | 0.007 | 0.288 | 0.366 |  | -0.020 | 0.187 | 0.238 |  |
|  | NCS | Delta |  | 0.037 | 0.314 | 0.387 |  | -0.034 | 0.216 | 0.274 |  |
|  |  | MC |  | -0.024 | 0.298 | 0.370 |  | -0.053 | 0.222 | 0.282 |  |

Note: Metrics are calculated based on the logarithms of TTB estimates. The number of MC samples is 2,000. For MC method, the estimates are the median of the MC samples. Time is for all ARR values for each Model. ARR: absolute relative risk; MAE: mean absolute error; RMSE: root mean squared error; MC: Monte Carlo; NCS: natural cubic spline; PMS: penalized M-spline; PTPRS: penalized thin plate regression spline; SPRINT: Systolic Blood Pressure Intervention Trial; TTB: time to benefit; WF: Weibull model with shared frailty; WnoF: Weibull model without shared frailty

**Table S5.** TTB interval estimation results for simulation scenario I with the Gamma frailty and 100 replicates.

|  |  |  |  | N=5,000 | | | | N=10,000 | | | |
| --- | --- | --- | --- | --- | --- | --- | --- | --- | --- | --- | --- |
| Cesnoring | Model | Method | ARR | CP | OLLP | OULP | length | CP | OLLP | OULP | length |
| 0% | WnoF | Delta | 0.002 | 0.900 | 0.050 | 0.050 | 1.777 | 0.970 | 0.020 | 0.010 | 1.286 |
|  |  | MC |  | 0.950 | 0.040 | 0.010 | 1.721 | 0.990 | 0.010 | 0.000 | 1.266 |
|  | WF | Delta |  | 0.910 | 0.050 | 0.040 | 1.612 | 0.980 | 0.010 | 0.010 | 1.173 |
|  |  | MC |  | 0.940 | 0.040 | 0.020 | 1.560 | 0.990 | 0.010 | 0.000 | 1.149 |
|  | PMS | Delta |  | 0.800 | 0.090 | 0.110 | 3.223 | 0.857 | 0.092 | 0.051 | 2.312 |
|  |  | MC |  | 0.920 | 0.040 | 0.040 | 2.578 | 0.939 | 0.010 | 0.051 | 2.011 |
|  | PTPRS | Delta |  | 0.880 | 0.060 | 0.060 | 2.477 | 0.970 | 0.010 | 0.020 | 1.940 |
|  |  | MC |  | 0.940 | 0.030 | 0.030 | 2.365 | 0.970 | 0.000 | 0.030 | 1.891 |
|  | NCS | Delta |  | 0.810 | 0.080 | 0.110 | 2.287 | 0.850 | 0.040 | 0.110 | 1.837 |
|  |  | MC |  | 0.890 | 0.030 | 0.080 | 2.012 | 0.930 | 0.010 | 0.060 | 1.656 |
|  | WnoF | Delta | 0.005 | 0.920 | 0.040 | 0.040 | 1.300 | 0.980 | 0.010 | 0.010 | 0.936 |
|  |  | MC |  | 0.950 | 0.040 | 0.010 | 1.289 | 0.990 | 0.010 | 0.000 | 0.938 |
|  | WF | Delta |  | 0.930 | 0.040 | 0.030 | 1.181 | 0.990 | 0.010 | 0.000 | 0.854 |
|  |  | MC |  | 0.940 | 0.040 | 0.020 | 1.161 | 0.990 | 0.010 | 0.000 | 0.841 |
|  | PMS | Delta |  | 0.820 | 0.090 | 0.090 | 1.992 | 0.898 | 0.051 | 0.051 | 1.412 |
|  |  | MC |  | 0.910 | 0.040 | 0.050 | 1.822 | 0.949 | 0.020 | 0.031 | 1.342 |
|  | PTPRS | Delta |  | 0.930 | 0.030 | 0.040 | 1.654 | 0.970 | 0.010 | 0.020 | 1.262 |
|  |  | MC |  | 0.930 | 0.030 | 0.040 | 1.616 | 0.970 | 0.000 | 0.030 | 1.248 |
|  | NCS | Delta |  | 0.840 | 0.070 | 0.090 | 1.674 | 0.920 | 0.030 | 0.050 | 1.311 |
|  |  | MC |  | 0.890 | 0.040 | 0.070 | 1.508 | 0.950 | 0.020 | 0.030 | 1.204 |
|  | WnoF | Delta | 0.010 | 0.930 | 0.040 | 0.030 | 1.012 | 0.990 | 0.010 | 0.000 | 0.724 |
|  |  | MC |  | 0.950 | 0.040 | 0.010 | 1.008 | 0.990 | 0.010 | 0.000 | 0.727 |
|  | WF | Delta |  | 0.930 | 0.040 | 0.030 | 0.923 | 0.990 | 0.010 | 0.000 | 0.664 |
|  |  | MC |  | 0.960 | 0.030 | 0.010 | 0.916 | 0.990 | 0.010 | 0.000 | 0.659 |
|  | PMS | Delta |  | 0.860 | 0.070 | 0.070 | 1.326 | 0.949 | 0.031 | 0.020 | 0.945 |
|  |  | MC |  | 0.880 | 0.050 | 0.070 | 1.305 | 0.959 | 0.020 | 0.020 | 0.938 |
|  | PTPRS | Delta |  | 0.910 | 0.030 | 0.060 | 1.194 | 0.970 | 0.010 | 0.020 | 0.900 |
|  |  | MC |  | 0.910 | 0.030 | 0.060 | 1.170 | 0.970 | 0.000 | 0.030 | 0.885 |
|  | NCS | Delta |  | 0.850 | 0.070 | 0.080 | 1.269 | 0.940 | 0.040 | 0.020 | 0.974 |
|  |  | MC |  | 0.890 | 0.050 | 0.060 | 1.163 | 0.950 | 0.030 | 0.020 | 0.905 |
| 30% | WnoF | Delta | 0.002 | 0.890 | 0.020 | 0.090 | 1.906 | 0.910 | 0.010 | 0.080 | 1.391 |
|  |  | MC |  | 0.910 | 0.020 | 0.070 | 1.783 | 0.930 | 0.010 | 0.060 | 1.326 |
|  | WF | Delta |  | 0.918 | 0.031 | 0.051 | 1.787 | 0.940 | 0.020 | 0.040 | 1.304 |
|  |  | MC |  | 0.939 | 0.020 | 0.041 | 1.704 | 0.950 | 0.010 | 0.040 | 1.253 |
|  | PMS | Delta |  | 0.850 | 0.070 | 0.080 | 3.152 | 0.860 | 0.080 | 0.060 | 2.384 |
|  |  | MC |  | 0.910 | 0.040 | 0.050 | 2.627 | 0.950 | 0.010 | 0.040 | 2.062 |
|  | PTPRS | Delta |  | 0.880 | 0.060 | 0.060 | 2.523 | 0.940 | 0.040 | 0.020 | 1.972 |
|  |  | MC |  | 0.940 | 0.020 | 0.040 | 2.421 | 0.980 | 0.000 | 0.020 | 1.926 |
|  | NCS | Delta |  | 0.820 | 0.080 | 0.100 | 2.508 | 0.850 | 0.040 | 0.110 | 1.990 |
|  |  | MC |  | 0.900 | 0.030 | 0.070 | 2.135 | 0.910 | 0.020 | 0.070 | 1.747 |
|  | WnoF | Delta | 0.005 | 0.900 | 0.020 | 0.080 | 1.383 | 0.920 | 0.010 | 0.070 | 0.999 |
|  |  | MC |  | 0.920 | 0.020 | 0.060 | 1.319 | 0.930 | 0.010 | 0.060 | 0.963 |
|  | WF | Delta |  | 0.918 | 0.031 | 0.051 | 1.301 | 0.970 | 0.010 | 0.020 | 0.941 |
|  |  | MC |  | 0.929 | 0.020 | 0.051 | 1.262 | 0.970 | 0.010 | 0.020 | 0.913 |
|  | PMS | Delta |  | 0.840 | 0.090 | 0.070 | 1.959 | 0.920 | 0.040 | 0.040 | 1.429 |
|  |  | MC |  | 0.890 | 0.040 | 0.070 | 1.858 | 0.930 | 0.030 | 0.040 | 1.371 |
|  | PTPRS | Delta |  | 0.930 | 0.030 | 0.040 | 1.692 | 0.970 | 0.010 | 0.020 | 1.280 |
|  |  | MC |  | 0.940 | 0.020 | 0.040 | 1.663 | 0.970 | 0.000 | 0.030 | 1.272 |
|  | NCS | Delta |  | 0.840 | 0.070 | 0.090 | 1.782 | 0.870 | 0.050 | 0.080 | 1.371 |
|  |  | MC |  | 0.890 | 0.040 | 0.070 | 1.582 | 0.920 | 0.030 | 0.050 | 1.248 |
|  | WnoF | Delta | 0.010 | 0.910 | 0.020 | 0.070 | 1.070 | 0.920 | 0.010 | 0.070 | 0.767 |
|  |  | MC |  | 0.920 | 0.020 | 0.060 | 1.036 | 0.930 | 0.010 | 0.060 | 0.747 |
|  | WF | Delta |  | 0.929 | 0.020 | 0.051 | 1.008 | 0.980 | 0.010 | 0.010 | 0.725 |
|  |  | MC |  | 0.929 | 0.020 | 0.051 | 0.989 | 0.960 | 0.010 | 0.030 | 0.708 |
|  | PMS | Delta |  | 0.850 | 0.070 | 0.080 | 1.349 | 0.940 | 0.020 | 0.040 | 0.960 |
|  |  | MC |  | 0.900 | 0.030 | 0.070 | 1.324 | 0.960 | 0.020 | 0.020 | 0.956 |
|  | PTPRS | Delta |  | 0.910 | 0.030 | 0.060 | 1.230 | 0.970 | 0.010 | 0.020 | 0.912 |
|  |  | MC |  | 0.900 | 0.020 | 0.080 | 1.208 | 0.970 | 0.010 | 0.020 | 0.910 |
|  | NCS | Delta |  | 0.860 | 0.060 | 0.080 | 1.309 | 0.920 | 0.040 | 0.040 | 0.986 |
|  |  | MC |  | 0.880 | 0.050 | 0.070 | 1.206 | 0.950 | 0.010 | 0.040 | 0.922 |
| 60% | WnoF | Delta | 0.002 | 0.850 | 0.040 | 0.110 | 2.104 | 0.940 | 0.010 | 0.050 | 1.572 |
|  |  | MC |  | 0.910 | 0.030 | 0.060 | 1.928 | 0.950 | 0.010 | 0.040 | 1.467 |
|  | WF | Delta |  | 0.876 | 0.052 | 0.072 | 2.014 | 0.930 | 0.030 | 0.040 | 1.510 |
|  |  | MC |  | 0.918 | 0.031 | 0.052 | 1.917 | 0.950 | 0.010 | 0.040 | 1.451 |
|  | PMS | Delta |  | 0.810 | 0.110 | 0.080 | 3.175 | 0.820 | 0.110 | 0.070 | 2.334 |
|  |  | MC |  | 0.930 | 0.030 | 0.040 | 2.619 | 0.950 | 0.010 | 0.040 | 2.095 |
|  | PTPRS | Delta |  | 0.860 | 0.060 | 0.080 | 2.437 | 0.930 | 0.040 | 0.030 | 2.023 |
|  |  | MC |  | 0.920 | 0.020 | 0.060 | 2.332 | 0.970 | 0.000 | 0.030 | 1.988 |
|  | NCS | Delta |  | 0.790 | 0.100 | 0.110 | 2.791 | 0.810 | 0.100 | 0.090 | 2.114 |
|  |  | MC |  | 0.900 | 0.040 | 0.060 | 2.336 | 0.930 | 0.010 | 0.060 | 1.855 |
|  | WnoF | Delta | 0.005 | 0.890 | 0.030 | 0.080 | 1.518 | 0.950 | 0.010 | 0.040 | 1.112 |
|  |  | MC |  | 0.910 | 0.030 | 0.060 | 1.420 | 0.950 | 0.010 | 0.040 | 1.056 |
|  | WF | Delta |  | 0.887 | 0.041 | 0.072 | 1.459 | 0.950 | 0.010 | 0.040 | 1.073 |
|  |  | MC |  | 0.907 | 0.031 | 0.062 | 1.417 | 0.950 | 0.010 | 0.040 | 1.054 |
|  | PMS | Delta |  | 0.870 | 0.070 | 0.060 | 2.024 | 0.900 | 0.050 | 0.050 | 1.434 |
|  |  | MC |  | 0.920 | 0.040 | 0.040 | 1.861 | 0.940 | 0.020 | 0.040 | 1.388 |
|  | PTPRS | Delta |  | 0.890 | 0.050 | 0.060 | 1.657 | 0.960 | 0.010 | 0.030 | 1.307 |
|  |  | MC |  | 0.910 | 0.030 | 0.060 | 1.624 | 0.960 | 0.010 | 0.030 | 1.302 |
|  | NCS | Delta |  | 0.820 | 0.080 | 0.100 | 2.005 | 0.890 | 0.050 | 0.060 | 1.426 |
|  |  | MC |  | 0.890 | 0.040 | 0.070 | 1.731 | 0.940 | 0.020 | 0.040 | 1.320 |
|  | WnoF | Delta | 0.010 | 0.910 | 0.030 | 0.060 | 1.158 | 0.950 | 0.010 | 0.040 | 0.839 |
|  |  | MC |  | 0.910 | 0.030 | 0.060 | 1.099 | 0.950 | 0.010 | 0.040 | 0.803 |
|  | WF | Delta |  | 0.897 | 0.041 | 0.062 | 1.116 | 0.950 | 0.010 | 0.040 | 0.812 |
|  |  | MC |  | 0.907 | 0.031 | 0.062 | 1.098 | 0.950 | 0.010 | 0.040 | 0.810 |
|  | PMS | Delta |  | 0.860 | 0.080 | 0.060 | 1.363 | 0.940 | 0.020 | 0.040 | 0.978 |
|  |  | MC |  | 0.910 | 0.030 | 0.060 | 1.357 | 0.950 | 0.020 | 0.030 | 0.982 |
|  | PTPRS | Delta |  | 0.880 | 0.050 | 0.070 | 1.214 | 0.960 | 0.010 | 0.030 | 0.927 |
|  |  | MC |  | 0.910 | 0.030 | 0.060 | 1.196 | 0.970 | 0.010 | 0.020 | 0.931 |
|  | NCS | Delta |  | 0.840 | 0.080 | 0.080 | 1.397 | 0.930 | 0.030 | 0.040 | 1.020 |
|  |  | MC |  | 0.880 | 0.040 | 0.080 | 1.319 | 0.950 | 0.010 | 0.040 | 0.970 |
| 90% | WnoF | Delta | 0.002 | 0.870 | 0.060 | 0.070 | 2.595 | 0.910 | 0.030 | 0.060 | 1.954 |
|  |  | MC |  | 0.940 | 0.030 | 0.030 | 2.417 | 0.970 | 0.000 | 0.030 | 1.832 |
|  | WF | Delta |  | 0.870 | 0.060 | 0.070 | 2.575 | 0.910 | 0.030 | 0.060 | 1.941 |
|  |  | MC |  | 0.940 | 0.020 | 0.040 | 2.459 | 0.970 | 0.000 | 0.030 | 1.853 |
|  | PMS | Delta |  | 0.810 | 0.110 | 0.080 | 3.196 | 0.850 | 0.090 | 0.060 | 2.445 |
|  |  | MC |  | 0.960 | 0.030 | 0.010 | 2.736 | 0.930 | 0.020 | 0.050 | 2.134 |
|  | PTPRS | Delta |  | 0.860 | 0.070 | 0.070 | 2.726 | 0.880 | 0.050 | 0.070 | 1.997 |
|  |  | MC |  | 0.940 | 0.020 | 0.040 | 2.542 | 0.960 | 0.000 | 0.040 | 1.942 |
|  | NCS | Delta |  | 0.750 | 0.170 | 0.080 | 2.187 | 0.820 | 0.140 | 0.040 | 2.119 |
|  |  | MC |  | 0.960 | 0.030 | 0.010 | 2.892 | 0.920 | 0.030 | 0.050 | 2.492 |
|  | WnoF | Delta | 0.005 | 0.900 | 0.060 | 0.040 | 1.799 | 0.960 | 0.010 | 0.030 | 1.322 |
|  |  | MC |  | 0.930 | 0.030 | 0.040 | 1.766 | 0.960 | 0.010 | 0.030 | 1.284 |
|  | WF | Delta |  | 0.900 | 0.060 | 0.040 | 1.788 | 0.960 | 0.010 | 0.030 | 1.316 |
|  |  | MC |  | 0.930 | 0.030 | 0.040 | 1.801 | 0.970 | 0.000 | 0.030 | 1.311 |
|  | PMS | Delta |  | 0.860 | 0.090 | 0.050 | 2.066 | 0.870 | 0.070 | 0.060 | 1.407 |
|  |  | MC |  | 0.930 | 0.040 | 0.030 | 1.996 | 0.940 | 0.020 | 0.040 | 1.461 |
|  | PTPRS | Delta |  | 0.890 | 0.060 | 0.050 | 1.846 | 0.950 | 0.020 | 0.030 | 1.329 |
|  |  | MC |  | 0.930 | 0.030 | 0.040 | 1.821 | 0.970 | 0.000 | 0.030 | 1.332 |
|  | NCS | Delta |  | 0.760 | 0.170 | 0.070 | 1.629 | 0.760 | 0.140 | 0.100 | 1.250 |
|  |  | MC |  | 0.930 | 0.030 | 0.040 | 2.218 | 0.930 | 0.010 | 0.060 | 1.851 |
|  | WnoF | Delta | 0.010 | 0.920 | 0.040 | 0.040 | 1.334 | 0.960 | 0.010 | 0.030 | 0.960 |
|  |  | MC |  | 0.930 | 0.030 | 0.040 | 1.365 | 0.960 | 0.010 | 0.030 | 0.958 |
|  | WF | Delta |  | 0.920 | 0.040 | 0.040 | 1.327 | 0.960 | 0.010 | 0.030 | 0.958 |
|  |  | MC |  | 0.940 | 0.020 | 0.040 | 1.406 | 0.960 | 0.010 | 0.030 | 0.982 |
|  | PMS | Delta |  | 0.880 | 0.080 | 0.040 | 1.403 | 0.910 | 0.060 | 0.030 | 1.021 |
|  |  | MC |  | 0.940 | 0.030 | 0.030 | 1.553 | 0.950 | 0.030 | 0.020 | 1.069 |
|  | PTPRS | Delta |  | 0.910 | 0.030 | 0.060 | 1.350 | 0.970 | 0.010 | 0.020 | 0.979 |
|  |  | MC |  | 0.930 | 0.020 | 0.050 | 1.372 | 0.960 | 0.010 | 0.030 | 0.985 |
|  | NCS | Delta |  | 0.780 | 0.150 | 0.070 | 1.239 | 0.900 | 0.050 | 0.050 | 1.082 |
|  |  | MC |  | 0.920 | 0.010 | 0.070 | 1.869 | 0.940 | 0.020 | 0.040 | 1.489 |

Note: Metrics are calculated based on the logarithms of TTB estimates. The number of MC samples is 2,000. ARR: absolute relative risk; CP: coverage probability; OLLP: out-of-lower-limit probability; OULP: out-of-upper-limit probability; MAE: mean absolute error; RMSE: root mean squared error; MC: Monte Carlo; NCS: natural cubic spline; PMS: penalized M-spline; PTPRS: penalized thin plate regression spline; SPRINT: Systolic Blood Pressure Intervention Trial; TTB: time to benefit; WF: Weibull model with shared frailty; WnoF: Weibull model without shared frailty

**Table S6.** TTB point estimation results for simulation scenario I with the log-normal frailty and 1,000 replicates.

|  |  |  |  | N=5,000 | | | | N=10,000 | | | |
| --- | --- | --- | --- | --- | --- | --- | --- | --- | --- | --- | --- |
| Cesnoring | Model | Method | ARR | Bias | MAE | RMSE | Time | Bias | MAE | RMSE | Time |
| 0% | WnoF | Delta | 0.002 | -0.032 | 0.370 | 0.459 | 1min | -0.025 | 0.261 | 0.325 | 1min |
|  |  | MC |  | -0.022 | 0.371 | 0.459 | 1hrs31min | -0.018 | 0.261 | 0.325 | 1hrs30min |
|  | WF | Delta |  | -0.086 | 0.462 | 0.625 | 3hrs11min | 0.009 | 0.247 | 0.307 | 6hrs40min |
|  |  | MC |  | -0.089 | 0.466 | 0.634 | 6hrs54min | 0.011 | 0.247 | 0.308 | 9hrs46min |
|  | PMS | Delta |  | -0.358 | 0.679 | 0.800 | 16hrs25min | -0.295 | 0.561 | 0.690 | 32hrs24min |
|  |  | MC |  | -0.359 | 0.674 | 0.795 | 19hrs28min | -0.287 | 0.556 | 0.682 | 36hrs42min |
|  | PTPRS | Delta |  | -0.018 | 0.481 | 0.602 | 29hrs38min | -0.025 | 0.376 | 0.467 | 46hrs39min |
|  | NCS | Delta |  | -0.370 | 0.452 | 0.526 | 3hrs35min | -0.390 | 0.422 | 0.479 | 4hrs48min |
|  | WnoF | Delta | 0.005 | -0.025 | 0.271 | 0.336 |  | -0.022 | 0.190 | 0.236 |  |
|  |  | MC |  | -0.018 | 0.272 | 0.337 |  | -0.017 | 0.190 | 0.237 |  |
|  | WF | Delta |  | -0.111 | 0.437 | 0.657 |  | 0.005 | 0.183 | 0.232 |  |
|  |  | MC |  | -0.109 | 0.434 | 0.651 |  | 0.006 | 0.184 | 0.232 |  |
|  | PMS | Delta |  | -0.116 | 0.431 | 0.523 |  | 0.071 | 0.374 | 0.469 |  |
|  |  | MC |  | -0.119 | 0.428 | 0.519 |  | 0.080 | 0.368 | 0.459 |  |
|  | PTPRS | Delta |  | -0.001 | 0.324 | 0.410 |  | -0.006 | 0.249 | 0.310 |  |
|  | NCS | Delta |  | -0.196 | 0.297 | 0.357 |  | -0.205 | 0.255 | 0.300 |  |
|  | WnoF | Delta | 0.010 | -0.017 | 0.210 | 0.261 |  | -0.014 | 0.147 | 0.182 |  |
|  |  | MC |  | -0.011 | 0.211 | 0.262 |  | -0.010 | 0.147 | 0.183 |  |
|  | WF | Delta |  | -0.077 | 0.421 | 0.668 |  | 0.001 | 0.147 | 0.189 |  |
|  |  | MC |  | -0.082 | 0.419 | 0.665 |  | 0.001 | 0.147 | 0.189 |  |
|  | PMS | Delta |  | 0.001 | 0.312 | 0.386 |  | 0.309 | 0.415 | 0.521 |  |
|  |  | MC |  | -0.004 | 0.310 | 0.383 |  | 0.309 | 0.413 | 0.515 |  |
|  | PTPRS | Delta |  | 0.003 | 0.236 | 0.299 |  | 0.000 | 0.177 | 0.222 |  |
|  | NCS | Delta |  | -0.109 | 0.221 | 0.272 |  | -0.112 | 0.176 | 0.212 |  |
| 30% | WnoF | Delta | 0.002 | -0.123 | 0.401 | 0.500 | 1min | -0.107 | 0.291 | 0.365 | 1min |
|  |  | MC |  | -0.109 | 0.398 | 0.495 | 1hrs31min | -0.097 | 0.288 | 0.362 | 1hrs32min |
|  | WF | Delta |  | -0.002 | 0.376 | 0.466 | 3hrs4min | -0.127 | 0.532 | 0.720 | 6hrs26min |
|  |  | MC |  | 0.013 | 0.377 | 0.467 | 6hrs21min | -0.122 | 0.532 | 0.721 | 9hrs8min |
|  | PMS | Delta |  | -0.213 | 0.664 | 0.783 | 13hrs21min | -0.171 | 0.505 | 0.612 | 27hrs38min |
|  |  | MC |  | -0.217 | 0.657 | 0.776 | 17hrs7min | -0.174 | 0.501 | 0.608 | 30hrs53min |
|  | PTPRS | Delta |  | -0.022 | 0.492 | 0.621 | 33hrs15min | -0.009 | 0.368 | 0.459 | 42hrs36min |
|  | NCS | Delta |  | -0.307 | 0.456 | 0.539 | 3hrs59min | -0.325 | 0.401 | 0.469 | 3hrs21min |
|  | WnoF | Delta | 0.005 | -0.084 | 0.290 | 0.362 |  | -0.074 | 0.210 | 0.263 |  |
|  |  | MC |  | -0.074 | 0.288 | 0.360 |  | -0.066 | 0.208 | 0.261 |  |
|  | WF | Delta |  | 0.001 | 0.276 | 0.348 |  | -0.149 | 0.596 | 0.824 |  |
|  |  | MC |  | 0.010 | 0.277 | 0.349 |  | -0.150 | 0.593 | 0.819 |  |
|  | PMS | Delta |  | -0.051 | 0.425 | 0.516 |  | 0.012 | 0.327 | 0.399 |  |
|  |  | MC |  | -0.057 | 0.422 | 0.512 |  | 0.008 | 0.324 | 0.396 |  |
|  | PTPRS | Delta |  | -0.003 | 0.331 | 0.421 |  | 0.002 | 0.245 | 0.306 |  |
|  | NCS | Delta |  | -0.153 | 0.310 | 0.377 |  | -0.161 | 0.253 | 0.304 |  |
|  | WnoF | Delta | 0.010 | -0.058 | 0.223 | 0.279 |  | -0.051 | 0.160 | 0.201 |  |
|  |  | MC |  | -0.051 | 0.221 | 0.277 |  | -0.046 | 0.159 | 0.199 |  |
|  | WF | Delta |  | 0.002 | 0.216 | 0.278 |  | -0.105 | 0.623 | 0.856 |  |
|  |  | MC |  | 0.008 | 0.217 | 0.279 |  | -0.107 | 0.619 | 0.850 |  |
|  | PMS | Delta |  | 0.013 | 0.294 | 0.365 |  | 0.086 | 0.249 | 0.306 |  |
|  |  | MC |  | 0.006 | 0.292 | 0.361 |  | 0.082 | 0.247 | 0.304 |  |
|  | PTPRS | Delta |  | 0.001 | 0.239 | 0.305 |  | 0.004 | 0.177 | 0.221 |  |
|  | NCS | MC |  | -0.077 | 0.236 | 0.291 |  | -0.080 | 0.183 | 0.222 |  |
| 60% | WnoF | Delta | 0.002 | -0.097 | 0.455 | 0.562 | 1min | -0.095 | 0.339 | 0.416 | 1min |
|  |  | Delta |  | -0.088 | 0.454 | 0.560 | 1hrs31min | -0.088 | 0.337 | 0.414 | 1hrs31min |
|  | WF | Delta |  | -0.004 | 0.436 | 0.539 | 3hrs57min | -0.015 | 0.441 | 0.600 | 8hrs39min |
|  |  | MC |  | 0.018 | 0.440 | 0.543 | 6hrs9min | 0.006 | 0.440 | 0.596 | 10hrs18min |
|  | PMS | Delta |  | -0.089 | 0.638 | 0.770 | 9hrs29min | -0.056 | 0.499 | 0.604 | 19hrs15min |
|  |  | MC |  | -0.101 | 0.629 | 0.761 | 13hrs17min | -0.064 | 0.495 | 0.599 | 22hrs23min |
|  | PTPRS | Delta |  | -0.018 | 0.506 | 0.638 | 61hrs51min | -0.026 | 0.398 | 0.496 | 44hrs16min |
|  | NCS | MC |  | -0.182 | 0.490 | 0.588 | 3hrs53min | -0.196 | 0.398 | 0.477 | 4hrs34min |
|  | WnoF | Delta | 0.005 | -0.059 | 0.324 | 0.402 |  | -0.058 | 0.238 | 0.293 |  |
|  |  | Delta |  | -0.052 | 0.322 | 0.401 |  | -0.054 | 0.237 | 0.292 |  |
|  | WF | Delta |  | 0.001 | 0.312 | 0.389 |  | 0.065 | 0.401 | 0.593 |  |
|  |  | MC |  | 0.018 | 0.315 | 0.393 |  | 0.082 | 0.398 | 0.580 |  |
|  | PMS | Delta |  | -0.007 | 0.405 | 0.505 |  | 0.020 | 0.309 | 0.379 |  |
|  |  | MC |  | -0.020 | 0.399 | 0.498 |  | 0.011 | 0.306 | 0.375 |  |
|  | PTPRS | Delta |  | -0.003 | 0.340 | 0.433 |  | -0.004 | 0.262 | 0.325 |  |
|  | NCS | MC |  | -0.074 | 0.344 | 0.423 |  | -0.077 | 0.269 | 0.327 |  |
|  | WnoF | Delta | 0.010 | -0.038 | 0.245 | 0.306 |  | -0.036 | 0.179 | 0.220 |  |
|  |  | Delta |  | -0.032 | 0.245 | 0.306 |  | -0.032 | 0.179 | 0.220 |  |
|  | WF | Delta |  | 0.001 | 0.237 | 0.297 |  | 0.138 | 0.373 | 0.574 |  |
|  |  | MC |  | 0.014 | 0.238 | 0.299 |  | 0.150 | 0.370 | 0.568 |  |
|  | PMS | Delta |  | 0.016 | 0.275 | 0.351 |  | 0.033 | 0.206 | 0.256 |  |
|  |  | MC |  | 0.001 | 0.270 | 0.346 |  | 0.023 | 0.203 | 0.253 |  |
|  | PTPRS | Delta |  | -0.002 | 0.247 | 0.316 |  | 0.000 | 0.187 | 0.233 |  |
|  | NCS | MC |  | -0.027 | 0.261 | 0.326 |  | -0.025 | 0.200 | 0.245 |  |
| 90% | WnoF | Delta | 0.002 | -0.038 | 0.563 | 0.697 | 1min | -0.021 | 0.412 | 0.508 | 1min |
|  |  | Delta |  | -0.027 | 0.563 | 0.696 | 1hrs37min | -0.013 | 0.413 | 0.508 | 1hrs34min |
|  | WF | Delta |  | -0.032 | 0.560 | 0.693 | 2hrs29min | -0.015 | 0.410 | 0.504 | 5hrs11min |
|  |  | MC |  | -0.009 | 0.562 | 0.694 | 6hrs22min | 0.000 | 0.413 | 0.506 | 8hrs10min |
|  | PMS | Delta |  | -0.053 | 0.631 | 0.773 | 7hrs45min | -0.028 | 0.486 | 0.593 | 14hrs30min |
|  |  | MC |  | -0.096 | 0.635 | 0.777 | 13hrs13min | -0.056 | 0.490 | 0.600 | 18hrs39min |
|  | PTPRS | Delta |  | -0.036 | 0.576 | 0.721 | 137hrs30min | -0.020 | 0.422 | 0.523 | 222hrs59min |
|  | NCS | MC |  | -0.046 | 0.587 | 0.710 | 2hrs30min | -0.035 | 0.473 | 0.568 | 3hrs11min |
|  | WnoF | Delta | 0.005 | -0.012 | 0.385 | 0.486 |  | -0.004 | 0.279 | 0.346 |  |
|  |  | Delta |  | -0.004 | 0.386 | 0.485 |  | 0.003 | 0.281 | 0.348 |  |
|  | WF | Delta |  | -0.010 | 0.383 | 0.484 |  | -0.003 | 0.278 | 0.345 |  |
|  |  | MC |  | 0.009 | 0.386 | 0.485 |  | 0.011 | 0.280 | 0.347 |  |
|  | PMS | Delta |  | -0.001 | 0.403 | 0.516 |  | 0.012 | 0.305 | 0.377 |  |
|  |  | MC |  | -0.021 | 0.409 | 0.525 |  | 0.000 | 0.309 | 0.382 |  |
|  | PTPRS | Delta |  | -0.008 | 0.388 | 0.492 |  | -0.003 | 0.282 | 0.351 |  |
|  | NCS | MC |  | -0.013 | 0.394 | 0.497 |  | -0.004 | 0.308 | 0.379 |  |
|  | WnoF | Delta | 0.010 | 0.000 | 0.280 | 0.361 |  | 0.002 | 0.204 | 0.253 |  |
|  |  | Delta |  | 0.006 | 0.280 | 0.358 |  | 0.009 | 0.204 | 0.253 |  |
|  | WF | Delta |  | -0.001 | 0.280 | 0.360 |  | 0.001 | 0.203 | 0.252 |  |
|  |  | MC |  | 0.015 | 0.282 | 0.359 |  | 0.014 | 0.206 | 0.255 |  |
|  | PMS | Delta |  | 0.011 | 0.283 | 0.370 |  | 0.013 | 0.211 | 0.263 |  |
|  |  | MC |  | 0.008 | 0.289 | 0.390 |  | 0.015 | 0.218 | 0.288 |  |
|  | PTPRS | Delta |  | 0.001 | 0.281 | 0.364 |  | 0.001 | 0.205 | 0.255 |  |
|  | NCS | MC |  | -0.001 | 0.285 | 0.369 |  | 0.004 | 0.212 | 0.267 |  |

Note: Metrics are calculated based on the logarithms of TTB estimates. The number of MC samples is 2,000. For MC method, the estimates are the median of the MC samples. Time is for all ARR values for each Model. ARR: absolute relative risk; MAE: mean absolute error; RMSE: root mean squared error; MC: Monte Carlo; NCS: natural cubic spline; PMS: penalized M-spline; PTPRS: penalized thin plate regression spline; SPRINT: Systolic Blood Pressure Intervention Trial; TTB: time to benefit; WF: Weibull model with shared frailty; WnoF: Weibull model without shared frailty

**Table S7.** TTB interval estimation results for simulation scenario I with the log-normal frailty and 1,000 replicates.

|  |  |  |  | N=5,000 | | | | N=10,000 | | | |
| --- | --- | --- | --- | --- | --- | --- | --- | --- | --- | --- | --- |
| Cesnoring | Model | Method | ARR | CP | OLLP | OULP | length | CP | OLLP | OULP | length |
| 0% | WnoF | Delta | 0.002 | 0.925 | 0.028 | 0.047 | 1.804 | 0.945 | 0.027 | 0.028 | 1.302 |
|  |  | MC |  | 0.948 | 0.016 | 0.036 | 1.722 | 0.958 | 0.019 | 0.023 | 1.264 |
|  | WF | Delta |  | 0.910 | 0.054 | 0.035 | 2.023 | 0.942 | 0.035 | 0.023 | 1.206 |
|  |  | MC |  | 0.934 | 0.032 | 0.033 | 2.134 | 0.952 | 0.029 | 0.019 | 1.190 |
|  | PMS | Delta |  | 0.805 | 0.029 | 0.166 | 2.848 | 0.830 | 0.033 | 0.137 | 2.287 |
|  |  | MC |  | 0.929 | 0.004 | 0.067 | 2.472 | 0.922 | 0.006 | 0.071 | 2.091 |
|  | PTPRS | Delta |  | 0.906 | 0.061 | 0.033 | 2.524 | 0.932 | 0.052 | 0.016 | 1.922 |
|  | NCS | Delta |  | 0.716 | 0.001 | 0.283 | 1.481 | 0.627 | 0.000 | 0.373 | 1.104 |
|  | WnoF | Delta | 0.005 | 0.937 | 0.020 | 0.043 | 1.325 | 0.953 | 0.019 | 0.028 | 0.949 |
|  |  | MC |  | 0.948 | 0.017 | 0.035 | 1.289 | 0.958 | 0.019 | 0.023 | 0.933 |
|  | Weibull | Delta |  | 0.881 | 0.071 | 0.047 | 1.606 | 0.945 | 0.031 | 0.024 | 0.880 |
|  |  | MC |  | 0.896 | 0.046 | 0.057 | 1.715 | 0.951 | 0.029 | 0.020 | 0.876 |
|  | PMS | Delta |  | 0.876 | 0.045 | 0.080 | 2.026 | 0.869 | 0.095 | 0.036 | 1.635 |
|  |  | MC |  | 0.949 | 0.011 | 0.039 | 1.819 | 0.933 | 0.047 | 0.021 | 1.515 |
|  | PTPRS | Delta |  | 0.936 | 0.036 | 0.028 | 1.705 | 0.946 | 0.037 | 0.017 | 1.268 |
|  | NCS | Delta |  | 0.845 | 0.004 | 0.151 | 1.189 | 0.794 | 0.001 | 0.205 | 0.878 |
|  | WnoF | Delta | 0.010 | 0.940 | 0.020 | 0.040 | 1.029 | 0.955 | 0.019 | 0.026 | 0.734 |
|  |  | MC |  | 0.947 | 0.019 | 0.034 | 1.011 | 0.960 | 0.017 | 0.023 | 0.726 |
|  | Weibull | Delta |  | 0.828 | 0.098 | 0.074 | 1.279 | 0.936 | 0.033 | 0.031 | 0.684 |
|  |  | MC |  | 0.874 | 0.057 | 0.069 | 1.457 | 0.941 | 0.033 | 0.026 | 0.684 |
|  | PMS | Delta |  | 0.895 | 0.062 | 0.042 | 1.474 | 0.647 | 0.340 | 0.013 | 1.040 |
|  |  | MC |  | 0.945 | 0.026 | 0.029 | 1.376 | 0.747 | 0.240 | 0.012 | 1.038 |
|  | PTPRS | Delta |  | 0.948 | 0.030 | 0.022 | 1.236 | 0.947 | 0.034 | 0.019 | 0.910 |
|  | NCS | Delta |  | 0.896 | 0.006 | 0.098 | 0.991 | 0.892 | 0.003 | 0.105 | 0.726 |
| 30% | WnoF | Delta | 0.002 | 0.915 | 0.019 | 0.066 | 1.947 | 0.931 | 0.014 | 0.055 | 1.408 |
|  |  | MC |  | 0.943 | 0.012 | 0.045 | 1.851 | 0.945 | 0.011 | 0.044 | 1.371 |
|  | Weibull | Delta |  | 0.917 | 0.037 | 0.046 | 1.837 | 0.847 | 0.130 | 0.023 | 1.879 |
|  |  | MC |  | 0.946 | 0.024 | 0.030 | 1.761 | 0.858 | 0.073 | 0.068 | 2.119 |
|  | PMS | Delta |  | 0.825 | 0.063 | 0.112 | 2.963 | 0.863 | 0.039 | 0.099 | 2.250 |
|  |  | MC |  | 0.945 | 0.010 | 0.045 | 2.523 | 0.934 | 0.013 | 0.053 | 1.997 |
|  | PTPRS | Delta |  | 0.907 | 0.060 | 0.033 | 2.554 | 0.933 | 0.052 | 0.015 | 1.935 |
|  | NCS | Delta |  | 0.793 | 0.004 | 0.203 | 1.769 | 0.742 | 0.002 | 0.256 | 1.320 |
|  | WnoF | Delta | 0.005 | 0.925 | 0.017 | 0.058 | 1.416 | 0.935 | 0.013 | 0.052 | 1.015 |
|  |  | MC |  | 0.944 | 0.011 | 0.045 | 1.365 | 0.944 | 0.011 | 0.045 | 0.994 |
|  | Weibull | Delta |  | 0.926 | 0.033 | 0.041 | 1.342 | 0.738 | 0.176 | 0.086 | 1.545 |
|  |  | MC |  | 0.944 | 0.027 | 0.029 | 1.316 | 0.762 | 0.124 | 0.114 | 1.773 |
|  | PMS | Delta |  | 0.871 | 0.068 | 0.062 | 2.036 | 0.891 | 0.069 | 0.040 | 1.484 |
|  |  | MC |  | 0.949 | 0.020 | 0.032 | 1.834 | 0.939 | 0.036 | 0.025 | 1.386 |
|  | PTPRS | Delta |  | 0.932 | 0.040 | 0.028 | 1.727 | 0.946 | 0.036 | 0.018 | 1.280 |
|  | NCS | Delta |  | 0.873 | 0.008 | 0.119 | 1.382 | 0.853 | 0.005 | 0.142 | 1.019 |
|  | WnoF | Delta | 0.010 | 0.930 | 0.018 | 0.052 | 1.093 | 0.940 | 0.014 | 0.046 | 0.780 |
|  |  | MC |  | 0.943 | 0.011 | 0.046 | 1.061 | 0.944 | 0.013 | 0.043 | 0.764 |
|  | Weibull | Delta |  | 0.930 | 0.035 | 0.035 | 1.037 | 0.641 | 0.209 | 0.150 | 1.277 |
|  |  | MC |  | 0.946 | 0.029 | 0.025 | 1.033 | 0.699 | 0.158 | 0.143 | 1.575 |
|  | PMS | Delta |  | 0.899 | 0.067 | 0.034 | 1.446 | 0.866 | 0.119 | 0.015 | 1.020 |
|  |  | MC |  | 0.946 | 0.026 | 0.029 | 1.373 | 0.905 | 0.077 | 0.017 | 0.987 |
|  | PTPRS | Delta |  | 0.943 | 0.032 | 0.025 | 1.254 | 0.953 | 0.027 | 0.020 | 0.918 |
|  | NCS | Delta |  | 0.924 | 0.010 | 0.066 | 1.124 | 0.909 | 0.011 | 0.080 | 0.823 |
| 60% | WnoF | Delta | 0.002 | 0.918 | 0.028 | 0.054 | 2.178 | 0.931 | 0.021 | 0.048 | 1.597 |
|  |  | MC |  | 0.953 | 0.014 | 0.033 | 2.063 | 0.958 | 0.011 | 0.031 | 1.556 |
|  | Weibull | Delta |  | 0.911 | 0.046 | 0.043 | 2.090 | 0.875 | 0.104 | 0.021 | 1.772 |
|  |  | MC |  | 0.952 | 0.021 | 0.027 | 1.940 | 0.918 | 0.059 | 0.023 | 1.995 |
|  | PMS | Delta |  | 0.806 | 0.114 | 0.080 | 2.976 | 0.856 | 0.089 | 0.055 | 2.275 |
|  |  | MC |  | 0.948 | 0.018 | 0.034 | 2.573 | 0.949 | 0.027 | 0.024 | 2.053 |
|  | PTPRS | Delta |  | 0.891 | 0.071 | 0.038 | 2.503 | 0.917 | 0.061 | 0.022 | 2.002 |
|  | NCS | Delta |  | 0.852 | 0.024 | 0.124 | 2.231 | 0.853 | 0.017 | 0.130 | 1.695 |
|  | WnoF | Delta | 0.005 | 0.927 | 0.024 | 0.049 | 1.565 | 0.941 | 0.017 | 0.042 | 1.132 |
|  |  | MC |  | 0.953 | 0.014 | 0.033 | 1.516 | 0.960 | 0.010 | 0.030 | 1.121 |
|  | Weibull | Delta |  | 0.923 | 0.036 | 0.041 | 1.507 | 0.810 | 0.158 | 0.031 | 1.260 |
|  |  | MC |  | 0.952 | 0.022 | 0.026 | 1.431 | 0.860 | 0.112 | 0.028 | 1.491 |
|  | PMS | Delta |  | 0.853 | 0.099 | 0.048 | 1.974 | 0.890 | 0.088 | 0.022 | 1.443 |
|  |  | MC |  | 0.946 | 0.022 | 0.032 | 1.853 | 0.942 | 0.037 | 0.021 | 1.395 |
|  | PTPRS | Delta |  | 0.915 | 0.050 | 0.035 | 1.709 | 0.936 | 0.044 | 0.020 | 1.315 |
|  | NCS | Delta |  | 0.890 | 0.029 | 0.081 | 1.668 | 0.911 | 0.018 | 0.071 | 1.241 |
|  | WnoF | Delta | 0.010 | 0.936 | 0.021 | 0.043 | 1.190 | 0.948 | 0.018 | 0.034 | 0.854 |
|  |  | MC |  | 0.958 | 0.015 | 0.027 | 1.172 | 0.964 | 0.009 | 0.027 | 0.857 |
|  | Weibull | Delta |  | 0.936 | 0.029 | 0.035 | 1.148 | 0.775 | 0.193 | 0.032 | 0.966 |
|  |  | MC |  | 0.950 | 0.024 | 0.026 | 1.112 | 0.809 | 0.164 | 0.027 | 1.107 |
|  | PMS | Delta |  | 0.908 | 0.061 | 0.030 | 1.367 | 0.912 | 0.072 | 0.015 | 0.969 |
|  |  | MC |  | 0.948 | 0.029 | 0.023 | 1.361 | 0.937 | 0.047 | 0.016 | 0.970 |
|  | PTPRS | Delta |  | 0.931 | 0.038 | 0.031 | 1.250 | 0.941 | 0.037 | 0.022 | 0.939 |
|  | NCS | Delta |  | 0.906 | 0.036 | 0.058 | 1.298 | 0.933 | 0.028 | 0.039 | 0.951 |
| 90% | WnoF | Delta | 0.002 | 0.867 | 0.079 | 0.054 | 2.625 | 0.908 | 0.062 | 0.030 | 1.943 |
|  |  | MC |  | 0.933 | 0.034 | 0.033 | 2.473 | 0.943 | 0.035 | 0.022 | 1.857 |
|  | Weibull | Delta |  | 0.867 | 0.081 | 0.052 | 2.600 | 0.907 | 0.064 | 0.029 | 1.926 |
|  |  | MC |  | 0.939 | 0.030 | 0.031 | 2.505 | 0.948 | 0.033 | 0.019 | 1.867 |
|  | PMS | Delta |  | 0.803 | 0.126 | 0.072 | 2.917 | 0.866 | 0.096 | 0.038 | 2.249 |
|  |  | MC |  | 0.945 | 0.025 | 0.030 | 2.813 | 0.947 | 0.030 | 0.023 | 2.170 |
|  | PTPRS | Delta |  | 0.855 | 0.088 | 0.057 | 2.624 | 0.903 | 0.066 | 0.031 | 1.973 |
|  | NCS | Delta |  | 0.801 | 0.123 | 0.076 | 2.739 | 0.850 | 0.094 | 0.056 | 2.114 |
|  | WnoF | Delta | 0.005 | 0.895 | 0.062 | 0.043 | 1.835 | 0.925 | 0.055 | 0.020 | 1.322 |
|  |  | MC |  | 0.939 | 0.030 | 0.031 | 1.805 | 0.947 | 0.032 | 0.021 | 1.301 |
|  | Weibull | Delta |  | 0.895 | 0.062 | 0.043 | 1.821 | 0.926 | 0.055 | 0.019 | 1.313 |
|  |  | MC |  | 0.946 | 0.026 | 0.028 | 1.850 | 0.952 | 0.030 | 0.018 | 1.314 |
|  | PMS | Delta |  | 0.879 | 0.080 | 0.041 | 1.985 | 0.909 | 0.070 | 0.020 | 1.442 |
|  |  | MC |  | 0.946 | 0.025 | 0.029 | 2.133 | 0.945 | 0.035 | 0.020 | 1.507 |
|  | PTPRS | Delta |  | 0.886 | 0.067 | 0.047 | 1.842 | 0.921 | 0.054 | 0.025 | 1.334 |
|  | NCS | Delta |  | 0.866 | 0.085 | 0.049 | 1.938 | 0.897 | 0.074 | 0.029 | 1.427 |
|  | WnoF | Delta | 0.010 | 0.923 | 0.039 | 0.038 | 1.370 | 0.939 | 0.039 | 0.022 | 0.964 |
|  |  | MC |  | 0.946 | 0.025 | 0.029 | 1.405 | 0.951 | 0.030 | 0.019 | 0.979 |
|  | Weibull | Delta |  | 0.920 | 0.038 | 0.042 | 1.361 | 0.939 | 0.039 | 0.022 | 0.960 |
|  |  | MC |  | 0.940 | 0.028 | 0.032 | 1.457 | 0.950 | 0.031 | 0.019 | 0.987 |
|  | PMS | Delta |  | 0.914 | 0.051 | 0.034 | 1.431 | 0.939 | 0.039 | 0.022 | 1.022 |
|  |  | MC |  | 0.944 | 0.025 | 0.031 | 1.798 | 0.952 | 0.024 | 0.024 | 1.197 |
|  | PTPRS | Delta |  | 0.916 | 0.042 | 0.042 | 1.368 | 0.939 | 0.041 | 0.020 | 0.978 |
|  | NCS | Delta |  | 0.918 | 0.046 | 0.036 | 1.430 | 0.937 | 0.040 | 0.023 | 1.021 |

Note: Metrics are calculated based on the logarithms of TTB estimates. The number of MC samples is 2,000. ARR: absolute relative risk; CP: coverage probability; OLLP: out-of-lower-limit probability; OULP: out-of-upper-limit probability; MAE: mean absolute error; RMSE: root mean squared error; MC: Monte Carlo; NCS: natural cubic spline; PMS: penalized M-spline; PTPRS: penalized thin plate regression spline; SPRINT: Systolic Blood Pressure Intervention Trial; TTB: time to benefit; WF: Weibull model with shared frailty; WnoF: Weibull model without shared frailty

**Table S8.** TTB point estimation results for simulation scenario II with the Gamma frailty and 1,000 replicates.

|  |  |  |  | N=5,000 | | | | N=10,000 | | | |
| --- | --- | --- | --- | --- | --- | --- | --- | --- | --- | --- | --- |
| Cesnoring | Model | Method | ARR | Bias | MAE | RMSE | Time | Bias | MAE | RMSE | Time |
| 0% | WnoF | Delta | 0.002 | -0.323 | 0.455 | 0.561 | 1min | -0.307 | 0.366 | 0.446 | 1min |
|  |  | MC |  | -0.334 | 0.460 | 0.566 | 1hrs32min | -0.315 | 0.371 | 0.451 | 1hrs33min |
|  | WF | Delta |  | -0.274 | 0.411 | 0.502 | 26min | -0.266 | 0.327 | 0.400 | 1hrs51min |
|  |  | MC |  | -0.296 | 0.422 | 0.514 | 2hrs23min | -0.283 | 0.337 | 0.411 | 3hrs50min |
|  | PMS | Delta |  | -0.203 | 0.597 | 0.771 | 3hrs28min | -0.164 | 0.439 | 0.571 | 9hrs45min |
|  |  | MC |  | -0.167 | 0.571 | 0.740 | 6hrs14min | -0.134 | 0.421 | 0.547 | 12hrs32min |
|  | PTPRS | Delta |  | -0.233 | 0.508 | 0.660 | 17hrs50min | -0.208 | 0.392 | 0.510 | 19hrs21min |
|  | NCS | Delta |  | -0.249 | 0.549 | 0.665 | 5hrs58min | -0.245 | 0.431 | 0.525 | 5hrs19min |
|  | WnoF | Delta | 0.005 | -0.181 | 0.310 | 0.382 |  | -0.171 | 0.237 | 0.292 |  |
|  |  | MC |  | -0.189 | 0.313 | 0.386 |  | -0.177 | 0.240 | 0.295 |  |
|  | WF | Delta |  | -0.140 | 0.276 | 0.339 |  | -0.135 | 0.207 | 0.257 |  |
|  |  | MC |  | -0.156 | 0.282 | 0.346 |  | -0.147 | 0.214 | 0.264 |  |
|  | PMS | Delta |  | -0.054 | 0.379 | 0.489 |  | -0.030 | 0.260 | 0.334 |  |
|  |  | MC |  | -0.034 | 0.371 | 0.475 |  | -0.016 | 0.256 | 0.326 |  |
|  | PTPRS | Delta |  | -0.094 | 0.330 | 0.423 |  | -0.076 | 0.240 | 0.306 |  |
|  | NCS | Delta |  | -0.083 | 0.368 | 0.452 |  | -0.075 | 0.268 | 0.332 |  |
|  | WnoF | Delta | 0.010 | -0.102 | 0.226 | 0.280 |  | -0.095 | 0.166 | 0.205 |  |
|  |  | MC |  | -0.105 | 0.227 | 0.282 |  | -0.098 | 0.167 | 0.206 |  |
|  | WF | Delta |  | -0.072 | 0.204 | 0.252 |  | -0.069 | 0.147 | 0.183 |  |
|  |  | MC |  | -0.086 | 0.207 | 0.256 |  | -0.079 | 0.151 | 0.187 |  |
|  | PMS | Delta |  | -0.007 | 0.262 | 0.335 |  | 0.006 | 0.176 | 0.224 |  |
|  |  | MC |  | 0.005 | 0.261 | 0.331 |  | 0.014 | 0.176 | 0.223 |  |
|  | PTPRS | Delta |  | -0.039 | 0.235 | 0.299 |  | -0.027 | 0.167 | 0.211 |  |
|  | NCS | Delta |  | -0.016 | 0.269 | 0.333 |  | -0.006 | 0.189 | 0.236 |  |
| 30% | WnoF | Delta | 0.002 | -0.370 | 0.505 | 0.617 | 1min | -0.366 | 0.417 | 0.509 | 2min |
|  |  | MC |  | -0.385 | 0.512 | 0.625 | 1hrs34min | -0.377 | 0.424 | 0.516 | 1hrs36min |
|  | WF | Delta |  | -0.261 | 0.434 | 0.529 | 22min | -0.263 | 0.338 | 0.421 | 1hrs43min |
|  |  | MC |  | -0.282 | 0.443 | 0.539 | 2hrs20min | -0.278 | 0.346 | 0.430 | 3hrs44min |
|  | PMS | Delta |  | -0.211 | 0.619 | 0.791 | 2hrs14min | -0.164 | 0.445 | 0.580 | 5hrs11min |
|  |  | MC |  | -0.180 | 0.594 | 0.761 | 5hrs2min | -0.138 | 0.430 | 0.559 | 8hrs4min |
|  | PTPRS | Delta |  | -0.234 | 0.526 | 0.680 | 17hrs1min | -0.207 | 0.403 | 0.525 | 20hrs32min |
|  | NCS | Delta |  | -0.177 | 0.552 | 0.667 | 5hrs5min | -0.186 | 0.430 | 0.528 | 5hrs24min |
|  | WnoF | Delta | 0.005 | -0.209 | 0.340 | 0.417 |  | -0.208 | 0.267 | 0.329 |  |
|  |  | MC |  | -0.219 | 0.344 | 0.421 |  | -0.215 | 0.271 | 0.333 |  |
|  | WF | Delta |  | -0.129 | 0.297 | 0.362 |  | -0.133 | 0.219 | 0.273 |  |
|  |  | MC |  | -0.144 | 0.300 | 0.366 |  | -0.144 | 0.222 | 0.277 |  |
|  | PMS | Delta |  | -0.054 | 0.389 | 0.498 |  | -0.034 | 0.268 | 0.344 |  |
|  |  | MC |  | -0.033 | 0.383 | 0.487 |  | -0.020 | 0.265 | 0.339 |  |
|  | PTPRS | Delta |  | -0.090 | 0.341 | 0.433 |  | -0.075 | 0.247 | 0.314 |  |
|  | NCS | Delta |  | -0.044 | 0.373 | 0.458 |  | -0.045 | 0.275 | 0.340 |  |
|  | WnoF | Delta | 0.010 | -0.121 | 0.247 | 0.304 |  | -0.120 | 0.185 | 0.229 |  |
|  |  | MC |  | -0.127 | 0.249 | 0.306 |  | -0.124 | 0.186 | 0.231 |  |
|  | WF | Delta |  | -0.065 | 0.221 | 0.270 |  | -0.067 | 0.158 | 0.196 |  |
|  |  | MC |  | -0.077 | 0.223 | 0.273 |  | -0.077 | 0.160 | 0.199 |  |
|  | PMS | Delta |  | -0.003 | 0.268 | 0.339 |  | 0.000 | 0.185 | 0.233 |  |
|  |  | MC |  | 0.012 | 0.267 | 0.335 |  | 0.010 | 0.186 | 0.233 |  |
|  | PTPRS | Delta |  | -0.034 | 0.242 | 0.306 |  | -0.027 | 0.172 | 0.217 |  |
|  | NCS | MC |  | 0.002 | 0.270 | 0.335 |  | 0.005 | 0.192 | 0.239 |  |
| 60% | WnoF | Delta | 0.002 | -0.333 | 0.526 | 0.645 | 1min | -0.329 | 0.416 | 0.512 | 1min |
|  |  | Delta |  | -0.344 | 0.528 | 0.648 | 1hrs34min | -0.338 | 0.420 | 0.516 | 1hrs34min |
|  | WF | Delta |  | -0.237 | 0.476 | 0.585 | 23min | -0.233 | 0.356 | 0.446 | 1hrs35min |
|  |  | MC |  | -0.266 | 0.487 | 0.598 | 2hrs23min | -0.256 | 0.367 | 0.458 | 3hrs37min |
|  | PMS | Delta |  | -0.192 | 0.607 | 0.780 | 1hrs59min | -0.149 | 0.441 | 0.575 | 2hrs8min |
|  |  | MC |  | -0.161 | 0.589 | 0.757 | 4hrs51min | -0.123 | 0.431 | 0.560 | 5hrs2min |
|  | PTPRS | Delta |  | -0.230 | 0.539 | 0.701 | 21hrs49min | -0.199 | 0.406 | 0.542 | 20hrs57min |
|  | NCS | MC |  | -0.135 | 0.548 | 0.688 | 5hrs16min | -0.133 | 0.417 | 0.525 | 5hrs26min |
|  | WnoF | Delta | 0.005 | -0.175 | 0.354 | 0.434 |  | -0.174 | 0.264 | 0.328 |  |
|  |  | Delta |  | -0.183 | 0.355 | 0.436 |  | -0.180 | 0.266 | 0.330 |  |
|  | WF | Delta |  | -0.112 | 0.327 | 0.403 |  | -0.112 | 0.234 | 0.293 |  |
|  |  | MC |  | -0.132 | 0.333 | 0.410 |  | -0.127 | 0.240 | 0.299 |  |
|  | PMS | Delta |  | -0.047 | 0.382 | 0.492 |  | -0.025 | 0.261 | 0.336 |  |
|  |  | MC |  | -0.025 | 0.372 | 0.481 |  | -0.008 | 0.255 | 0.330 |  |
|  | PTPRS | Delta |  | -0.085 | 0.346 | 0.439 |  | -0.065 | 0.245 | 0.313 |  |
|  | NCS | MC |  | -0.038 | 0.372 | 0.472 |  | -0.031 | 0.263 | 0.333 |  |
|  | WnoF | Delta | 0.010 | -0.093 | 0.256 | 0.317 |  | -0.092 | 0.184 | 0.229 |  |
|  |  | Delta |  | -0.099 | 0.257 | 0.317 |  | -0.097 | 0.185 | 0.230 |  |
|  | WF | Delta |  | -0.053 | 0.242 | 0.300 |  | -0.053 | 0.169 | 0.211 |  |
|  |  | MC |  | -0.068 | 0.245 | 0.303 |  | -0.063 | 0.171 | 0.214 |  |
|  | PMS | Delta |  | -0.004 | 0.261 | 0.335 |  | 0.000 | 0.179 | 0.227 |  |
|  |  | MC |  | 0.011 | 0.258 | 0.330 |  | 0.010 | 0.178 | 0.225 |  |
|  | PTPRS | Delta |  | -0.032 | 0.245 | 0.310 |  | -0.022 | 0.172 | 0.217 |  |
|  | NCS | MC |  | -0.006 | 0.263 | 0.337 |  | 0.001 | 0.181 | 0.230 |  |
| 90% | WnoF | Delta | 0.002 | -0.253 | 0.560 | 0.712 | 1min | -0.228 | 0.416 | 0.535 | 2min |
|  |  | Delta |  | -0.251 | 0.551 | 0.702 | 1hrs40min | -0.225 | 0.412 | 0.530 | 1hrs35min |
|  | WF | Delta |  | -0.245 | 0.554 | 0.704 | 19min | -0.221 | 0.411 | 0.529 | 1hrs32min |
|  |  | MC |  | -0.273 | 0.568 | 0.721 | 3hrs6min | -0.228 | 0.413 | 0.534 | 3hrs44min |
|  | PMS | Delta |  | -0.194 | 0.626 | 0.801 | 1hrs33min | -0.132 | 0.445 | 0.586 | 1hrs14min |
|  |  | MC |  | -0.177 | 0.601 | 0.774 | 4hrs10min | -0.115 | 0.432 | 0.568 | 4hrs21min |
|  | PTPRS | Delta |  | -0.225 | 0.579 | 0.757 | 29hrs20min | -0.179 | 0.426 | 0.566 | 45hrs33min |
|  | NCS | MC |  | -0.227 | 0.634 | 0.852 | 6hrs47min | -0.129 | 0.441 | 0.629 | 6hrs18min |
|  | WnoF | Delta | 0.005 | -0.109 | 0.382 | 0.482 |  | -0.096 | 0.272 | 0.347 |  |
|  |  | Delta |  | -0.113 | 0.377 | 0.476 |  | -0.098 | 0.270 | 0.344 |  |
|  | WF | Delta |  | -0.105 | 0.381 | 0.481 |  | -0.094 | 0.270 | 0.344 |  |
|  |  | MC |  | -0.122 | 0.385 | 0.486 |  | -0.100 | 0.269 | 0.344 |  |
|  | PMS | Delta |  | -0.035 | 0.394 | 0.510 |  | -0.015 | 0.271 | 0.351 |  |
|  |  | MC |  | -0.030 | 0.388 | 0.500 |  | -0.006 | 0.271 | 0.349 |  |
|  | PTPRS | Delta |  | -0.077 | 0.383 | 0.489 |  | -0.054 | 0.262 | 0.336 |  |
|  | NCS | MC |  | -0.053 | 0.398 | 0.525 |  | -0.023 | 0.272 | 0.351 |  |
|  | WnoF | Delta | 0.010 | -0.039 | 0.283 | 0.357 |  | -0.032 | 0.196 | 0.249 |  |
|  |  | Delta |  | -0.046 | 0.279 | 0.353 |  | -0.036 | 0.194 | 0.247 |  |
|  | WF | Delta |  | -0.039 | 0.282 | 0.356 |  | -0.033 | 0.195 | 0.248 |  |
|  |  | MC |  | -0.049 | 0.284 | 0.358 |  | -0.039 | 0.196 | 0.249 |  |
|  | PMS | Delta |  | 0.010 | 0.282 | 0.363 |  | 0.009 | 0.201 | 0.251 |  |
|  |  | MC |  | 0.015 | 0.278 | 0.356 |  | 0.018 | 0.200 | 0.251 |  |
|  | PTPRS | Delta |  | -0.020 | 0.279 | 0.352 |  | -0.013 | 0.193 | 0.244 |  |
|  | NCS | MC |  | -0.012 | 0.299 | 0.377 |  | -0.005 | 0.214 | 0.267 |  |

Note: Metrics are calculated based on the logarithms of TTB estimates. The number of MC samples is 2,000. For MC method, the estimates are the median of the MC samples. Time is for all ARR values for each Model. ARR: absolute relative risk; MAE: mean absolute error; RMSE: root mean squared error; MC: Monte Carlo; NCS: natural cubic spline; PMS: penalized M-spline; PTPRS: penalized thin plate regression spline; SPRINT: Systolic Blood Pressure Intervention Trial; TTB: time to benefit; WF: Weibull model with shared frailty; WnoF: Weibull model without shared frailty

**Table S9.** TTB interval estimation results for simulation scenario II with the Gamma frailty and 1,000 replicates.

|  |  |  |  | N=5,000 | | | | N=10,000 | | | |
| --- | --- | --- | --- | --- | --- | --- | --- | --- | --- | --- | --- |
| Cesnoring | Model | Method | ARR | CP | OLLP | OULP | length | CP | OLLP | OULP | length |
| 0% | WnoF | Delta | 0.002 | 0.886 | 0.005 | 0.109 | 1.795 | 0.855 | 0.002 | 0.143 | 1.291 |
|  |  | MC |  | 0.908 | 0.001 | 0.091 | 1.738 | 0.873 | 0.002 | 0.125 | 1.272 |
|  | WF | Delta |  | 0.886 | 0.005 | 0.109 | 1.645 | 0.855 | 0.003 | 0.142 | 1.183 |
|  |  | MC |  | 0.898 | 0.000 | 0.102 | 1.594 | 0.851 | 0.002 | 0.147 | 1.162 |
|  | PMS | Delta |  | 0.845 | 0.076 | 0.079 | 2.835 | 0.901 | 0.055 | 0.044 | 2.165 |
|  |  | MC |  | 0.935 | 0.028 | 0.037 | 2.491 | 0.947 | 0.025 | 0.028 | 1.956 |
|  | PTPRS | Delta |  | 0.914 | 0.037 | 0.049 | 2.564 | 0.942 | 0.026 | 0.032 | 1.970 |
|  | NCS | Delta |  | 0.829 | 0.042 | 0.129 | 2.428 | 0.871 | 0.026 | 0.103 | 1.888 |
|  | WnoF | Delta | 0.005 | 0.900 | 0.009 | 0.091 | 1.321 | 0.894 | 0.003 | 0.103 | 0.944 |
|  |  | MC |  | 0.930 | 0.004 | 0.066 | 1.305 | 0.913 | 0.003 | 0.084 | 0.945 |
|  | Weibull | Delta |  | 0.911 | 0.006 | 0.083 | 1.208 | 0.902 | 0.006 | 0.092 | 0.864 |
|  |  | MC |  | 0.924 | 0.003 | 0.073 | 1.188 | 0.900 | 0.004 | 0.096 | 0.853 |
|  | PMS | Delta |  | 0.889 | 0.069 | 0.042 | 1.854 | 0.926 | 0.055 | 0.019 | 1.345 |
|  |  | MC |  | 0.939 | 0.032 | 0.029 | 1.758 | 0.951 | 0.031 | 0.018 | 1.299 |
|  | PTPRS | Delta |  | 0.932 | 0.033 | 0.035 | 1.685 | 0.953 | 0.023 | 0.024 | 1.247 |
|  | NCS | Delta |  | 0.878 | 0.051 | 0.071 | 1.748 | 0.916 | 0.032 | 0.052 | 1.314 |
|  | WnoF | Delta | 0.010 | 0.920 | 0.012 | 0.068 | 1.026 | 0.925 | 0.007 | 0.068 | 0.730 |
|  |  | MC |  | 0.941 | 0.009 | 0.050 | 1.019 | 0.938 | 0.006 | 0.056 | 0.732 |
|  | Weibull | Delta |  | 0.935 | 0.014 | 0.051 | 0.942 | 0.924 | 0.010 | 0.066 | 0.672 |
|  |  | MC |  | 0.944 | 0.006 | 0.050 | 0.937 | 0.927 | 0.006 | 0.067 | 0.669 |
|  | PMS | Delta |  | 0.917 | 0.054 | 0.029 | 1.281 | 0.947 | 0.041 | 0.012 | 0.908 |
|  |  | MC |  | 0.944 | 0.031 | 0.025 | 1.274 | 0.957 | 0.029 | 0.014 | 0.906 |
|  | PTPRS | Delta |  | 0.947 | 0.029 | 0.024 | 1.205 | 0.965 | 0.017 | 0.018 | 0.879 |
|  | NCS | Delta |  | 0.904 | 0.052 | 0.044 | 1.303 | 0.935 | 0.041 | 0.024 | 0.955 |
| 30% | WnoF | Delta | 0.002 | 0.858 | 0.007 | 0.135 | 1.929 | 0.828 | 0.002 | 0.170 | 1.397 |
|  |  | MC |  | 0.879 | 0.002 | 0.119 | 1.809 | 0.830 | 0.002 | 0.168 | 1.330 |
|  | Weibull | Delta |  | 0.891 | 0.009 | 0.100 | 1.799 | 0.879 | 0.003 | 0.118 | 1.302 |
|  |  | MC |  | 0.902 | 0.001 | 0.097 | 1.712 | 0.867 | 0.002 | 0.131 | 1.260 |
|  | PMS | Delta |  | 0.844 | 0.081 | 0.075 | 2.926 | 0.900 | 0.058 | 0.042 | 2.176 |
|  |  | MC |  | 0.942 | 0.024 | 0.034 | 2.587 | 0.948 | 0.024 | 0.028 | 2.016 |
|  | PTPRS | Delta |  | 0.909 | 0.041 | 0.050 | 2.617 | 0.943 | 0.027 | 0.030 | 2.016 |
|  | NCS | Delta |  | 0.845 | 0.063 | 0.092 | 2.535 | 0.880 | 0.042 | 0.078 | 1.980 |
|  | WnoF | Delta | 0.005 | 0.895 | 0.008 | 0.097 | 1.411 | 0.875 | 0.002 | 0.123 | 1.013 |
|  |  | MC |  | 0.918 | 0.006 | 0.076 | 1.361 | 0.886 | 0.002 | 0.112 | 0.985 |
|  | Weibull | Delta |  | 0.914 | 0.013 | 0.073 | 1.319 | 0.906 | 0.007 | 0.087 | 0.947 |
|  |  | MC |  | 0.926 | 0.003 | 0.070 | 1.274 | 0.906 | 0.002 | 0.092 | 0.924 |
|  | PMS | Delta |  | 0.894 | 0.067 | 0.039 | 1.914 | 0.925 | 0.056 | 0.019 | 1.361 |
|  |  | MC |  | 0.941 | 0.028 | 0.031 | 1.824 | 0.950 | 0.027 | 0.023 | 1.334 |
|  | PTPRS | Delta |  | 0.936 | 0.033 | 0.031 | 1.729 | 0.952 | 0.024 | 0.024 | 1.271 |
|  | NCS | Delta |  | 0.883 | 0.068 | 0.049 | 1.801 | 0.910 | 0.053 | 0.037 | 1.354 |
|  | WnoF | Delta | 0.010 | 0.924 | 0.009 | 0.067 | 1.093 | 0.911 | 0.004 | 0.085 | 0.780 |
|  |  | MC |  | 0.940 | 0.008 | 0.052 | 1.075 | 0.924 | 0.004 | 0.072 | 0.774 |
|  | Weibull | Delta |  | 0.931 | 0.015 | 0.054 | 1.024 | 0.924 | 0.010 | 0.066 | 0.733 |
|  |  | MC |  | 0.941 | 0.005 | 0.054 | 1.001 | 0.924 | 0.007 | 0.069 | 0.720 |
|  | PMS | Delta |  | 0.925 | 0.051 | 0.024 | 1.330 | 0.932 | 0.051 | 0.017 | 0.940 |
|  |  | MC |  | 0.950 | 0.030 | 0.020 | 1.318 | 0.957 | 0.029 | 0.014 | 0.938 |
|  | PTPRS | Delta |  | 0.940 | 0.032 | 0.028 | 1.237 | 0.960 | 0.020 | 0.020 | 0.900 |
|  | NCS | Delta |  | 0.900 | 0.066 | 0.034 | 1.320 | 0.925 | 0.057 | 0.018 | 0.964 |
| 60% | WnoF | Delta | 0.002 | 0.876 | 0.013 | 0.111 | 2.157 | 0.871 | 0.004 | 0.125 | 1.580 |
|  |  | MC |  | 0.897 | 0.007 | 0.096 | 1.970 | 0.870 | 0.002 | 0.128 | 1.477 |
|  | Weibull | Delta |  | 0.895 | 0.025 | 0.081 | 2.065 | 0.908 | 0.010 | 0.082 | 1.511 |
|  |  | MC |  | 0.916 | 0.007 | 0.077 | 1.958 | 0.902 | 0.003 | 0.095 | 1.456 |
|  | PMS | Delta |  | 0.841 | 0.085 | 0.074 | 2.911 | 0.893 | 0.066 | 0.041 | 2.188 |
|  |  | MC |  | 0.939 | 0.026 | 0.035 | 2.571 | 0.954 | 0.022 | 0.024 | 2.051 |
|  | PTPRS | Delta |  | 0.890 | 0.044 | 0.066 | 2.553 | 0.935 | 0.030 | 0.035 | 2.056 |
|  | NCS | Delta |  | 0.830 | 0.091 | 0.079 | 2.562 | 0.881 | 0.067 | 0.052 | 1.990 |
|  | WnoF | Delta | 0.005 | 0.905 | 0.017 | 0.078 | 1.552 | 0.907 | 0.006 | 0.087 | 1.123 |
|  |  | MC |  | 0.924 | 0.010 | 0.066 | 1.450 | 0.914 | 0.005 | 0.081 | 1.067 |
|  | Weibull | Delta |  | 0.911 | 0.027 | 0.062 | 1.492 | 0.932 | 0.011 | 0.057 | 1.080 |
|  |  | MC |  | 0.928 | 0.012 | 0.059 | 1.445 | 0.931 | 0.006 | 0.063 | 1.063 |
|  | PMS | Delta |  | 0.890 | 0.071 | 0.039 | 1.871 | 0.928 | 0.053 | 0.019 | 1.339 |
|  |  | MC |  | 0.945 | 0.029 | 0.026 | 1.807 | 0.955 | 0.027 | 0.018 | 1.332 |
|  | PTPRS | Delta |  | 0.924 | 0.037 | 0.039 | 1.713 | 0.953 | 0.027 | 0.020 | 1.275 |
|  | NCS | Delta |  | 0.877 | 0.074 | 0.049 | 1.786 | 0.918 | 0.055 | 0.027 | 1.330 |
|  | WnoF | Delta | 0.010 | 0.926 | 0.017 | 0.057 | 1.179 | 0.933 | 0.009 | 0.058 | 0.846 |
|  |  | MC |  | 0.931 | 0.015 | 0.054 | 1.118 | 0.930 | 0.009 | 0.061 | 0.811 |
|  | Weibull | Delta |  | 0.925 | 0.027 | 0.048 | 1.136 | 0.944 | 0.014 | 0.042 | 0.817 |
|  |  | MC |  | 0.938 | 0.015 | 0.047 | 1.118 | 0.943 | 0.010 | 0.047 | 0.816 |
|  | PMS | Delta |  | 0.920 | 0.054 | 0.026 | 1.303 | 0.933 | 0.047 | 0.020 | 0.911 |
|  |  | MC |  | 0.946 | 0.029 | 0.025 | 1.313 | 0.955 | 0.029 | 0.016 | 0.935 |
|  | PTPRS | Delta |  | 0.937 | 0.031 | 0.032 | 1.221 | 0.959 | 0.022 | 0.019 | 0.902 |
|  | NCS | Delta |  | 0.910 | 0.060 | 0.030 | 1.295 | 0.936 | 0.046 | 0.018 | 0.934 |
| 90% | WnoF | Delta | 0.002 | 0.907 | 0.036 | 0.057 | 2.668 | 0.917 | 0.026 | 0.057 | 1.941 |
|  |  | MC |  | 0.938 | 0.017 | 0.045 | 2.476 | 0.928 | 0.013 | 0.059 | 1.841 |
|  | Weibull | Delta |  | 0.910 | 0.036 | 0.054 | 2.662 | 0.916 | 0.026 | 0.058 | 1.920 |
|  |  | MC |  | 0.933 | 0.016 | 0.051 | 2.514 | 0.924 | 0.008 | 0.068 | 1.858 |
|  | PMS | Delta |  | 0.831 | 0.088 | 0.081 | 3.092 | 0.893 | 0.069 | 0.038 | 2.211 |
|  |  | MC |  | 0.938 | 0.023 | 0.039 | 2.745 | 0.952 | 0.020 | 0.028 | 2.168 |
|  | PTPRS | Delta |  | 0.888 | 0.046 | 0.066 | 2.739 | 0.908 | 0.039 | 0.053 | 2.005 |
|  | NCS | Delta |  | 0.742 | 0.137 | 0.121 | 2.274 | 0.824 | 0.111 | 0.065 | 1.935 |
|  | WnoF | Delta | 0.005 | 0.926 | 0.033 | 0.041 | 1.855 | 0.930 | 0.025 | 0.045 | 1.332 |
|  |  | MC |  | 0.938 | 0.020 | 0.042 | 1.819 | 0.935 | 0.018 | 0.047 | 1.301 |
|  | Weibull | Delta |  | 0.924 | 0.035 | 0.041 | 1.889 | 0.931 | 0.025 | 0.044 | 1.320 |
|  |  | MC |  | 0.942 | 0.016 | 0.042 | 1.851 | 0.940 | 0.012 | 0.048 | 1.325 |
|  | PMS | Delta |  | 0.885 | 0.074 | 0.041 | 2.104 | 0.920 | 0.062 | 0.018 | 1.400 |
|  |  | MC |  | 0.946 | 0.026 | 0.028 | 2.014 | 0.950 | 0.028 | 0.022 | 1.456 |
|  | PTPRS | Delta |  | 0.917 | 0.041 | 0.042 | 1.871 | 0.938 | 0.036 | 0.026 | 1.315 |
|  | NCS | Delta |  | 0.808 | 0.127 | 0.065 | 1.619 | 0.856 | 0.100 | 0.044 | 1.419 |
|  | WnoF | Delta | 0.010 | 0.935 | 0.028 | 0.037 | 1.387 | 0.945 | 0.027 | 0.028 | 0.979 |
|  |  | MC |  | 0.937 | 0.025 | 0.038 | 1.417 | 0.943 | 0.024 | 0.033 | 0.983 |
|  | Weibull | Delta |  | 0.935 | 0.029 | 0.036 | 1.451 | 0.944 | 0.027 | 0.029 | 0.973 |
|  |  | MC |  | 0.949 | 0.013 | 0.038 | 1.469 | 0.949 | 0.017 | 0.034 | 1.004 |
|  | PMS | Delta |  | 0.915 | 0.055 | 0.030 | 2.670 | 0.924 | 0.050 | 0.026 | 1.014 |
|  |  | MC |  | 0.948 | 0.027 | 0.025 | 1.599 | 0.948 | 0.032 | 0.020 | 1.087 |
|  | PTPRS | Delta |  | 0.933 | 0.032 | 0.035 | 1.377 | 0.945 | 0.030 | 0.025 | 0.971 |
|  | NCS | Delta |  | 0.808 | 0.110 | 0.082 | 1.259 | 0.871 | 0.076 | 0.053 | 1.021 |

Note: Metrics are calculated based on the logarithms of TTB estimates. The number of MC samples is 2,000. ARR: absolute relative risk; CP: coverage probability; OLLP: out-of-lower-limit probability; OULP: out-of-upper-limit probability; MAE: mean absolute error; RMSE: root mean squared error; MC: Monte Carlo; NCS: natural cubic spline; PMS: penalized M-spline; PTPRS: penalized thin plate regression spline; SPRINT: Systolic Blood Pressure Intervention Trial; TTB: time to benefit; WF: Weibull model with shared frailty; WnoF: Weibull model without shared frailty

**Table S10.** TTB point estimation results for simulation scenario II with the Gamma frailty and 100 replicates.

|  |  |  |  | N=5,000 | | | | N=10,000 | | | |
| --- | --- | --- | --- | --- | --- | --- | --- | --- | --- | --- | --- |
| Cesnoring | Model | Method | ARR | Bias | MAE | RMSE | Time | Bias | MAE | RMSE | Time |
| 0% | WnoF | Delta | 0.002 | -0.390 | 0.512 | 0.601 | <1min | -0.305 | 0.346 | 0.424 | <1min |
|  |  | MC |  | -0.401 | 0.518 | 0.607 | 3min | -0.313 | 0.351 | 0.430 | 3min |
|  | WF | Delta |  | -0.333 | 0.461 | 0.542 | 3min | -0.261 | 0.299 | 0.374 | 5min |
|  |  | MC |  | -0.354 | 0.474 | 0.555 | 14min | -0.278 | 0.311 | 0.386 | 17min |
|  | PMS | Delta |  | -0.175 | 0.599 | 0.768 | 20min | -0.127 | 0.413 | 0.547 | 1hrs60min |
|  |  | MC |  | -0.140 | 0.572 | 0.737 | 1hrs36min | -0.099 | 0.397 | 0.527 | 1hrs15min |
|  | PTPRS | Delta |  | -0.230 | 0.515 | 0.676 | 2hrs37min | -0.194 | 0.377 | 0.496 | 2hrs54min |
|  |  | MC |  | -0.237 | 0.516 | 0.678 | 65hrs20min | -0.202 | 0.382 | 0.503 | 69hrs52min |
|  | NCS | Delta |  | -0.230 | 0.605 | 0.719 | 29min | -0.236 | 0.421 | 0.518 | 1hrs31min |
|  |  | MC |  | -0.220 | 0.597 | 0.709 | 22hrs17min | -0.227 | 0.415 | 0.510 | 23hrs33min |
|  | WnoF | Delta | 0.005 | -0.230 | 0.345 | 0.407 |  | -0.173 | 0.221 | 0.274 |  |
|  |  | MC |  | -0.238 | 0.349 | 0.411 |  | -0.179 | 0.224 | 0.277 |  |
|  | Weibull | Delta |  | -0.182 | 0.306 | 0.363 |  | -0.134 | 0.185 | 0.235 |  |
|  |  | MC |  | -0.199 | 0.314 | 0.371 |  | -0.147 | 0.191 | 0.242 |  |
|  | PMS | Delta |  | -0.029 | 0.404 | 0.506 |  | -0.018 | 0.255 | 0.330 |  |
|  |  | MC |  | -0.010 | 0.395 | 0.494 |  | -0.006 | 0.252 | 0.323 |  |
|  | PTPRS | Delta |  | -0.100 | 0.346 | 0.448 |  | -0.070 | 0.230 | 0.297 |  |
|  |  | MC |  | -0.106 | 0.347 | 0.450 |  | -0.076 | 0.233 | 0.301 |  |
|  | NCS | Delta |  | -0.079 | 0.417 | 0.501 |  | -0.072 | 0.262 | 0.325 |  |
|  |  | MC |  | -0.074 | 0.413 | 0.495 |  | -0.069 | 0.260 | 0.322 |  |
|  | WnoF | Delta | 0.010 | -0.140 | 0.247 | 0.295 |  | -0.099 | 0.152 | 0.189 |  |
|  |  | MC |  | -0.144 | 0.249 | 0.297 |  | -0.101 | 0.153 | 0.190 |  |
|  | Weibull | Delta |  | -0.106 | 0.222 | 0.266 |  | -0.070 | 0.129 | 0.163 |  |
|  |  | MC |  | -0.119 | 0.228 | 0.272 |  | -0.081 | 0.132 | 0.168 |  |
|  | PMS | Delta |  | 0.006 | 0.293 | 0.364 |  | 0.005 | 0.173 | 0.217 |  |
|  |  | MC |  | 0.020 | 0.293 | 0.361 |  | 0.013 | 0.173 | 0.216 |  |
|  | PTPRS | Delta |  | -0.051 | 0.256 | 0.326 |  | -0.026 | 0.160 | 0.201 |  |
|  |  | MC |  | -0.055 | 0.257 | 0.327 |  | -0.030 | 0.161 | 0.203 |  |
|  | NCS | Delta |  | -0.024 | 0.305 | 0.372 |  | -0.007 | 0.184 | 0.226 |  |
|  |  | MC |  | -0.022 | 0.303 | 0.369 |  | -0.006 | 0.183 | 0.225 |  |
| 30% | WnoF | Delta | 0.002 | -0.427 | 0.552 | 0.654 | <1min | -0.373 | 0.409 | 0.488 | <1min |
|  |  | MC |  | -0.441 | 0.561 | 0.663 | 3min | -0.383 | 0.416 | 0.496 | 3min |
|  | Weibull | Delta |  | -0.291 | 0.459 | 0.551 | 2min | -0.268 | 0.321 | 0.395 | 4min |
|  |  | MC |  | -0.312 | 0.469 | 0.561 | 13min | -0.283 | 0.331 | 0.404 | 16min |
|  | PMS | Delta |  | -0.162 | 0.620 | 0.778 | 13min | -0.160 | 0.428 | 0.563 | 1hrs33min |
|  |  | MC |  | -0.133 | 0.594 | 0.747 | 29min | -0.134 | 0.413 | 0.542 | 1hrs49min |
|  | PTPRS | Delta |  | -0.220 | 0.544 | 0.705 | 2hrs40min | -0.207 | 0.396 | 0.512 | 2hrs52min |
|  |  | MC |  | -0.229 | 0.547 | 0.707 | 67hrs3min | -0.213 | 0.399 | 0.515 | 68hrs55min |
|  | NCS | Delta |  | -0.128 | 0.552 | 0.682 | 1hrs30min | -0.189 | 0.416 | 0.538 | 1hrs31min |
|  |  | MC |  | -0.120 | 0.545 | 0.672 | 23hrs15min | -0.183 | 0.411 | 0.532 | 23hrs58min |
|  | WnoF | Delta | 0.005 | -0.250 | 0.369 | 0.442 |  | -0.216 | 0.259 | 0.312 |  |
|  |  | MC |  | -0.260 | 0.375 | 0.447 |  | -0.224 | 0.264 | 0.317 |  |
|  | Weibull | Delta |  | -0.152 | 0.314 | 0.377 |  | -0.140 | 0.202 | 0.250 |  |
|  |  | MC |  | -0.167 | 0.319 | 0.381 |  | -0.151 | 0.206 | 0.255 |  |
|  | PMS | Delta |  | -0.018 | 0.405 | 0.506 |  | -0.031 | 0.258 | 0.339 |  |
|  |  | MC |  | 0.002 | 0.401 | 0.497 |  | -0.017 | 0.257 | 0.335 |  |
|  | PTPRS | Delta |  | -0.084 | 0.365 | 0.460 |  | -0.080 | 0.238 | 0.306 |  |
|  |  | MC |  | -0.091 | 0.366 | 0.462 |  | -0.083 | 0.239 | 0.306 |  |
|  | NCS | Delta |  | -0.019 | 0.394 | 0.485 |  | -0.054 | 0.274 | 0.348 |  |
|  |  | MC |  | -0.017 | 0.390 | 0.479 |  | -0.053 | 0.271 | 0.345 |  |
|  | WnoF | Delta | 0.010 | -0.153 | 0.266 | 0.322 |  | -0.129 | 0.175 | 0.214 |  |
|  |  | MC |  | -0.159 | 0.269 | 0.324 |  | -0.134 | 0.177 | 0.216 |  |
|  | Weibull | Delta |  | -0.083 | 0.233 | 0.281 |  | -0.075 | 0.141 | 0.175 |  |
|  |  | MC |  | -0.096 | 0.237 | 0.284 |  | -0.085 | 0.145 | 0.179 |  |
|  | PMS | Delta |  | 0.017 | 0.291 | 0.363 |  | -0.006 | 0.186 | 0.236 |  |
|  |  | MC |  | 0.031 | 0.291 | 0.361 |  | 0.004 | 0.187 | 0.235 |  |
|  | PTPRS | Delta |  | -0.034 | 0.267 | 0.335 |  | -0.035 | 0.165 | 0.209 |  |
|  |  | MC |  | -0.038 | 0.269 | 0.336 |  | -0.037 | 0.166 | 0.209 |  |
|  | NCS | Delta |  | 0.011 | 0.293 | 0.364 |  | -0.006 | 0.193 | 0.240 |  |
|  |  | MC |  | 0.009 | 0.289 | 0.360 |  | -0.008 | 0.191 | 0.239 |  |
| 60% | WnoF | Delta | 0.002 | -0.342 | 0.544 | 0.695 | <1min | -0.327 | 0.396 | 0.493 | <1min |
|  |  | MC |  | -0.353 | 0.546 | 0.697 | 3min | -0.335 | 0.401 | 0.497 | 3min |
|  | Weibull | Delta |  | -0.253 | 0.510 | 0.646 | 2min | -0.232 | 0.339 | 0.430 | 4min |
|  |  | MC |  | -0.281 | 0.518 | 0.658 | 14min | -0.255 | 0.350 | 0.443 | 16min |
|  | PMS | Delta |  | -0.157 | 0.598 | 0.755 | 6min | -0.130 | 0.424 | 0.557 | 13min |
|  |  | MC |  | -0.128 | 0.578 | 0.732 | 22min | -0.104 | 0.413 | 0.543 | 30min |
|  | PTPRS | Delta |  | -0.208 | 0.532 | 0.693 | 2hrs4min | -0.184 | 0.397 | 0.533 | 2hrs59min |
|  |  | MC |  | -0.214 | 0.531 | 0.694 | 69hrs56min | -0.186 | 0.395 | 0.522 | 71hrs54min |
|  | NCS | Delta |  | -0.138 | 0.560 | 0.685 | 1hrs30min | -0.140 | 0.423 | 0.541 | 1hrs32min |
|  |  | MC |  | -0.142 | 0.546 | 0.671 | 23hrs28min | -0.141 | 0.417 | 0.535 | 24hrs35min |
|  | WnoF | Delta | 0.005 | -0.184 | 0.376 | 0.473 |  | -0.176 | 0.248 | 0.313 |  |
|  |  | MC |  | -0.191 | 0.376 | 0.474 |  | -0.182 | 0.250 | 0.315 |  |
|  | Weibull | Delta |  | -0.126 | 0.358 | 0.447 |  | -0.115 | 0.220 | 0.279 |  |
|  |  | MC |  | -0.146 | 0.363 | 0.453 |  | -0.129 | 0.226 | 0.286 |  |
|  | PMS | Delta |  | -0.018 | 0.397 | 0.496 |  | -0.015 | 0.256 | 0.336 |  |
|  |  | MC |  | 0.002 | 0.389 | 0.486 |  | 0.001 | 0.253 | 0.331 |  |
|  | PTPRS | Delta |  | -0.080 | 0.356 | 0.455 |  | -0.058 | 0.235 | 0.305 |  |
|  |  | MC |  | -0.083 | 0.355 | 0.455 |  | -0.062 | 0.236 | 0.307 |  |
|  | NCS | Delta |  | -0.025 | 0.383 | 0.482 |  | -0.034 | 0.265 | 0.343 |  |
|  |  | MC |  | -0.033 | 0.375 | 0.475 |  | -0.039 | 0.262 | 0.340 |  |
|  | WnoF | Delta | 0.010 | -0.102 | 0.278 | 0.347 |  | -0.097 | 0.169 | 0.215 |  |
|  |  | MC |  | -0.109 | 0.279 | 0.347 |  | -0.102 | 0.170 | 0.217 |  |
|  | Weibull | Delta |  | -0.066 | 0.269 | 0.333 |  | -0.058 | 0.154 | 0.198 |  |
|  |  | MC |  | -0.081 | 0.270 | 0.336 |  | -0.068 | 0.157 | 0.201 |  |
|  | PMS | Delta |  | 0.009 | 0.287 | 0.358 |  | 0.000 | 0.179 | 0.230 |  |
|  |  | MC |  | 0.023 | 0.286 | 0.355 |  | 0.010 | 0.178 | 0.227 |  |
|  | PTPRS | Delta |  | -0.035 | 0.267 | 0.336 |  | -0.024 | 0.169 | 0.211 |  |
|  |  | MC |  | -0.036 | 0.265 | 0.335 |  | -0.026 | 0.169 | 0.211 |  |
|  | NCS | Delta |  | 0.006 | 0.292 | 0.367 |  | 0.001 | 0.181 | 0.233 |  |
|  |  | MC |  | -0.004 | 0.287 | 0.363 |  | -0.007 | 0.180 | 0.232 |  |
| 90% | WnoF | Delta | 0.002 | -0.221 | 0.554 | 0.714 | <1min | -0.231 | 0.396 | 0.524 | <1min |
|  |  | MC |  | -0.218 | 0.546 | 0.705 | 4min | -0.228 | 0.391 | 0.519 | 3min |
|  | Weibull | Delta |  | -0.214 | 0.549 | 0.708 | 2min | -0.225 | 0.392 | 0.519 | 3min |
|  |  | MC |  | -0.239 | 0.563 | 0.723 | 17min | -0.231 | 0.394 | 0.523 | 15min |
|  | PMS | Delta |  | -0.151 | 0.603 | 0.765 | 3min | -0.112 | 0.439 | 0.570 | 7min |
|  |  | MC |  | -0.129 | 0.579 | 0.736 | 23min | -0.096 | 0.426 | 0.551 | 24min |
|  | PTPRS | Delta |  | -0.177 | 0.546 | 0.724 | 3hrs37min | -0.173 | 0.413 | 0.548 | 4hrs38min |
|  |  | MC |  | -0.176 | 0.541 | 0.716 | 74hrs11min | -0.176 | 0.414 | 0.547 | 66hrs1min |
|  | NCS | Delta |  | -0.209 | 0.620 | 0.823 | 1hrs32min | -0.103 | 0.453 | 0.620 | 1hrs34min |
|  |  | MC |  | -0.273 | 0.569 | 0.774 | 29hrs38min | -0.130 | 0.423 | 0.578 | 25hrs8min |
|  | WnoF | Delta | 0.005 | -0.094 | 0.387 | 0.493 |  | -0.104 | 0.257 | 0.338 |  |
|  |  | MC |  | -0.097 | 0.381 | 0.486 |  | -0.106 | 0.255 | 0.335 |  |
|  | Weibull | Delta |  | -0.092 | 0.385 | 0.490 |  | -0.102 | 0.255 | 0.336 |  |
|  |  | MC |  | -0.106 | 0.389 | 0.496 |  | -0.108 | 0.254 | 0.336 |  |
|  | PMS | Delta |  | 0.000 | 0.395 | 0.502 |  | -0.016 | 0.270 | 0.348 |  |
|  |  | MC |  | 0.008 | 0.390 | 0.492 |  | -0.006 | 0.270 | 0.346 |  |
|  | PTPRS | Delta |  | -0.049 | 0.366 | 0.475 |  | -0.053 | 0.252 | 0.327 |  |
|  |  | MC |  | -0.048 | 0.362 | 0.472 |  | -0.055 | 0.251 | 0.327 |  |
|  | NCS | Delta |  | -0.023 | 0.402 | 0.517 |  | -0.025 | 0.275 | 0.355 |  |
|  |  | MC |  | -0.078 | 0.375 | 0.484 |  | -0.051 | 0.271 | 0.354 |  |
|  | WnoF | Delta | 0.010 | -0.035 | 0.293 | 0.368 |  | -0.042 | 0.185 | 0.240 |  |
|  |  | MC |  | -0.041 | 0.289 | 0.364 |  | -0.047 | 0.183 | 0.239 |  |
|  | Weibull | Delta |  | -0.035 | 0.292 | 0.367 |  | -0.042 | 0.184 | 0.240 |  |
|  |  | MC |  | -0.044 | 0.294 | 0.369 |  | -0.049 | 0.185 | 0.241 |  |
|  | PMS | Delta |  | 0.023 | 0.295 | 0.366 |  | -0.006 | 0.195 | 0.246 |  |
|  |  | MC |  | 0.032 | 0.294 | 0.366 |  | 0.004 | 0.195 | 0.246 |  |
|  | PTPRS | Delta |  | -0.010 | 0.283 | 0.357 |  | -0.024 | 0.191 | 0.244 |  |
|  |  | MC |  | -0.009 | 0.279 | 0.353 |  | -0.024 | 0.191 | 0.244 |  |
|  | NCS | Delta |  | 0.019 | 0.294 | 0.363 |  | -0.029 | 0.209 | 0.260 |  |
|  |  | MC |  | -0.006 | 0.290 | 0.357 |  | -0.043 | 0.210 | 0.263 |  |

Note: Metrics are calculated based on the logarithms of TTB estimates. The number of MC samples is 2,000. For MC method, the estimates are the median of the MC samples. Time is for all ARR values for each Model. ARR: absolute relative risk; MAE: mean absolute error; RMSE: root mean squared error; MC: Monte Carlo; NCS: natural cubic spline; PMS: penalized M-spline; PTPRS: penalized thin plate regression spline; SPRINT: Systolic Blood Pressure Intervention Trial; TTB: time to benefit; WF: Weibull model with shared frailty; WnoF: Weibull model without shared frailty

**Table S11.** TTB interval estimation results for simulation scenario II with the Gamma frailty and 100 replicates.

|  |  |  |  | N=5,000 | | | | N=10,000 | | | |
| --- | --- | --- | --- | --- | --- | --- | --- | --- | --- | --- | --- |
| Cesnoring | Model | Method | ARR | CP | OLLP | OULP | length | CP | OLLP | OULP | length |
| 0% | WnoF | Delta | 0.002 | 0.830 | 0.020 | 0.150 | 1.789 | 0.850 | 0.000 | 0.150 | 1.290 |
|  |  | MC |  | 0.890 | 0.000 | 0.110 | 1.731 | 0.860 | 0.000 | 0.140 | 1.270 |
|  | WF | Delta |  | 0.840 | 0.010 | 0.150 | 1.628 | 0.848 | 0.000 | 0.152 | 1.180 |
|  |  | MC |  | 0.860 | 0.000 | 0.140 | 1.577 | 0.848 | 0.000 | 0.152 | 1.159 |
|  | PMS | Delta |  | 0.810 | 0.090 | 0.100 | 2.992 | 0.895 | 0.053 | 0.053 | 2.090 |
|  |  | MC |  | 0.900 | 0.040 | 0.060 | 2.525 | 0.937 | 0.021 | 0.042 | 1.916 |
|  | PTPRS | Delta |  | 0.900 | 0.030 | 0.070 | 2.490 | 0.940 | 0.010 | 0.050 | 1.960 |
|  |  | MC |  | 0.890 | 0.020 | 0.090 | 2.420 | 0.960 | 0.000 | 0.040 | 1.949 |
|  | NCS | Delta |  | 0.800 | 0.070 | 0.130 | 2.290 | 0.840 | 0.030 | 0.130 | 1.864 |
|  |  | MC |  | 0.890 | 0.030 | 0.080 | 1.993 | 0.930 | 0.000 | 0.070 | 1.658 |
|  | WnoF | Delta | 0.005 | 0.860 | 0.020 | 0.120 | 1.312 | 0.880 | 0.000 | 0.120 | 0.942 |
|  |  | MC |  | 0.900 | 0.010 | 0.090 | 1.299 | 0.900 | 0.000 | 0.100 | 0.944 |
|  | WF | Delta |  | 0.880 | 0.010 | 0.110 | 1.195 | 0.889 | 0.010 | 0.101 | 0.862 |
|  |  | MC |  | 0.890 | 0.010 | 0.100 | 1.175 | 0.899 | 0.000 | 0.101 | 0.851 |
|  | PMS | Delta |  | 0.840 | 0.090 | 0.070 | 1.836 | 0.916 | 0.053 | 0.032 | 1.291 |
|  |  | MC |  | 0.900 | 0.040 | 0.060 | 1.756 | 0.947 | 0.032 | 0.021 | 1.269 |
|  | PTPRS | Delta |  | 0.890 | 0.030 | 0.080 | 1.636 | 0.960 | 0.010 | 0.030 | 1.237 |
|  |  | MC |  | 0.900 | 0.030 | 0.070 | 1.624 | 0.970 | 0.000 | 0.030 | 1.239 |
|  | NCS | Delta |  | 0.840 | 0.070 | 0.090 | 1.658 | 0.920 | 0.040 | 0.040 | 1.305 |
|  |  | MC |  | 0.880 | 0.050 | 0.070 | 1.488 | 0.950 | 0.020 | 0.030 | 1.192 |
|  | WnoF | Delta | 0.010 | 0.900 | 0.020 | 0.080 | 1.020 | 0.930 | 0.000 | 0.070 | 0.729 |
|  |  | MC |  | 0.930 | 0.020 | 0.050 | 1.015 | 0.960 | 0.000 | 0.040 | 0.731 |
|  | WF | Delta |  | 0.940 | 0.010 | 0.050 | 0.932 | 0.929 | 0.010 | 0.061 | 0.670 |
|  |  | MC |  | 0.940 | 0.010 | 0.050 | 0.927 | 0.929 | 0.010 | 0.061 | 0.666 |
|  | PMS | Delta |  | 0.860 | 0.080 | 0.060 | 1.227 | 0.968 | 0.032 | 0.000 | 0.892 |
|  |  | MC |  | 0.890 | 0.050 | 0.060 | 1.240 | 0.979 | 0.021 | 0.000 | 0.891 |
|  | PTPRS | Delta |  | 0.910 | 0.030 | 0.060 | 1.172 | 0.970 | 0.010 | 0.020 | 0.873 |
|  |  | MC |  | 0.900 | 0.030 | 0.070 | 1.161 | 0.980 | 0.000 | 0.020 | 0.867 |
|  | NCS | Delta |  | 0.860 | 0.070 | 0.070 | 1.242 | 0.940 | 0.040 | 0.020 | 0.952 |
|  |  | MC |  | 0.890 | 0.050 | 0.060 | 1.140 | 0.950 | 0.030 | 0.020 | 0.885 |
| 30% | WnoF | Delta | 0.002 | 0.820 | 0.020 | 0.160 | 1.927 | 0.870 | 0.000 | 0.130 | 1.399 |
|  |  | MC |  | 0.850 | 0.000 | 0.150 | 1.804 | 0.870 | 0.000 | 0.130 | 1.330 |
|  | WF | Delta |  | 0.866 | 0.021 | 0.113 | 1.791 | 0.900 | 0.000 | 0.100 | 1.303 |
|  |  | MC |  | 0.876 | 0.010 | 0.113 | 1.705 | 0.880 | 0.000 | 0.120 | 1.259 |
|  | PMS | Delta |  | 0.820 | 0.100 | 0.080 | 2.943 | 0.900 | 0.040 | 0.060 | 2.221 |
|  |  | MC |  | 0.910 | 0.040 | 0.050 | 2.611 | 0.940 | 0.010 | 0.050 | 2.022 |
|  | PTPRS | Delta |  | 0.880 | 0.040 | 0.080 | 2.535 | 0.940 | 0.010 | 0.050 | 2.016 |
|  |  | MC |  | 0.920 | 0.020 | 0.060 | 2.460 | 0.950 | 0.000 | 0.050 | 1.999 |
|  | NCS | Delta |  | 0.780 | 0.100 | 0.120 | 2.435 | 0.870 | 0.030 | 0.100 | 1.941 |
|  |  | MC |  | 0.910 | 0.030 | 0.060 | 2.076 | 0.930 | 0.010 | 0.060 | 1.718 |
|  | WnoF | Delta | 0.005 | 0.850 | 0.020 | 0.130 | 1.406 | 0.870 | 0.000 | 0.130 | 1.012 |
|  |  | MC |  | 0.900 | 0.010 | 0.090 | 1.355 | 0.880 | 0.000 | 0.120 | 0.985 |
|  | WF | Delta |  | 0.897 | 0.031 | 0.072 | 1.312 | 0.910 | 0.010 | 0.080 | 0.946 |
|  |  | MC |  | 0.918 | 0.010 | 0.072 | 1.269 | 0.910 | 0.000 | 0.090 | 0.922 |
|  | PMS | Delta |  | 0.850 | 0.100 | 0.050 | 1.868 | 0.920 | 0.040 | 0.040 | 1.345 |
|  |  | MC |  | 0.900 | 0.040 | 0.060 | 1.814 | 0.950 | 0.010 | 0.040 | 1.329 |
|  | PTPRS | Delta |  | 0.900 | 0.040 | 0.060 | 1.716 | 0.960 | 0.010 | 0.030 | 1.265 |
|  |  | MC |  | 0.920 | 0.020 | 0.060 | 1.652 | 0.960 | 0.010 | 0.030 | 1.265 |
|  | NCS | Delta |  | 0.820 | 0.110 | 0.070 | 1.724 | 0.900 | 0.050 | 0.050 | 1.335 |
|  |  | MC |  | 0.890 | 0.050 | 0.060 | 1.543 | 0.950 | 0.010 | 0.040 | 1.223 |
|  | WnoF | Delta | 0.010 | 0.920 | 0.020 | 0.060 | 1.089 | 0.930 | 0.000 | 0.070 | 0.779 |
|  |  | MC |  | 0.930 | 0.020 | 0.050 | 1.071 | 0.940 | 0.000 | 0.060 | 0.773 |
|  | WF | Delta |  | 0.918 | 0.031 | 0.052 | 1.019 | 0.940 | 0.010 | 0.050 | 0.731 |
|  |  | MC |  | 0.938 | 0.010 | 0.052 | 0.996 | 0.940 | 0.010 | 0.050 | 0.718 |
|  | PMS | Delta |  | 0.860 | 0.080 | 0.060 | 1.283 | 0.930 | 0.040 | 0.030 | 0.919 |
|  |  | MC |  | 0.900 | 0.040 | 0.060 | 1.280 | 0.960 | 0.020 | 0.020 | 0.926 |
|  | PTPRS | Delta |  | 0.900 | 0.030 | 0.070 | 1.208 | 0.960 | 0.010 | 0.030 | 0.895 |
|  |  | MC |  | 0.910 | 0.030 | 0.060 | 1.192 | 0.960 | 0.010 | 0.030 | 0.890 |
|  | NCS | Delta |  | 0.830 | 0.100 | 0.070 | 1.264 | 0.940 | 0.040 | 0.020 | 0.957 |
|  |  | MC |  | 0.880 | 0.050 | 0.070 | 1.176 | 0.970 | 0.010 | 0.020 | 0.900 |
| 60% | WnoF | Delta | 0.002 | 0.830 | 0.020 | 0.150 | 2.109 | 0.910 | 0.000 | 0.090 | 1.574 |
|  |  | MC |  | 0.860 | 0.010 | 0.130 | 1.936 | 0.900 | 0.000 | 0.100 | 1.471 |
|  | WF | Delta |  | 0.838 | 0.030 | 0.131 | 2.015 | 0.920 | 0.010 | 0.070 | 1.503 |
|  |  | MC |  | 0.869 | 0.010 | 0.121 | 1.926 | 0.930 | 0.000 | 0.070 | 1.450 |
|  | PMS | Delta |  | 0.850 | 0.080 | 0.070 | 3.014 | 0.870 | 0.070 | 0.060 | 2.216 |
|  |  | MC |  | 0.910 | 0.040 | 0.050 | 2.594 | 0.950 | 0.010 | 0.040 | 2.053 |
|  | PTPRS | Delta |  | 0.880 | 0.040 | 0.080 | 2.437 | 0.930 | 0.010 | 0.060 | 2.007 |
|  |  | MC |  | 0.910 | 0.020 | 0.070 | 2.400 | 0.950 | 0.000 | 0.050 | 2.065 |
|  | NCS | Delta |  | 0.810 | 0.090 | 0.100 | 2.812 | 0.840 | 0.080 | 0.080 | 1.994 |
|  |  | MC |  | 0.880 | 0.040 | 0.080 | 2.244 | 0.930 | 0.020 | 0.050 | 1.772 |
|  | WnoF | Delta | 0.005 | 0.850 | 0.030 | 0.120 | 1.526 | 0.930 | 0.000 | 0.070 | 1.118 |
|  |  | MC |  | 0.860 | 0.020 | 0.120 | 1.429 | 0.940 | 0.000 | 0.060 | 1.063 |
|  | WF | Delta |  | 0.869 | 0.030 | 0.101 | 1.466 | 0.940 | 0.010 | 0.050 | 1.075 |
|  |  | MC |  | 0.889 | 0.020 | 0.091 | 1.426 | 0.950 | 0.000 | 0.050 | 1.058 |
|  | PMS | Delta |  | 0.870 | 0.080 | 0.050 | 1.818 | 0.910 | 0.050 | 0.040 | 1.328 |
|  |  | MC |  | 0.910 | 0.040 | 0.050 | 1.807 | 0.930 | 0.030 | 0.040 | 1.323 |
|  | PTPRS | Delta |  | 0.900 | 0.030 | 0.070 | 1.646 | 0.960 | 0.010 | 0.030 | 1.248 |
|  |  | MC |  | 0.920 | 0.020 | 0.060 | 1.623 | 0.960 | 0.010 | 0.030 | 1.288 |
|  | NCS | Delta |  | 0.830 | 0.080 | 0.090 | 1.890 | 0.910 | 0.050 | 0.040 | 1.338 |
|  |  | MC |  | 0.890 | 0.040 | 0.070 | 1.656 | 0.950 | 0.010 | 0.040 | 1.253 |
|  | WnoF | Delta | 0.010 | 0.880 | 0.030 | 0.090 | 1.163 | 0.950 | 0.010 | 0.040 | 0.842 |
|  |  | MC |  | 0.890 | 0.030 | 0.080 | 1.104 | 0.940 | 0.010 | 0.050 | 0.807 |
|  | WF | Delta |  | 0.889 | 0.030 | 0.081 | 1.121 | 0.950 | 0.010 | 0.040 | 0.814 |
|  |  | MC |  | 0.889 | 0.030 | 0.081 | 1.106 | 0.950 | 0.010 | 0.040 | 0.812 |
|  | PMS | Delta |  | 0.870 | 0.070 | 0.060 | 1.264 | 0.950 | 0.030 | 0.020 | 0.897 |
|  |  | MC |  | 0.900 | 0.030 | 0.070 | 1.287 | 0.960 | 0.020 | 0.020 | 0.921 |
|  | PTPRS | Delta |  | 0.890 | 0.030 | 0.080 | 1.187 | 0.960 | 0.010 | 0.030 | 0.888 |
|  |  | MC |  | 0.910 | 0.020 | 0.070 | 1.181 | 0.970 | 0.010 | 0.020 | 0.900 |
|  | NCS | Delta |  | 0.860 | 0.080 | 0.060 | 1.260 | 0.940 | 0.040 | 0.020 | 0.942 |
|  |  | MC |  | 0.880 | 0.040 | 0.080 | 1.253 | 0.970 | 0.010 | 0.020 | 0.916 |
| 90% | WnoF | Delta | 0.002 | 0.920 | 0.020 | 0.060 | 2.566 | 0.920 | 0.010 | 0.070 | 1.924 |
|  |  | MC |  | 0.930 | 0.020 | 0.050 | 2.436 | 0.930 | 0.000 | 0.070 | 1.829 |
|  | WF | Delta |  | 0.920 | 0.020 | 0.060 | 2.543 | 0.910 | 0.010 | 0.080 | 1.908 |
|  |  | MC |  | 0.930 | 0.020 | 0.050 | 2.474 | 0.920 | 0.000 | 0.080 | 1.849 |
|  | PMS | Delta |  | 0.810 | 0.100 | 0.090 | 2.999 | 0.860 | 0.090 | 0.050 | 2.143 |
|  |  | MC |  | 0.920 | 0.030 | 0.050 | 2.790 | 0.940 | 0.020 | 0.040 | 2.169 |
|  | PTPRS | Delta |  | 0.890 | 0.050 | 0.060 | 2.686 | 0.880 | 0.040 | 0.080 | 2.027 |
|  |  | MC |  | 0.930 | 0.010 | 0.060 | 9.206 | 0.930 | 0.010 | 0.060 | 2.141 |
|  | NCS | Delta |  | 0.750 | 0.150 | 0.100 | 2.027 | 0.720 | 0.190 | 0.090 | 1.763 |
|  |  | MC |  | 0.930 | 0.030 | 0.040 | 7.773 | 0.940 | 0.020 | 0.040 | 2.586 |
|  | WnoF | Delta | 0.005 | 0.930 | 0.020 | 0.050 | 1.806 | 0.950 | 0.010 | 0.040 | 1.322 |
|  |  | MC |  | 0.940 | 0.010 | 0.050 | 1.784 | 0.960 | 0.010 | 0.030 | 1.290 |
|  | WF | Delta |  | 0.930 | 0.020 | 0.050 | 1.792 | 0.950 | 0.010 | 0.040 | 1.313 |
|  |  | MC |  | 0.940 | 0.010 | 0.050 | 1.820 | 0.940 | 0.010 | 0.050 | 1.319 |
|  | PMS | Delta |  | 0.880 | 0.080 | 0.040 | 1.986 | 0.910 | 0.060 | 0.030 | 1.307 |
|  |  | MC |  | 0.920 | 0.030 | 0.050 | 2.044 | 0.960 | 0.010 | 0.030 | 1.431 |
|  | PTPRS | Delta |  | 0.910 | 0.040 | 0.050 | 1.811 | 0.950 | 0.010 | 0.040 | 1.286 |
|  |  | MC |  | 0.930 | 0.010 | 0.060 | 8.426 | 0.950 | 0.010 | 0.040 | 1.470 |
|  | NCS | Delta |  | 0.760 | 0.190 | 0.050 | 1.731 | 0.820 | 0.120 | 0.060 | 1.145 |
|  |  | MC |  | 0.930 | 0.020 | 0.050 | 7.230 | 0.940 | 0.010 | 0.050 | 1.621 |
|  | WnoF | Delta | 0.010 | 0.950 | 0.010 | 0.040 | 1.353 | 0.960 | 0.010 | 0.030 | 0.971 |
|  |  | MC |  | 0.950 | 0.010 | 0.040 | 1.387 | 0.960 | 0.010 | 0.030 | 0.972 |
|  | WF | Delta |  | 0.950 | 0.010 | 0.040 | 1.345 | 0.960 | 0.010 | 0.030 | 0.968 |
|  |  | MC |  | 0.950 | 0.010 | 0.040 | 1.438 | 0.960 | 0.010 | 0.030 | 0.997 |
|  | PMS | Delta |  | 0.870 | 0.080 | 0.050 | 1.352 | 0.930 | 0.040 | 0.030 | 0.989 |
|  |  | MC |  | 0.940 | 0.020 | 0.040 | 1.602 | 0.960 | 0.020 | 0.020 | 1.050 |
|  | PTPRS | Delta |  | 0.930 | 0.020 | 0.050 | 1.314 | 0.950 | 0.010 | 0.040 | 0.951 |
|  |  | MC |  | 0.940 | 0.020 | 0.040 | 7.992 | 0.960 | 0.010 | 0.030 | 1.131 |
|  | NCS | Delta |  | 0.790 | 0.140 | 0.070 | 1.111 | 0.900 | 0.040 | 0.060 | 0.975 |
|  |  | MC |  | 0.950 | 0.000 | 0.050 | 6.752 | 0.950 | 0.020 | 0.030 | 1.238 |

Note: Metrics are calculated based on the logarithms of TTB estimates. The number of MC samples is 2,000. ARR: absolute relative risk; CP: coverage probability; OLLP: out-of-lower-limit probability; OULP: out-of-upper-limit probability; MAE: mean absolute error; RMSE: root mean squared error; MC: Monte Carlo; NCS: natural cubic spline; PMS: penalized M-spline; PTPRS: penalized thin plate regression spline; SPRINT: Systolic Blood Pressure Intervention Trial; TTB: time to benefit; WF: Weibull model with shared frailty; WnoF: Weibull model without shared frailty

**Table S12.** TTB point estimation results for simulation scenario II with the log-normal frailty and 1,000 replicates.

|  |  |  |  | N=5,000 | | | | N=10,000 | | | |
| --- | --- | --- | --- | --- | --- | --- | --- | --- | --- | --- | --- |
| Cesnoring | Model | Method | ARR | Bias | MAE | RMSE | Time | Bias | MAE | RMSE | Time |
| 0% | WnoF | Delta | 0.002 | -0.275 | 0.427 | 0.533 | 1min | -0.267 | 0.346 | 0.423 | 1min |
|  |  | MC |  | -0.265 | 0.424 | 0.529 | 1hrs31min | -0.260 | 0.343 | 0.419 | 1hrs30min |
|  | WF | Delta |  | -0.329 | 0.518 | 0.697 | 3hrs12min | -0.236 | 0.319 | 0.391 | 5hrs8min |
|  |  | MC |  | -0.331 | 0.522 | 0.708 | 6hrs2min | -0.233 | 0.317 | 0.389 | 8hrs2min |
|  | PMS | Delta |  | -0.497 | 0.752 | 0.894 | 17hrs3min | -0.390 | 0.597 | 0.747 | 30hrs46min |
|  |  | MC |  | -0.500 | 0.748 | 0.890 | 20hrs20min | -0.388 | 0.592 | 0.746 | 33hrs10min |
|  | PTPRS | Delta |  | -0.207 | 0.514 | 0.666 | 30hrs45min | -0.172 | 0.391 | 0.506 | 43hrs21min |
|  | NCS | Delta |  | -0.615 | 0.650 | 0.729 | 3hrs38min | -0.621 | 0.632 | 0.686 | 3hrs30min |
|  | WnoF | Delta | 0.005 | -0.152 | 0.296 | 0.368 |  | -0.147 | 0.229 | 0.281 |  |
|  |  | MC |  | -0.144 | 0.294 | 0.367 |  | -0.141 | 0.227 | 0.278 |  |
|  | WF | Delta |  | -0.240 | 0.459 | 0.687 |  | -0.123 | 0.212 | 0.263 |  |
|  |  | MC |  | -0.239 | 0.455 | 0.678 |  | -0.121 | 0.212 | 0.263 |  |
|  | PMS | Delta |  | -0.175 | 0.452 | 0.559 |  | 0.039 | 0.358 | 0.460 |  |
|  |  | MC |  | -0.179 | 0.449 | 0.556 |  | 0.046 | 0.352 | 0.452 |  |
|  | PTPRS | Delta |  | -0.080 | 0.334 | 0.427 |  | -0.057 | 0.246 | 0.310 |  |
|  | NCS | Delta |  | -0.319 | 0.379 | 0.445 |  | -0.318 | 0.343 | 0.392 |  |
|  | WnoF | Delta | 0.010 | -0.084 | 0.219 | 0.274 |  | -0.080 | 0.163 | 0.201 |  |
|  |  | MC |  | -0.078 | 0.219 | 0.273 |  | -0.075 | 0.162 | 0.200 |  |
|  | WF | Delta |  | -0.151 | 0.418 | 0.655 |  | -0.067 | 0.158 | 0.199 |  |
|  |  | MC |  | -0.156 | 0.417 | 0.655 |  | -0.066 | 0.158 | 0.199 |  |
|  | PMS | Delta |  | -0.020 | 0.316 | 0.397 |  | 0.302 | 0.396 | 0.487 |  |
|  |  | MC |  | -0.026 | 0.314 | 0.395 |  | 0.300 | 0.392 | 0.480 |  |
|  | PTPRS | Delta |  | -0.031 | 0.237 | 0.302 |  | -0.018 | 0.173 | 0.217 |  |
|  | NCS | Delta |  | -0.170 | 0.255 | 0.308 |  | -0.166 | 0.211 | 0.250 |  |
| 30% | WnoF | Delta | 0.002 | -0.335 | 0.473 | 0.592 | 1min | -0.322 | 0.393 | 0.479 | 1min |
|  |  | MC |  | -0.321 | 0.466 | 0.584 | 1hrs30min | -0.312 | 0.387 | 0.472 | 1hrs31min |
|  | WF | Delta |  | -0.222 | 0.412 | 0.520 | 3hrs36min | -0.314 | 0.550 | 0.731 | 6hrs24min |
|  |  | MC |  | -0.207 | 0.407 | 0.515 | 5hrs29min | -0.310 | 0.546 | 0.727 | 9hrs14min |
|  | PMS | Delta |  | -0.313 | 0.673 | 0.818 | 12hrs4min | -0.252 | 0.526 | 0.652 | 27hrs54min |
|  |  | MC |  | -0.319 | 0.668 | 0.813 | 15hrs26min | -0.256 | 0.524 | 0.650 | 30hrs21min |
|  | PTPRS | Delta |  | -0.194 | 0.511 | 0.671 | 30hrs49min | -0.155 | 0.395 | 0.516 | 43hrs8min |
|  | NCS | Delta |  | -0.518 | 0.596 | 0.689 | 3hrs38min | -0.512 | 0.552 | 0.625 | 3hrs28min |
|  | WnoF | Delta | 0.005 | -0.188 | 0.321 | 0.403 |  | -0.181 | 0.257 | 0.314 |  |
|  |  | MC |  | -0.178 | 0.317 | 0.398 |  | -0.174 | 0.253 | 0.309 |  |
|  | WF | Delta |  | -0.108 | 0.290 | 0.371 |  | -0.211 | 0.564 | 0.768 |  |
|  |  | MC |  | -0.098 | 0.289 | 0.369 |  | -0.208 | 0.559 | 0.762 |  |
|  | PMS | Delta |  | -0.082 | 0.416 | 0.513 |  | -0.014 | 0.331 | 0.413 |  |
|  |  | MC |  | -0.089 | 0.413 | 0.510 |  | -0.019 | 0.329 | 0.411 |  |
|  | PTPRS | Delta |  | -0.069 | 0.331 | 0.427 |  | -0.046 | 0.248 | 0.316 |  |
|  | NCS | Delta |  | -0.250 | 0.360 | 0.431 |  | -0.243 | 0.306 | 0.364 |  |
|  | WnoF | Delta | 0.010 | -0.108 | 0.235 | 0.296 |  | -0.103 | 0.181 | 0.222 |  |
|  |  | MC |  | -0.101 | 0.233 | 0.293 |  | -0.098 | 0.179 | 0.220 |  |
|  | WF | Delta |  | -0.053 | 0.222 | 0.290 |  | -0.098 | 0.589 | 0.803 |  |
|  |  | MC |  | -0.047 | 0.222 | 0.290 |  | -0.100 | 0.584 | 0.797 |  |
|  | PMS | Delta |  | 0.010 | 0.285 | 0.355 |  | 0.082 | 0.253 | 0.318 |  |
|  |  | MC |  | 0.002 | 0.282 | 0.352 |  | 0.076 | 0.251 | 0.315 |  |
|  | PTPRS | Delta |  | -0.023 | 0.236 | 0.301 |  | -0.011 | 0.177 | 0.223 |  |
|  | NCS | MC |  | -0.119 | 0.251 | 0.308 |  | -0.111 | 0.199 | 0.243 |  |
| 60% | WnoF | Delta | 0.002 | -0.296 | 0.505 | 0.628 | 1min | -0.278 | 0.395 | 0.486 | 1min |
|  |  | Delta |  | -0.286 | 0.500 | 0.623 | 30min | -0.271 | 0.391 | 0.481 | 1hrs31min |
|  | WF | Delta |  | -0.205 | 0.461 | 0.576 | 3hrs36min | -0.226 | 0.471 | 0.647 | 7hrs49min |
|  |  | MC |  | -0.182 | 0.456 | 0.571 | 5hrs29min | -0.215 | 0.469 | 0.649 | 10hrs43min |
|  | PMS | Delta |  | -0.170 | 0.599 | 0.760 | 8hrs18min | -0.120 | 0.460 | 0.581 | 17hrs47min |
|  |  | MC |  | -0.184 | 0.593 | 0.754 | 12hrs48min | -0.130 | 0.458 | 0.579 | 20hrs19min |
|  | PTPRS | Delta |  | -0.191 | 0.529 | 0.697 | 57hrs30min | -0.156 | 0.412 | 0.538 | 42hrs29min |
|  | NCS | MC |  | -0.317 | 0.552 | 0.658 | 3hrs40min | -0.316 | 0.462 | 0.555 | 3hrs26min |
|  | WnoF | Delta | 0.005 | -0.154 | 0.344 | 0.428 |  | -0.143 | 0.259 | 0.318 |  |
|  |  | Delta |  | -0.148 | 0.341 | 0.425 |  | -0.139 | 0.257 | 0.316 |  |
|  | WF | Delta |  | -0.095 | 0.322 | 0.401 |  | -0.105 | 0.436 | 0.659 |  |
|  |  | MC |  | -0.078 | 0.320 | 0.400 |  | -0.098 | 0.430 | 0.652 |  |
|  | PMS | Delta |  | -0.028 | 0.379 | 0.486 |  | 0.002 | 0.284 | 0.353 |  |
|  |  | MC |  | -0.044 | 0.372 | 0.480 |  | -0.008 | 0.281 | 0.350 |  |
|  | PTPRS | Delta |  | -0.065 | 0.340 | 0.438 |  | -0.041 | 0.256 | 0.322 |  |
|  | NCS | MC |  | -0.122 | 0.361 | 0.442 |  | -0.116 | 0.286 | 0.348 |  |
|  | WnoF | Delta | 0.010 | -0.081 | 0.252 | 0.315 |  | -0.073 | 0.184 | 0.227 |  |
|  |  | Delta |  | -0.075 | 0.252 | 0.315 |  | -0.069 | 0.184 | 0.226 |  |
|  | WF | Delta |  | -0.044 | 0.240 | 0.301 |  | 0.010 | 0.411 | 0.652 |  |
|  |  | MC |  | -0.031 | 0.239 | 0.300 |  | 0.011 | 0.404 | 0.644 |  |
|  | PMS | Delta |  | 0.013 | 0.259 | 0.335 |  | 0.032 | 0.193 | 0.240 |  |
|  |  | MC |  | -0.002 | 0.256 | 0.331 |  | 0.021 | 0.191 | 0.238 |  |
|  | PTPRS | Delta |  | -0.024 | 0.243 | 0.312 |  | -0.008 | 0.183 | 0.227 |  |
|  | NCS | MC |  | -0.035 | 0.263 | 0.330 |  | -0.028 | 0.204 | 0.249 |  |
| 90% | WnoF | Delta | 0.002 | -0.219 | 0.561 | 0.723 | 1min | -0.199 | 0.423 | 0.537 | 1min |
|  |  | Delta |  | -0.208 | 0.559 | 0.718 | 1hrs40min | -0.190 | 0.423 | 0.534 | 1hrs35min |
|  | WF | Delta |  | -0.213 | 0.556 | 0.718 | 2hrs28min | -0.193 | 0.420 | 0.532 | 5hrs2min |
|  |  | MC |  | -0.190 | 0.554 | 0.712 | 7hrs52min | -0.178 | 0.418 | 0.529 | 8hrs5min |
|  | PMS | Delta |  | -0.157 | 0.597 | 0.776 | 7hrs44min | -0.113 | 0.461 | 0.593 | 13hrs7min |
|  |  | MC |  | -0.197 | 0.612 | 0.792 | 14hrs19min | -0.139 | 0.473 | 0.608 | 18hrs51min |
|  | PTPRS | Delta |  | -0.194 | 0.586 | 0.779 | 135hrs22min | -0.157 | 0.438 | 0.572 | 196hrs54min |
|  | NCS | MC |  | -0.108 | 0.547 | 0.691 | 3hrs42min | -0.094 | 0.435 | 0.544 | 3hrs15min |
|  | WnoF | Delta | 0.005 | -0.090 | 0.382 | 0.491 |  | -0.082 | 0.282 | 0.353 |  |
|  |  | Delta |  | -0.082 | 0.381 | 0.488 |  | -0.074 | 0.282 | 0.352 |  |
|  | WF | Delta |  | -0.089 | 0.380 | 0.489 |  | -0.081 | 0.281 | 0.351 |  |
|  |  | MC |  | -0.069 | 0.380 | 0.487 |  | -0.067 | 0.281 | 0.350 |  |
|  | PMS | Delta |  | -0.025 | 0.380 | 0.502 |  | -0.006 | 0.281 | 0.356 |  |
|  |  | MC |  | -0.044 | 0.385 | 0.510 |  | -0.015 | 0.286 | 0.362 |  |
|  | PTPRS | Delta |  | -0.058 | 0.377 | 0.491 |  | -0.043 | 0.276 | 0.347 |  |
|  | NCS | MC |  | -0.018 | 0.369 | 0.478 |  | -0.013 | 0.285 | 0.357 |  |
|  | WnoF | Delta | 0.010 | -0.029 | 0.278 | 0.362 |  | -0.027 | 0.205 | 0.254 |  |
|  |  | Delta |  | -0.024 | 0.276 | 0.357 |  | -0.020 | 0.205 | 0.254 |  |
|  | WF | Delta |  | -0.030 | 0.278 | 0.362 |  | -0.028 | 0.205 | 0.254 |  |
|  |  | MC |  | -0.014 | 0.277 | 0.358 |  | -0.015 | 0.206 | 0.254 |  |
|  | PMS | Delta |  | 0.006 | 0.273 | 0.356 |  | 0.011 | 0.199 | 0.250 |  |
|  |  | MC |  | 0.008 | 0.281 | 0.379 |  | 0.011 | 0.201 | 0.254 |  |
|  | PTPRS | Delta |  | -0.013 | 0.274 | 0.356 |  | -0.008 | 0.201 | 0.250 |  |
|  | NCS | MC |  | 0.004 | 0.271 | 0.353 |  | 0.009 | 0.200 | 0.254 |  |

Note: Metrics are calculated based on the logarithms of TTB estimates. The number of MC samples is 2,000. For MC method, the estimates are the median of the MC samples. Time is for all ARR values for each Model. ARR: absolute relative risk; MAE: mean absolute error; RMSE: root mean squared error; MC: Monte Carlo; NCS: natural cubic spline; PMS: penalized M-spline; PTPRS: penalized thin plate regression spline; SPRINT: Systolic Blood Pressure Intervention Trial; TTB: time to benefit; WF: Weibull model with shared frailty; WnoF: Weibull model without shared frailty

**Table S13.** TTB interval estimation results for simulation scenario II with the log-normal frailty and 1,000 replicates.

|  |  |  |  | N=5,000 | | | | N=10,000 | | | |
| --- | --- | --- | --- | --- | --- | --- | --- | --- | --- | --- | --- |
| Cesnoring | Model | Method | ARR | CP | OLLP | OULP | length | CP | OLLP | OULP | length |
| 0% | WnoF | Delta | 0.002 | 0.905 | 0.007 | 0.088 | 1.811 | 0.864 | 0.004 | 0.132 | 1.303 |
|  |  | MC |  | 0.915 | 0.004 | 0.081 | 1.729 | 0.865 | 0.002 | 0.133 | 1.266 |
|  | WF | Delta |  | 0.891 | 0.031 | 0.077 | 2.008 | 0.873 | 0.004 | 0.123 | 1.210 |
|  |  | MC |  | 0.898 | 0.012 | 0.089 | 2.078 | 0.893 | 0.002 | 0.105 | 1.195 |
|  | PMS | Delta |  | 0.794 | 0.029 | 0.177 | 2.930 | 0.828 | 0.029 | 0.143 | 2.346 |
|  |  | MC |  | 0.909 | 0.005 | 0.086 | 2.505 | 0.921 | 0.006 | 0.073 | 2.122 |
|  | PTPRS | Delta |  | 0.916 | 0.039 | 0.045 | 2.569 | 0.942 | 0.029 | 0.029 | 1.968 |
|  | NCS | Delta |  | 0.552 | 0.000 | 0.448 | 1.520 | 0.402 | 0.000 | 0.598 | 1.134 |
|  | WnoF | Delta | 0.005 | 0.916 | 0.009 | 0.075 | 1.333 | 0.895 | 0.005 | 0.100 | 0.953 |
|  |  | MC |  | 0.929 | 0.005 | 0.066 | 1.297 | 0.901 | 0.004 | 0.095 | 0.936 |
|  | Weibull | Delta |  | 0.855 | 0.054 | 0.091 | 1.614 | 0.901 | 0.011 | 0.088 | 0.886 |
|  |  | MC |  | 0.890 | 0.025 | 0.085 | 1.702 | 0.914 | 0.009 | 0.077 | 0.881 |
|  | PMS | Delta |  | 0.863 | 0.045 | 0.092 | 2.043 | 0.853 | 0.109 | 0.038 | 1.608 |
|  |  | MC |  | 0.937 | 0.010 | 0.053 | 1.830 | 0.925 | 0.051 | 0.024 | 1.491 |
|  | PTPRS | Delta |  | 0.934 | 0.036 | 0.030 | 1.701 | 0.950 | 0.030 | 0.020 | 1.251 |
|  | NCS | Delta |  | 0.742 | 0.002 | 0.256 | 1.214 | 0.657 | 0.000 | 0.343 | 0.896 |
|  | WnoF | Delta | 0.010 | 0.930 | 0.010 | 0.060 | 1.036 | 0.927 | 0.009 | 0.064 | 0.738 |
|  |  | MC |  | 0.942 | 0.007 | 0.051 | 1.017 | 0.930 | 0.008 | 0.062 | 0.729 |
|  | Weibull | Delta |  | 0.830 | 0.078 | 0.092 | 1.248 | 0.914 | 0.021 | 0.065 | 0.688 |
|  |  | MC |  | 0.885 | 0.040 | 0.075 | 1.436 | 0.923 | 0.018 | 0.059 | 0.688 |
|  | PMS | Delta |  | 0.884 | 0.071 | 0.045 | 1.455 | 0.632 | 0.355 | 0.013 | 0.988 |
|  |  | MC |  | 0.939 | 0.029 | 0.033 | 1.369 | 0.734 | 0.256 | 0.010 | 0.991 |
|  | PTPRS | Delta |  | 0.940 | 0.032 | 0.028 | 1.221 | 0.951 | 0.032 | 0.017 | 0.885 |
|  | NCS | Delta |  | 0.854 | 0.004 | 0.142 | 1.008 | 0.814 | 0.001 | 0.185 | 0.737 |
| 30% | WnoF | Delta | 0.002 | 0.877 | 0.010 | 0.113 | 1.952 | 0.859 | 0.005 | 0.136 | 1.412 |
|  |  | MC |  | 0.904 | 0.003 | 0.093 | 1.855 | 0.865 | 0.001 | 0.134 | 1.373 |
|  | Weibull | Delta |  | 0.901 | 0.017 | 0.082 | 1.835 | 0.845 | 0.076 | 0.079 | 1.807 |
|  |  | MC |  | 0.922 | 0.009 | 0.069 | 1.762 | 0.831 | 0.039 | 0.129 | 1.967 |
|  | PMS | Delta |  | 0.832 | 0.060 | 0.108 | 2.980 | 0.851 | 0.054 | 0.095 | 2.245 |
|  |  | MC |  | 0.941 | 0.012 | 0.047 | 2.531 | 0.936 | 0.009 | 0.055 | 1.993 |
|  | PTPRS | Delta |  | 0.915 | 0.037 | 0.048 | 2.607 | 0.931 | 0.036 | 0.033 | 1.982 |
|  | NCS | Delta |  | 0.682 | 0.002 | 0.316 | 1.819 | 0.613 | 0.000 | 0.387 | 1.370 |
|  | WnoF | Delta | 0.005 | 0.902 | 0.010 | 0.088 | 1.425 | 0.889 | 0.005 | 0.106 | 1.022 |
|  |  | MC |  | 0.920 | 0.005 | 0.075 | 1.372 | 0.898 | 0.005 | 0.097 | 0.998 |
|  | Weibull | Delta |  | 0.913 | 0.022 | 0.065 | 1.346 | 0.716 | 0.160 | 0.124 | 1.464 |
|  |  | MC |  | 0.934 | 0.013 | 0.053 | 1.319 | 0.741 | 0.102 | 0.157 | 1.686 |
|  | PMS | Delta |  | 0.884 | 0.064 | 0.052 | 2.008 | 0.874 | 0.087 | 0.039 | 1.444 |
|  |  | MC |  | 0.942 | 0.020 | 0.038 | 1.823 | 0.931 | 0.043 | 0.026 | 1.362 |
|  | PTPRS | Delta |  | 0.939 | 0.034 | 0.027 | 1.721 | 0.941 | 0.036 | 0.023 | 1.261 |
|  | NCS | Delta |  | 0.827 | 0.006 | 0.167 | 1.411 | 0.779 | 0.002 | 0.219 | 1.045 |
|  | WnoF | Delta | 0.010 | 0.919 | 0.012 | 0.069 | 1.100 | 0.917 | 0.007 | 0.076 | 0.785 |
|  |  | MC |  | 0.930 | 0.008 | 0.062 | 1.068 | 0.920 | 0.005 | 0.075 | 0.769 |
|  | Weibull | Delta |  | 0.924 | 0.025 | 0.051 | 1.045 | 0.634 | 0.212 | 0.154 | 1.210 |
|  |  | MC |  | 0.944 | 0.018 | 0.038 | 1.040 | 0.695 | 0.146 | 0.159 | 1.479 |
|  | PMS | Delta |  | 0.896 | 0.071 | 0.033 | 1.408 | 0.837 | 0.146 | 0.018 | 0.973 |
|  |  | MC |  | 0.944 | 0.031 | 0.026 | 1.351 | 0.882 | 0.100 | 0.018 | 0.955 |
|  | PTPRS | Delta |  | 0.949 | 0.032 | 0.019 | 1.237 | 0.947 | 0.034 | 0.019 | 0.896 |
|  | NCS | Delta |  | 0.891 | 0.009 | 0.100 | 1.142 | 0.887 | 0.009 | 0.104 | 0.836 |
| 60% | WnoF | Delta | 0.002 | 0.894 | 0.014 | 0.092 | 2.179 | 0.896 | 0.009 | 0.095 | 1.596 |
|  |  | MC |  | 0.931 | 0.003 | 0.066 | 2.065 | 0.911 | 0.004 | 0.085 | 1.557 |
|  | Weibull | Delta |  | 0.910 | 0.020 | 0.070 | 2.083 | 0.880 | 0.070 | 0.049 | 1.811 |
|  |  | MC |  | 0.939 | 0.010 | 0.051 | 1.935 | 0.905 | 0.030 | 0.065 | 2.003 |
|  | PMS | Delta |  | 0.822 | 0.105 | 0.073 | 2.845 | 0.883 | 0.079 | 0.038 | 2.146 |
|  |  | MC |  | 0.944 | 0.018 | 0.037 | 2.533 | 0.943 | 0.028 | 0.028 | 2.000 |
|  | PTPRS | Delta |  | 0.900 | 0.042 | 0.058 | 2.551 | 0.928 | 0.042 | 0.030 | 2.055 |
|  | NCS | Delta |  | 0.812 | 0.019 | 0.169 | 2.292 | 0.804 | 0.013 | 0.183 | 1.745 |
|  | WnoF | Delta | 0.005 | 0.915 | 0.015 | 0.070 | 1.570 | 0.917 | 0.012 | 0.071 | 1.136 |
|  |  | MC |  | 0.941 | 0.007 | 0.052 | 1.520 | 0.935 | 0.006 | 0.059 | 1.124 |
|  | Weibull | Delta |  | 0.924 | 0.022 | 0.054 | 1.509 | 0.822 | 0.127 | 0.051 | 1.367 |
|  |  | MC |  | 0.941 | 0.014 | 0.045 | 1.432 | 0.881 | 0.065 | 0.054 | 1.608 |
|  | PMS | Delta |  | 0.874 | 0.086 | 0.040 | 1.872 | 0.897 | 0.085 | 0.017 | 1.355 |
|  |  | MC |  | 0.941 | 0.024 | 0.034 | 1.805 | 0.945 | 0.033 | 0.022 | 1.339 |
|  | PTPRS | Delta |  | 0.922 | 0.040 | 0.038 | 1.695 | 0.944 | 0.035 | 0.021 | 1.285 |
|  | NCS | Delta |  | 0.878 | 0.031 | 0.091 | 1.692 | 0.898 | 0.019 | 0.083 | 1.259 |
|  | WnoF | Delta | 0.010 | 0.931 | 0.018 | 0.051 | 1.193 | 0.939 | 0.012 | 0.049 | 0.856 |
|  |  | MC |  | 0.950 | 0.011 | 0.039 | 1.174 | 0.956 | 0.010 | 0.034 | 0.859 |
|  | Weibull | Delta |  | 0.929 | 0.025 | 0.046 | 1.149 | 0.786 | 0.163 | 0.051 | 1.039 |
|  |  | MC |  | 0.945 | 0.017 | 0.038 | 1.113 | 0.867 | 0.090 | 0.043 | 1.325 |
|  | PMS | Delta |  | 0.916 | 0.059 | 0.025 | 1.294 | 0.919 | 0.069 | 0.012 | 0.913 |
|  |  | MC |  | 0.945 | 0.033 | 0.021 | 1.306 | 0.938 | 0.045 | 0.017 | 0.923 |
|  | PTPRS | Delta |  | 0.937 | 0.033 | 0.030 | 1.231 | 0.945 | 0.035 | 0.020 | 0.909 |
|  | NCS | Delta |  | 0.905 | 0.037 | 0.058 | 1.301 | 0.932 | 0.032 | 0.036 | 0.953 |
| 90% | WnoF | Delta | 0.002 | 0.894 | 0.045 | 0.061 | 2.621 | 0.928 | 0.030 | 0.042 | 1.929 |
|  |  | MC |  | 0.930 | 0.014 | 0.056 | 2.502 | 0.939 | 0.013 | 0.048 | 1.862 |
|  | Weibull | Delta |  | 0.894 | 0.046 | 0.060 | 2.597 | 0.927 | 0.031 | 0.042 | 1.911 |
|  |  | MC |  | 0.944 | 0.012 | 0.044 | 2.536 | 0.947 | 0.012 | 0.041 | 1.870 |
|  | PMS | Delta |  | 0.834 | 0.096 | 0.069 | 2.839 | 0.892 | 0.072 | 0.036 | 2.172 |
|  |  | MC |  | 0.947 | 0.020 | 0.033 | 2.848 | 0.946 | 0.031 | 0.023 | 2.172 |
|  | PTPRS | Delta |  | 0.868 | 0.062 | 0.070 | 2.645 | 0.913 | 0.045 | 0.042 | 2.017 |
|  | NCS | Delta |  | 0.826 | 0.103 | 0.071 | 2.597 | 0.872 | 0.079 | 0.049 | 1.978 |
|  | WnoF | Delta | 0.005 | 0.910 | 0.040 | 0.050 | 1.841 | 0.938 | 0.026 | 0.036 | 1.319 |
|  |  | MC |  | 0.937 | 0.019 | 0.044 | 1.829 | 0.947 | 0.018 | 0.035 | 1.308 |
|  | Weibull | Delta |  | 0.911 | 0.040 | 0.049 | 1.827 | 0.938 | 0.027 | 0.035 | 1.310 |
|  |  | MC |  | 0.941 | 0.017 | 0.042 | 1.882 | 0.945 | 0.019 | 0.036 | 1.322 |
|  | PMS | Delta |  | 0.893 | 0.070 | 0.036 | 1.920 | 0.927 | 0.057 | 0.016 | 1.365 |
|  |  | MC |  | 0.942 | 0.028 | 0.030 | 2.141 | 0.952 | 0.029 | 0.019 | 1.493 |
|  | PTPRS | Delta |  | 0.901 | 0.052 | 0.047 | 1.827 | 0.937 | 0.040 | 0.023 | 1.309 |
|  | NCS | Delta |  | 0.892 | 0.068 | 0.040 | 1.849 | 0.915 | 0.059 | 0.026 | 1.346 |
|  | WnoF | Delta | 0.010 | 0.927 | 0.025 | 0.048 | 1.376 | 0.946 | 0.030 | 0.024 | 0.967 |
|  |  | MC |  | 0.940 | 0.018 | 0.042 | 1.429 | 0.950 | 0.024 | 0.026 | 0.989 |
|  | Weibull | Delta |  | 0.927 | 0.026 | 0.047 | 1.368 | 0.945 | 0.031 | 0.024 | 0.962 |
|  |  | MC |  | 0.943 | 0.019 | 0.038 | 1.491 | 0.951 | 0.024 | 0.025 | 0.999 |
|  | PMS | Delta |  | 0.920 | 0.054 | 0.026 | 1.367 | 0.941 | 0.040 | 0.019 | 0.965 |
|  |  | MC |  | 0.951 | 0.022 | 0.027 | 1.778 | 0.950 | 0.027 | 0.023 | 1.219 |
|  | PTPRS | Delta |  | 0.922 | 0.037 | 0.041 | 1.347 | 0.941 | 0.035 | 0.024 | 0.959 |
|  | NCS | Delta |  | 0.920 | 0.047 | 0.033 | 1.355 | 0.941 | 0.040 | 0.019 | 0.971 |

Note: Metrics are calculated based on the logarithms of TTB estimates. The number of MC samples is 2,000. ARR: absolute relative risk; CP: coverage probability; OLLP: out-of-lower-limit probability; OULP: out-of-upper-limit probability; MAE: mean absolute error; RMSE: root mean squared error; MC: Monte Carlo; NCS: natural cubic spline; PMS: penalized M-spline; PTPRS: penalized thin plate regression spline; SPRINT: Systolic Blood Pressure Intervention Trial; TTB: time to benefit; WF: Weibull model with shared frailty; WnoF: Weibull model without shared frailty

## Supplemenraty Figures


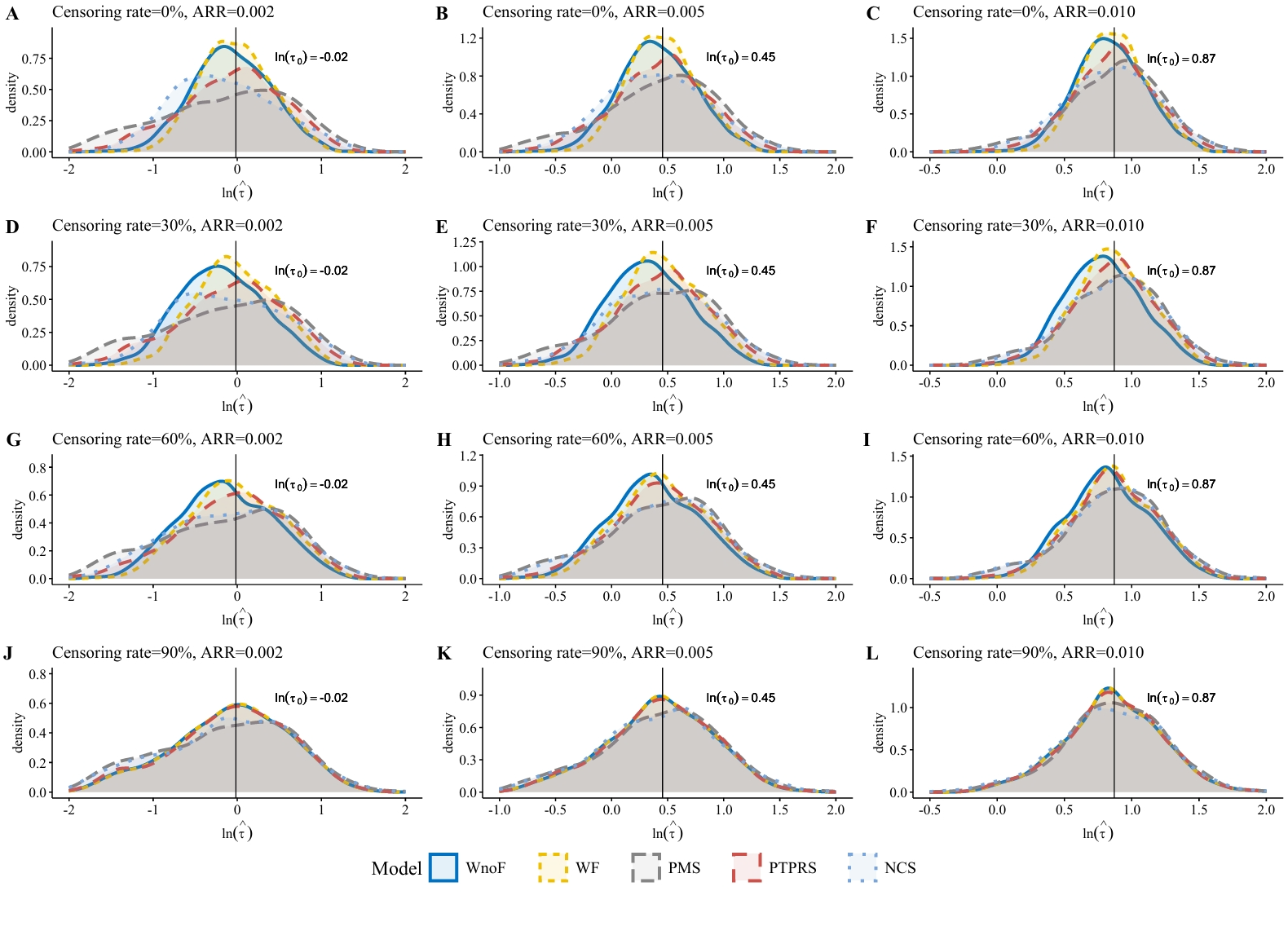


**Figure S1.** The sampling distributions of the logarithms of TTB estimates for five models in the simulation scenario I with the Gamma frailty. The kernel density estimates are demonstrated. The sample size is 5,000 and the number of simulation replication is 1,000. Vertical lines denote the true logarithms of TTB. ARR: absolute relative risk; MC: Monte Carlo; NCS: natural cubic spline; PMS: penalized M-spline; PTPRS: penalized thin plate regression spline; TTB: time to benefit; WF: Weibull model with shared frailty; WnoF: Weibull model without shared frailty


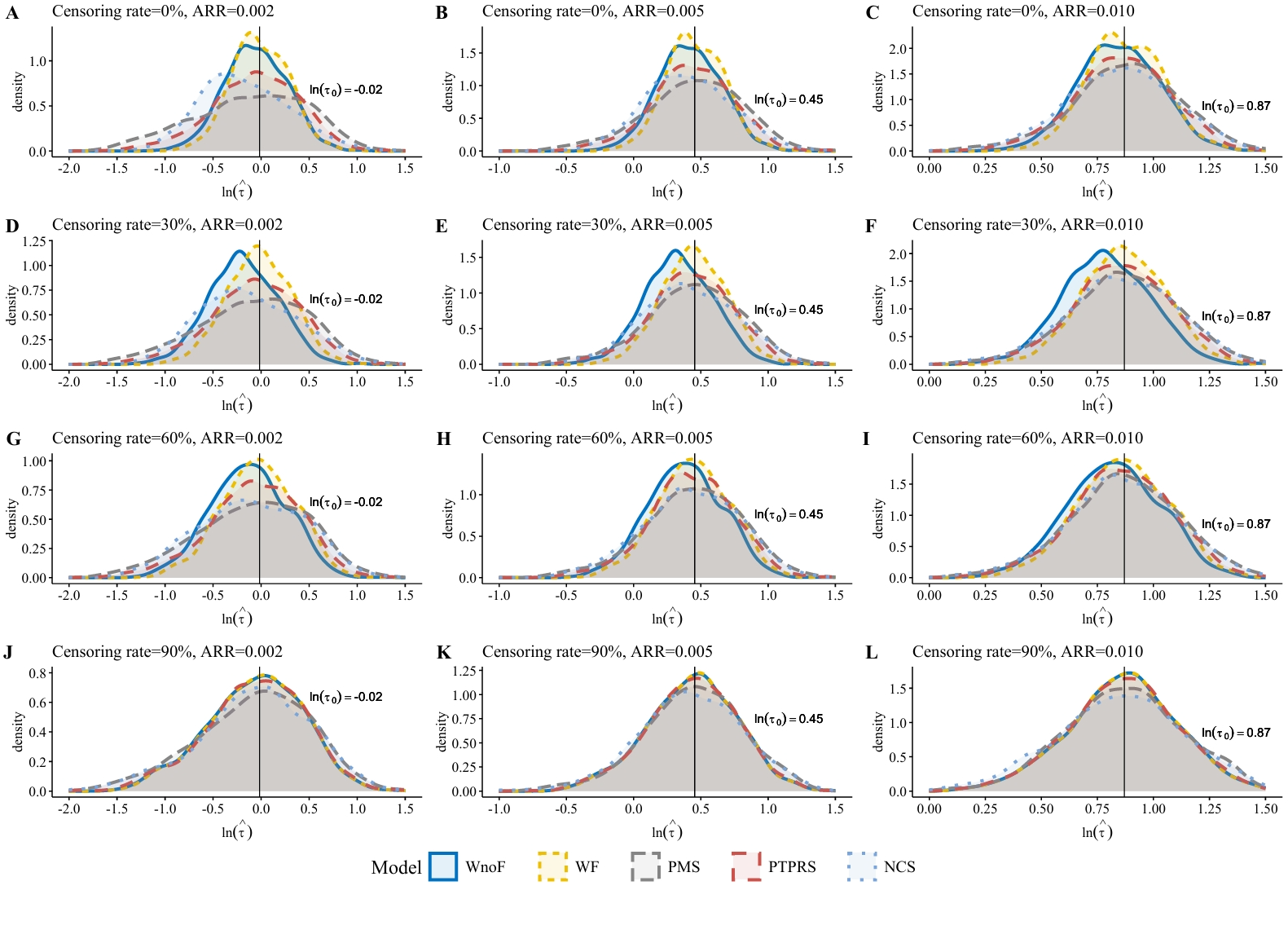
**Figure S2.** The sampling distributions of the logarithms of TTB estimates for five models in the simulation scenario I with the Gamma frailty. The kernel density estimates are demonstrated. The sample size is 10,000 and the number of simulation replication is 1,000. Vertical lines denote the true logarithms of TTB. ARR: absolute relative risk; MC: Monte Carlo; NCS: natural cubic spline; PMS: penalized M-spline; PTPRS: penalized thin plate regression spline; TTB: time to benefit; WF: Weibull model with shared frailty; WnoF: Weibull model without shared frailty


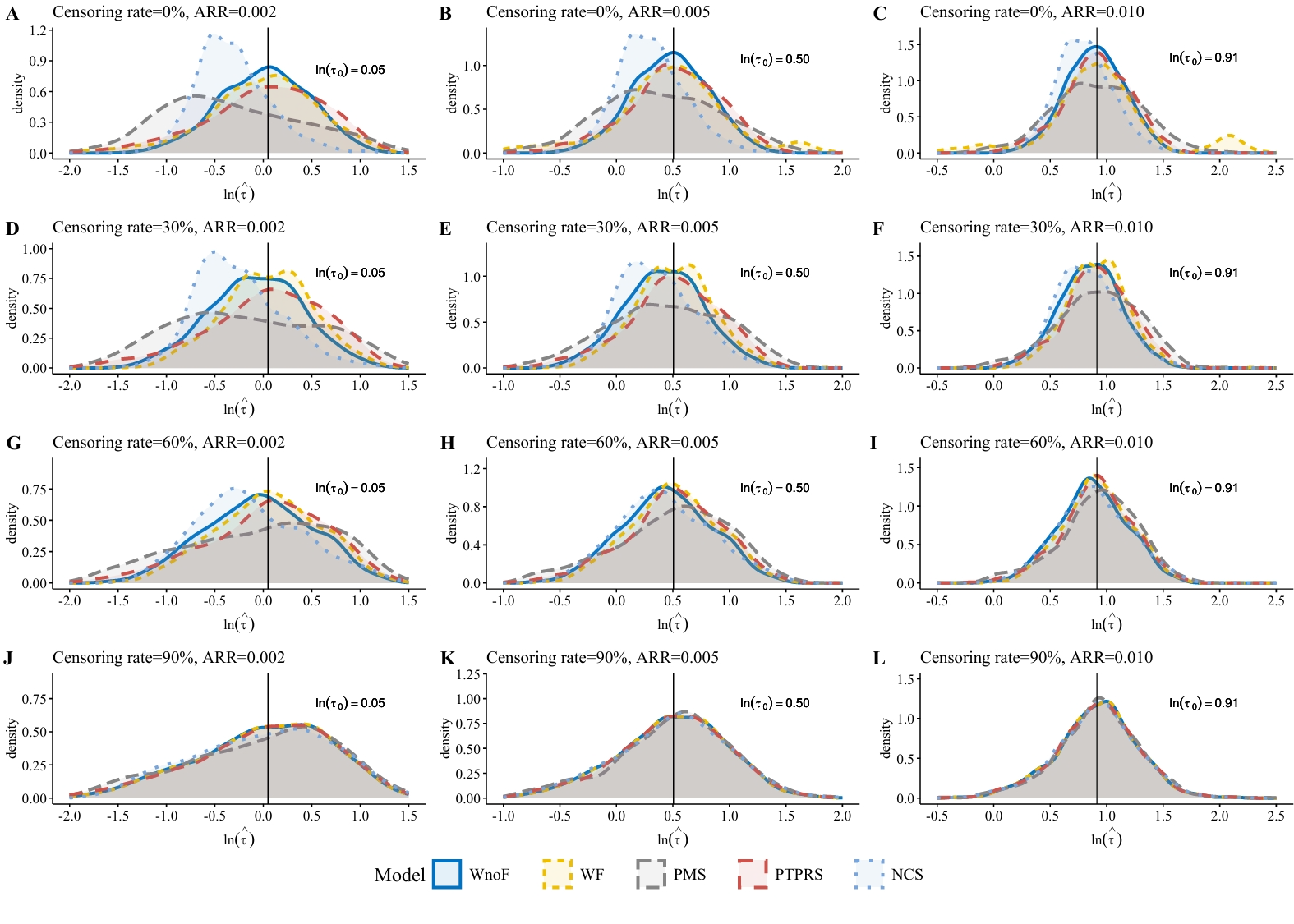
**Figure S3.** The sampling distributions of the logarithms of TTB estimates for five models in the simulation scenario I with the log-normal frailty. The kernel density estimates are demonstrated. The sample size is 5,000 and the number of simulation replication is 1,000. Vertical lines denote the true logarithms of TTB. ARR: absolute relative risk; MC: Monte Carlo; NCS: natural cubic spline; PMS: penalized M-spline; PTPRS: penalized thin plate regression spline; TTB: time to benefit; WF: Weibull model with shared frailty; WnoF: Weibull model without shared frailty


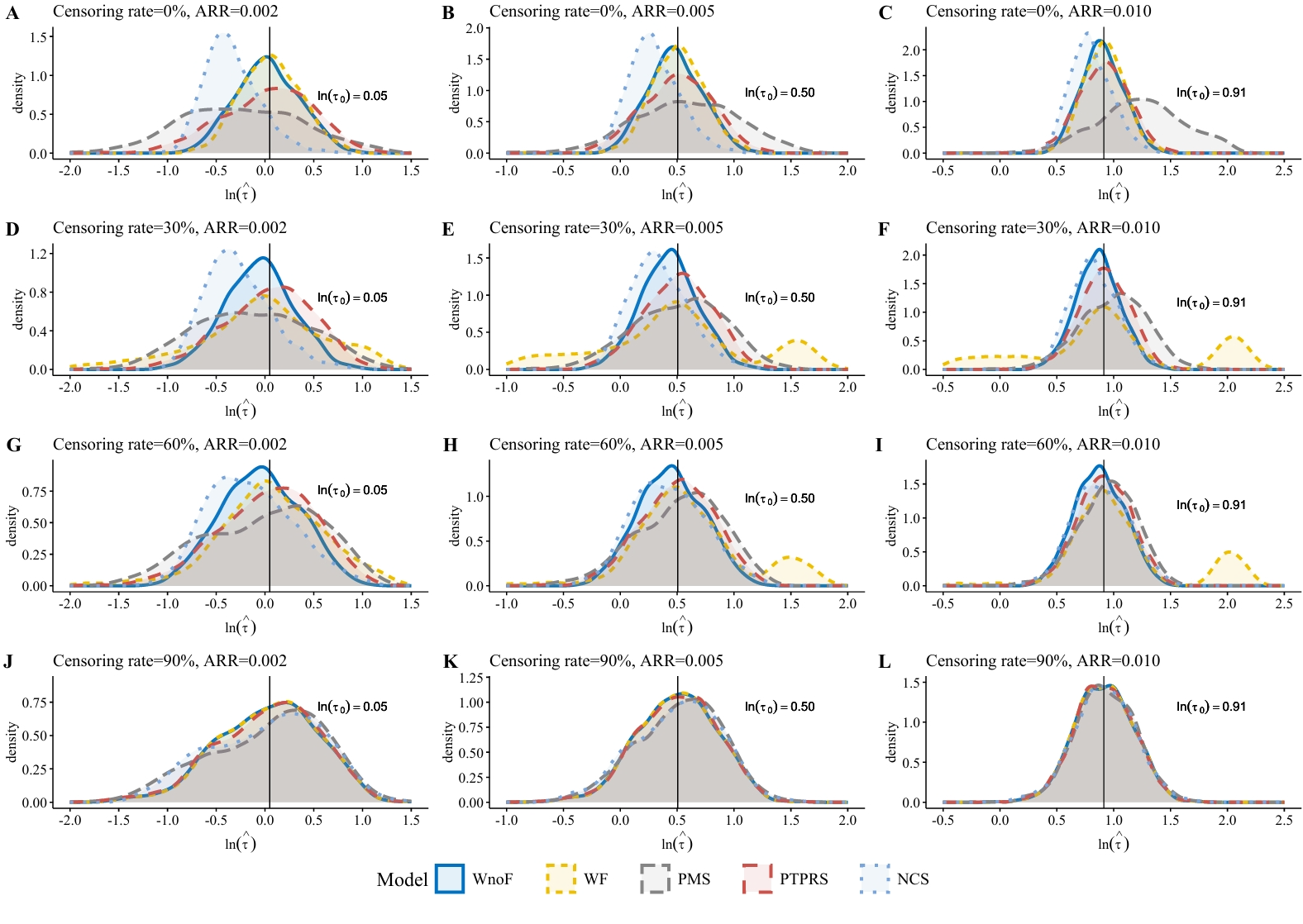
**Figure S4.** The sampling distributions of the logarithms of TTB estimates for five models in the simulation scenario I with the log-normal frailty. The kernel density estimates are demonstrated. The sample size is 10,000 and the number of simulation replication is 1,000. Vertical lines denote the true logarithms of TTB. ARR: absolute relative risk; MC: Monte Carlo; NCS: natural cubic spline; PMS: penalized M-spline; PTPRS: penalized thin plate regression spline; TTB: time to benefit; WF: Weibull model with shared frailty; WnoF: Weibull model without shared frailty
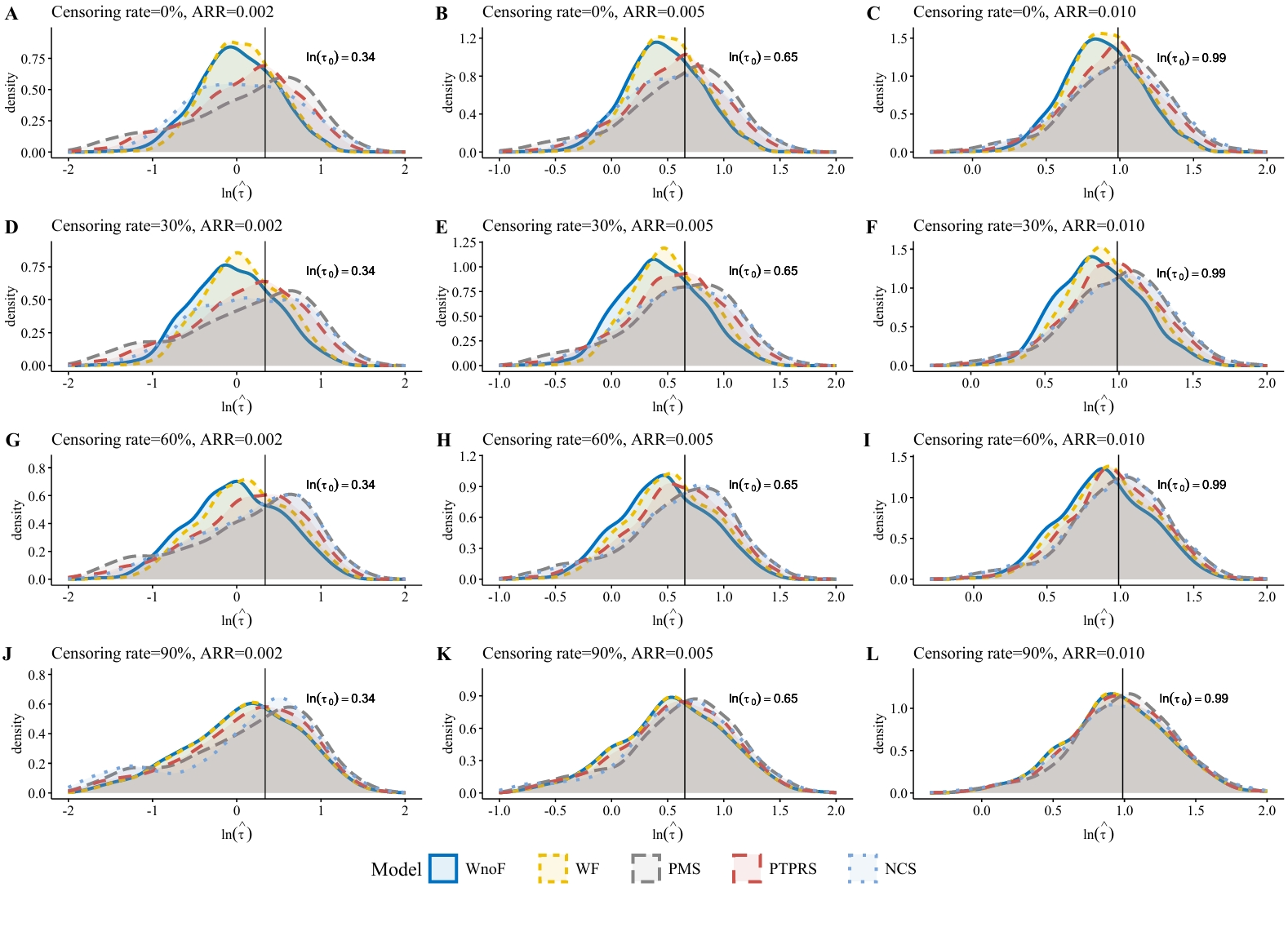


**Figure S5.** The sampling distributions of the logarithms of TTB estimates for five models in the simulation scenario II with the Gamma frailty. The kernel density estimates are demonstrated. The sample size is 5,000 and the number of simulation replication is 1,000. Vertical lines denote the true logarithms of TTB. ARR: absolute relative risk; MC: Monte Carlo; NCS: natural cubic spline; PMS: penalized M-spline; PTPRS: penalized thin plate regression spline; TTB: time to benefit; WF: Weibull model with shared frailty; WnoF: Weibull model without shared frailty


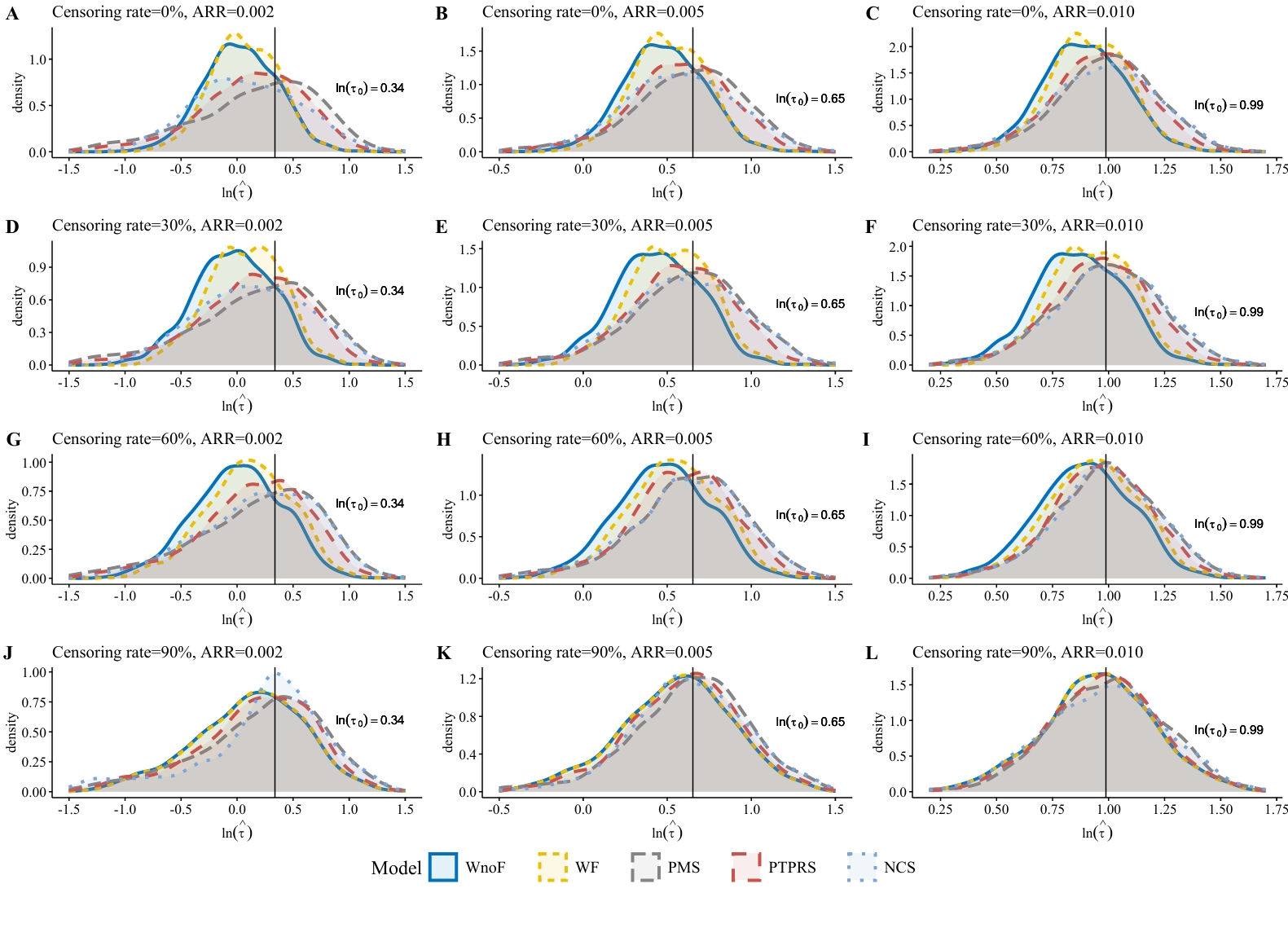
 **Figure S6.** The sampling distributions of the logarithms of TTB estimates for five models in the simulation scenario II with the Gamma frailty. The kernel density estimates are demonstrated. The sample size is 10,000 and the number of simulation replication is 1,000. Vertical lines denote the true logarithms of TTB. ARR: absolute relative risk; MC: Monte Carlo; NCS: natural cubic spline; PMS: penalized M-spline; PTPRS: penalized thin plate regression spline; TTB: time to benefit; WF: Weibull model with shared frailty; WnoF: Weibull model without shared frailty


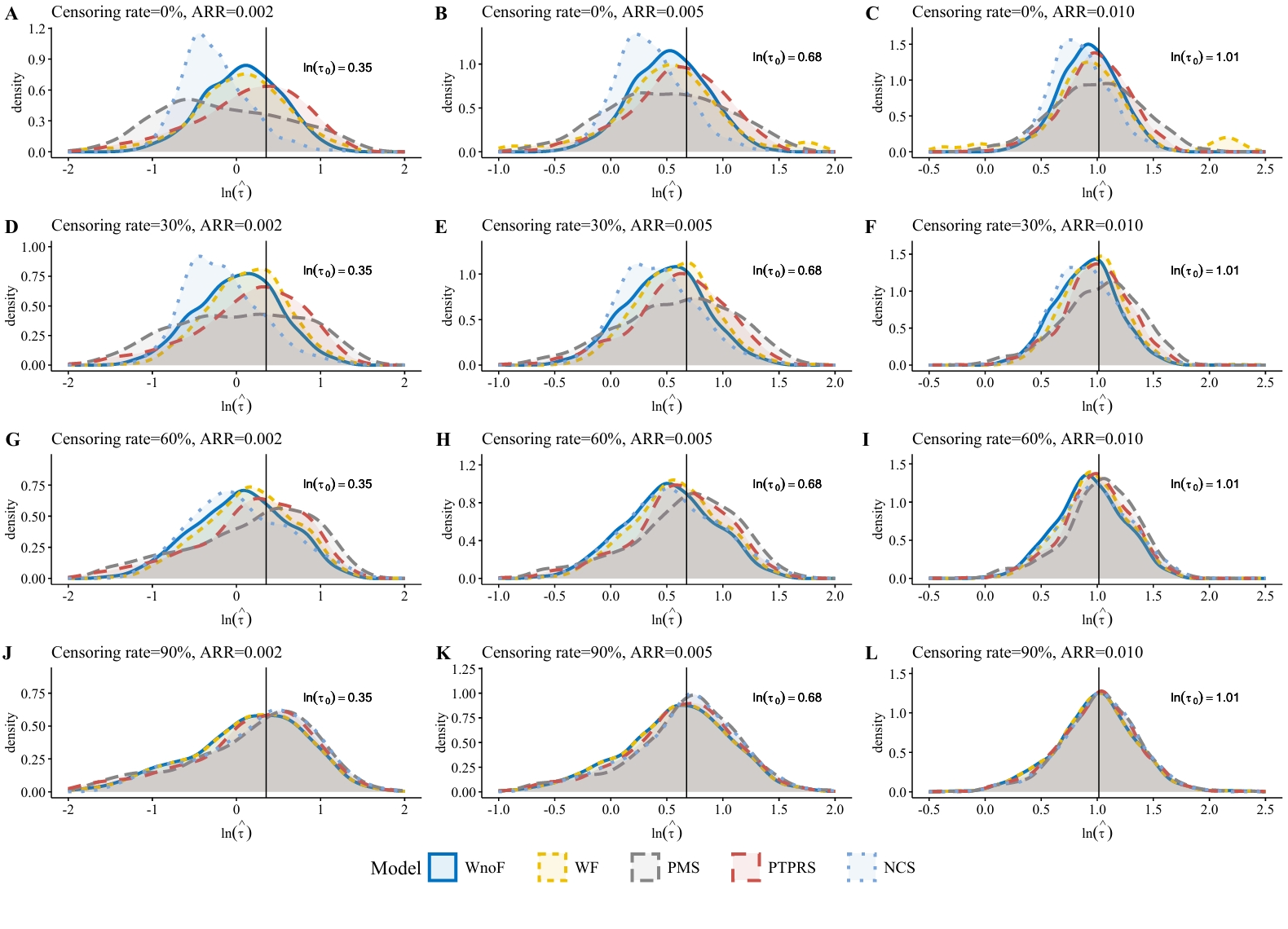


**Figure S7.** The sampling distributions of the logarithms of TTB estimates for five models in the simulation scenario II with the log-normal frailty. The kernel density estimates are demonstrated. The sample size is 5,000 and the number of simulation replication is 1,000. Vertical lines denote the true logarithms of TTB. ARR: absolute relative risk; MC: Monte Carlo; NCS: natural cubic spline; PMS: penalized M-spline; PTPRS: penalized thin plate regression spline; TTB: time to benefit; WF: Weibull model with shared frailty; WnoF: Weibull model without shared frailty

**
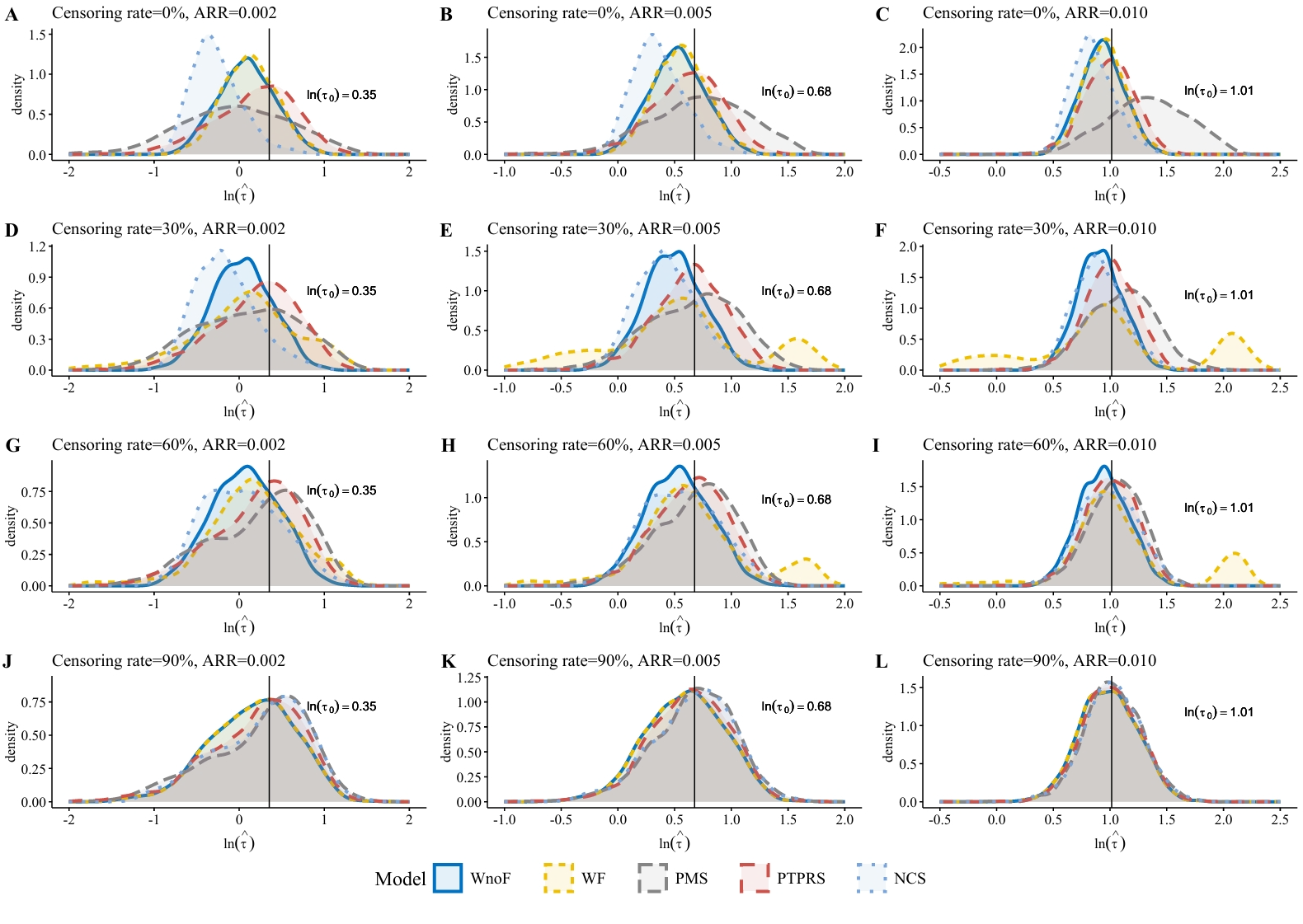
Figure S8.** The sampling distributions of the logarithms of TTB estimates for five models in the simulation scenario II with the log-normal frailty. The kernel density estimates are demonstrated. The sample size is 10,000 and the number of simulation replication is 1,000. Vertical lines denote the true logarithms of TTB. ARR: absolute relative risk; MC: Monte Carlo; NCS: natural cubic spline; PMS: penalized M-spline; PTPRS: penalized thin plate regression spline; TTB: time to benefit; WF: Weibull model with shared frailty; WnoF: Weibull model without shared frailty


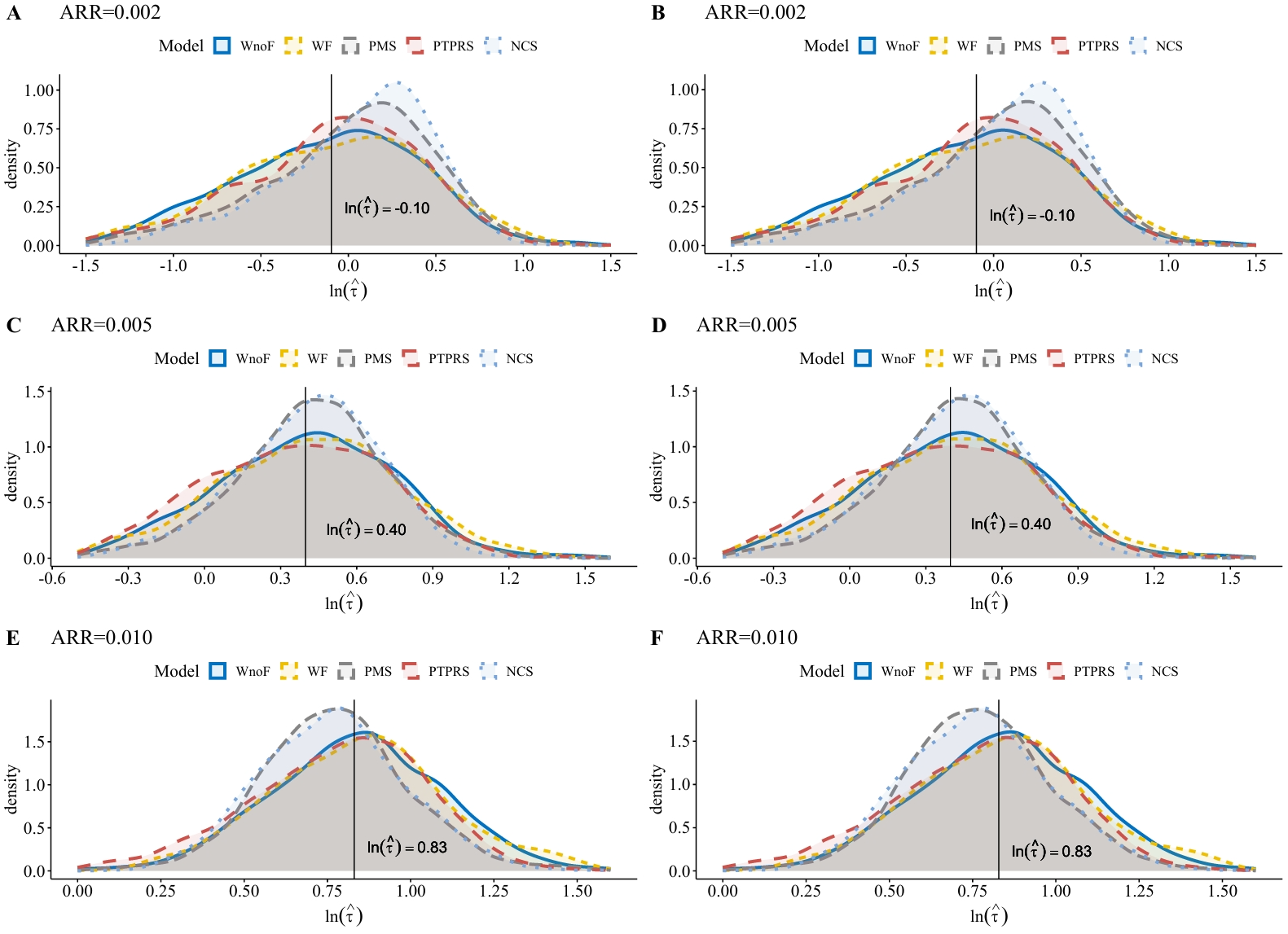


**Figure S9.** The empirical sampling distributions of the logarithms of TTB estimates for five models with the Gamma frailty and 1,000 MC samples for SPRINT dataset. The kernel density estimates are demonstrated. Left Panel ACE are results for the five models with the Gamma frailty. Right Panel BDF are results for the five models with the log-normal frailty. Vertical lines denote the estimated logarithms of TTB by the Weibull model with the Delta method. ARR: absolute relative risk; MC: Monte Carlo; NCS: natural cubic spline; PMS: penalized M-spline; PTPRS: penalized thin plate regression spline; SPRINT: Systolic Blood Pressure Intervention Trial; TTB: time to benefit; WF: Weibull model with shared frailty; WnoF: Weibull model without shared frailty.
